# An alternative framework for fluorescence correlation spectroscopy

**DOI:** 10.1038/s41467-019-11574-2

**Published:** 2019-08-14

**Authors:** Sina Jazani, Ioannis Sgouralis, Omer M. Shafraz, Marcia Levitus, Sanjeevi Sivasankar, Steve Pressé

**Affiliations:** 1 Center for Biological Physics, Arizona State University, Tempe, AZ 85287 USA; 2 Department of Physics, Arizona State University, Tempe, AZ 85287 USA; 3 Department of Biomedical Engineering, University of California, Davis, CA 95616 USA; 4 School of Molecular Sciences, Arizona State University, Tempe, AZ 85287 USA; 5 Biodesign Institute, Arizona State University, Tempe, AZ 85287 USA

**Keywords:** Biophysical methods, Microscopy, Optical spectroscopy, Biological fluorescence

## Abstract

Fluorescence correlation spectroscopy (FCS), is a widely used tool routinely exploited for in vivo and in vitro applications. While FCS provides estimates of dynamical quantities, such as diffusion coefficients, it demands high signal to noise ratios and long time traces, typically in the minute range. In principle, the same information can be extracted from microseconds to seconds long time traces; however, an appropriate analysis method is missing. To overcome these limitations, we adapt novel tools inspired by Bayesian non-parametrics, which starts from the direct analysis of the observed photon counts. With this approach, we are able to analyze time traces, which are too short to be analyzed by existing methods, including FCS. Our new analysis extends the capability of single molecule fluorescence confocal microscopy approaches to probe processes several orders of magnitude faster and permits a reduction of photo-toxic effects on living samples induced by long periods of light exposure.

## Abstract (web summary)

Fluorescence correlation spectroscopy is widely used for in vivo and in vitro applications, yet extracting information from experiments still requires long acquisition times. Here, the authors exploit Bayesian non-parametrics to directly analyze the output of confocal fluorescence experiments thereby probing physical processes on much faster timescales.

## Introduction

Owing to its flexibility and limited invasiveness for in vivo applications, single-molecule fluorescence confocal microscopy^1–4^ is one of the most widely used experimental techniques of modern biophysics. In this technique, fluorescent molecules are allowed to freely diffuse into a volume illuminated by a tightly focused laser beam of a conventional single-focus confocal setup. Molecular motion inside the illuminated volume generates fluctuations in the emitted fluorescence that is recorded and subsequently temporally autocorrelated^1–4^ or, jointly spatiotemporally autocorrelated^5–7^, to deduce physical quantities of interest. For example, fluorescence correlation spectroscopy (FCS)^1,2^ as well as complementary methods—such as fluorescence cross-correlation spectroscopy (FCCS)^8^, scanning FCS^9,10^, spot variation fluorescence correlation spectroscopy^11^, fluorescence resonance energy transfer-fluorescence correlation spectroscopy (FRET-FCS)^12,13^, etc—estimate diffusion coefficients, reaction kinetic, binding affinities, and concentrations of labeled molecules^14,15^.

Although single-molecule fluorescence confocal microscopy data are acquired on the nano- to millisecond timescales (ns-ms), fluorescence correlation methods typically require the analysis of long time traces, several seconds to tens of minutes long, depending on the molecular concentrations and emission properties of the fluorophores employed^16,17^. These traces, capturing multiple molecule traversals of the confocal volume, provide the statistics needed for the post-processing steps used in traditional FCS analysis^16^ (e.g. autocorrelation, and nonlinear fitting to theoretical curves). However, processing steps like these downgrade the quality of the available data and demand either relatively high concentrations or excessively long time traces to yield reliable estimates. The same downgrades are encountered even with less-traditional FCS analyses, including Bayesian approaches^18–22^, which also rely on auto-correlations.

In principle, within milliseconds, for the fluorophore concentrations and confocal volumes used in most experiments^1,2,23^, thousands of data points are already available. Accordingly, if one could, somehow, estimate diffusion coefficients within tens of ms with the same accuracy as FCS, one could hypothetically use tens of minutes worth of data to discriminate between very small differences in diffusion coefficients. Alternatively, one could opt for shorter traces in the first place and, in doing so, reduce the sample’s light exposure to only a few milliseconds, thereby minimizing photo-toxic effects, which remain a severe limitation of fluorescence microscopy^24–26^.

Exploiting data on millisecond timescales would require a method that, simultaneously and self-consistently, estimates the number of fluorescent molecules at any given time within the (inhomogenously) illuminated volume and deduce their dynamical properties based on their photon emissions, which, in turn, depend on their evolving location within the confocal volume. The mathematics to do so in a rigorous and efficient manner have, so far, been unavailable as analyzing ms traces would demand that we consider all possible populations of molecules responsible for the observed traces, their diffusion coefficients, and every possible location (and, thus, photon emission rate) of those molecules at any given time.

Indeed, with current technology, this global optimization is prohibitively computationally expensive. To wit, maximum likelihood approaches^15,27^, popular in a variety of applications, are excluded as they require that the, otherwise unknown, population of molecules in the confocal volume at any given time be specified in advance by other means. These considerations motivate an entirely new framework for FCS.

Here, we introduce a novel approach that exploits Bayesian non-parametrics^15,28,29^, a branch of statistics first suggested^30^ in 1973 and only broadly popularized in physical applications over the last few years^15,28,29,31–37^. This approach allows us to account for an arbitrary number of molecules responsible for emitting detected photons. With the proposed method, we are able to estimate physical variables, otherwise determined from FCS, with: (i) significantly shorter time traces; and (ii) nearly single-molecule resolution. Furthermore, our overall framework is generalizable and can estimate not only diffusion coefficients and molecular populations but also track molecules through time as well as determine their molecular brightness and the background photon emission rate.

## Results

### Overview

The method we propose for the analysis of traces from single-molecule fluorescence confocal microscopy follows the Bayesian paradigm^15,27,29,38^. Within this paradigm, our goal is to estimate posterior probability distributions over unknown parameters such as diffusion coefficients as well as molecular populations over time.

In this section, we first demonstrate and validate our method by computing posterior distributions using synthetic (simulated) traces mimicking the properties of real single-molecule fluorescence confocal experiments. We subsequently benchmark our estimates with traces from control in vitro experiments. A comprehensive summary of the results can be found in Supplementary Table 1.

### Demonstration and validation with simulated data

**Fig. 1:**
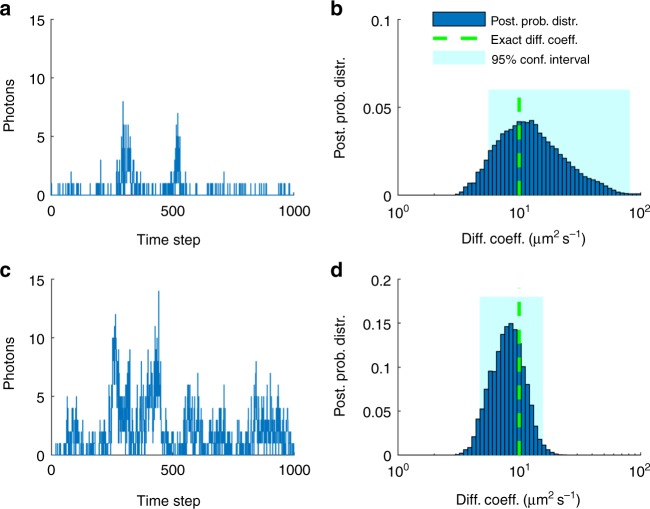
Effects of the number of molecules. **a** Synthetic fluorescent intensity trace produced by one molecule inside the confocal volume. For this simulation we used a molecular brightness of 5×10^4^ photons s^−1^ and a background photon emission rate of 10^3^ photons s^−1^. **b** Posterior probability distribution over the diffusion coefficient estimated from the trace in **a**. **c** Synthetic fluorescent intensity trace produced by five molecules inside the confocal volume otherwise identical to **a**. **d** Posterior probability distribution over the diffusion coefficient estimated from **c**. Traces shown in **a**, **c** are acquired at 100 μs for a total of 100 ms and the highlighted regions in **b** and **d** represent the 95% confidence intervals. For clarity, the horizontal axis is shown in logarithmic scale

**Fig. 2:**
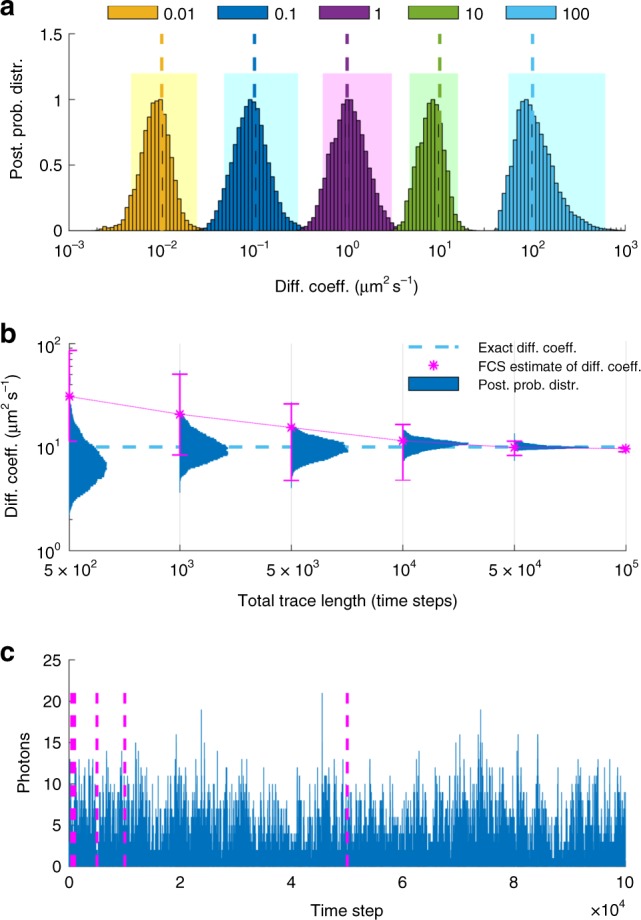
Effects of diffusion coefficient and trace length. **a** Posterior probability distributions deduced from traces produced from molecules with diffusion coefficients of 0.01, 0.1, 1, 10, and 100 μm^2^ s^−1^. For clarity, posteriors are normalized to maximum 1 and the horizontal axis is shown in logarithmic scale. Shaded regions illustrate the 95% confidence intervals. **b** Posterior probability distributions deduced from traces acquired at 100 μs with total trace lengths of 5 × 10^2^, 1 × 10^3^, 5 ×  10^3^, 1 × 10^4^, 5 × 10^4^ time steps. For the sake of comparison, exact values and FCS estimates are also shown and, for clarity, the vertical axis is shown in logarithmic scale. Error bars in the FCS curve are produced by analyzing multiple windows in the initial trace. In order to estimate the diffusion coefficient within less than a factor of 2 of the true value, it is typical for FCS to require ≈ 50 × more data than our method. **c** The entire trace used to deduce diffusion coefficients in **a**, **b**. Each segment, marked by dashed lines, represents the portion used in **b**. The molecular brightness and background photon emission rates used to generate the time traces are 5 × 10^4^ and 10^3^ photons s^−1^

**Fig. 3:**
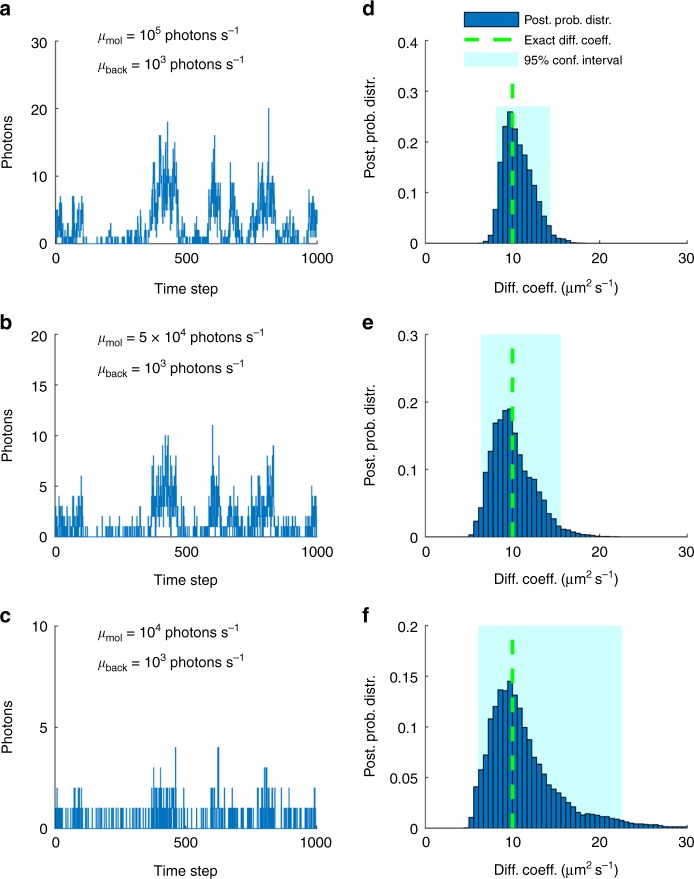
Effects of molecular brightness. **a**–**c** Intensity traces produced by the same molecular trajectories under molecular brightness of 10^5^, 5 × 10^4^, and 1 × 10^4^ photons s^−1^ and background photon emission rate fixed at 10^3^ photons s^−1^. All traces are acquired at 100 μs for a total length of 100 ms. **d**–**f** Posterior probability distributions and exact values of diffusion coefficients obtained from the corresponding traces. Shaded regions illustrate the 95% confidence intervals

To demonstrate the robustness of our method, we simulate fluorescent time traces under a broad range of: (i) numbers of labeled molecules in the effective volume, Fig. 1; (ii) diffusion coefficients, Fig. 2a; (iii) trace lengths, Fig. 2b; and (iv) molecular brightness, Fig. 3. As, the majority of our time traces are too short to be meaningfully analyzed with traditional FCS, we compare our posteriors directly to the ground truth that we used in the simulations.

The posteriors we obtain, in all figures, are informed from the analysis of a single trace. In those, the breadth of the posterior (i.e., variance), which is a measure of the accuracy of our estimate, indicates the uncertainty introduced by the finiteness of the data and the inherent noise in this single time trace.

To begin, in Fig. 1 we simulate a 3D Gaussian confocal volume of size *ω*_xy_ = 0.3 μm and *ω*_z_ = 1.5 μm and one molecule inside the effective volume (Fig. 1a) or five molecules inside the effective volume (Fig. 1c) diffusing at 10 μm^2^ s^−1^ for a total period of 100 ms. The corresponding full joint posteriors are shown in Supplementary Figs 1 and 2.

As can be seen in Fig. 1, a low intensity leads to a wide estimate of the diffusion coefficient. However, the higher the intensity, the sharper (i.e., more conclusive) the estimate of the diffusion coefficient becomes (e.g., note a narrower posterior in Fig. 1d as compared with Fig. 1b). Thus, diffusion coefficients are determined more accurately when the number of labeled molecules are higher. Accordingly, the most difficult data to analyze are those where concentrations of molecules are so low that, on average, only one molecule ventures, albeit rarely, into the effective region of the confocal volume where it can be appreciably excited. Put differently, for an equal length time trace, the posterior estimate over the diffusion coefficient is broader (i.e., less conclusive) for lower numbers of molecules inside the effective volume, Fig. 1b, than it is for larger numbers of molecules, Fig. 1d.

Following a similar reasoning, the slower a molecule diffuses, the more photons are collected, leading to a sharper posterior estimate of the corresponding diffusion coefficient, Fig. 2a. Likewise, the longer the trace is, Fig. 2b, or the greater the molecular brightness is, Fig. 3, the sharper the diffusion coefficient estimate becomes.

We emphasize that our definition of molecular brightness is based on the maximum number of detected photons emitted from a single fluorophore when it is located at the center of the confocal volume and we provide more details in the Supplementary Note 3.

In Fig. 3 we demonstrate the robustness of the diffusion coefficient estimates when varying the molecular brightness. Although we keep the background photon emission rate fixed, we simulate gradually dimmer fluorophores such as those encountered in experiments under lower laser powers, until the molecular signature is virtually lost. As can be seen, such traces lead to broader posterior estimates over diffusion coefficients, as one would expect, as these traces are associated with greater uncertainty. Also, as such traces lead to a weaker (i.e., less constraining) likelihood, the posterior resembles more closely the prior (similar to every Bayesian methods) and naturally starts to deviate from the exact value.

### Experimental data with elongated confocal volume shapes

**Fig. 4:**
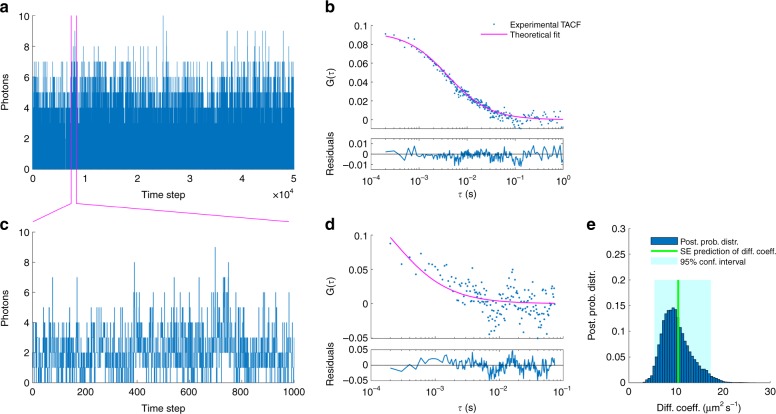
Traces of free fluorescent beads using an elongated confocal volume. **a** Experimental fluorescent intensity trace used in FCS. **b** Autocorrelation curve of the trace in **a** and best theoretical fit. **c** Portion of the trace in **a** to be used as the input to FCS and our method. **d** Autocorrelation curve of trace in **c**. **e** Posterior probability distribution over the diffusion coefficient estimated from the trace in **c**. The Stokes–Einstein prediction is denoted by a green line. Traces shown in **a** and **c** are acquired at 100 μs for a total period of 5 μs and 0.1 μs, respectively. The laser power used to generate the trace **a** is 100 μW (measured before the beam enters the objective). The estimate of diffusion coefficient resulting by autocorrelation fitting in **a** matches with the Stokes–Einstein prediction (i.e., 10.5 μm^2^ s^−1^); whereas, in **d** is almost 10-fold higher (~ 10 μm^2^ s^−1^)

Here, we apply our method on experimental traces captured with an elongated confocal volume that we approximate by a cylinder. To do so, we apply our method on fluorescent beads (with average diameter of 45 nm) diffusing in water. We benchmark our estimated diffusion coefficients against the Stokes–Einstein prediction and results from FCS. In particular, Fig. 4 illustrates our method’s performance in the analysis of traces too short to be meaningfully analyzed by FCS. The FCS formulation we used here can be found in Supplementary Note 3 and additional results can be found in Supplementary Fig. 10.

### Experimental data with elliptical confocal volume shapes

Next, we apply our method on experimental time traces derived from single-molecule fluorescence confocal microscopy. In our setup, we monitor Cy3 dyes, which diffuse freely in a mixture of water and glycerol. We benchmark our estimated diffusion coefficients against two values: those predicted by the Stokes–Einstein formula^39^, which is parametrized by physical quantities such as temperature and viscosity; and those estimated by FCS. To analyze the data using FCS reliably, we used the full (6 min) trace available.

In benchmarking, we obtained and analyzed measurements with different: (i) numbers of Cy3 dyes inside the effective volume (tuned by varying Cy3 concentration); (ii) trace lengths; (ii) diffusion coefficients (tuned by adjusting the viscosity of the solution); and (iii) molecular brightness (tuned by adjusting the laser power).

**Fig. 5:**
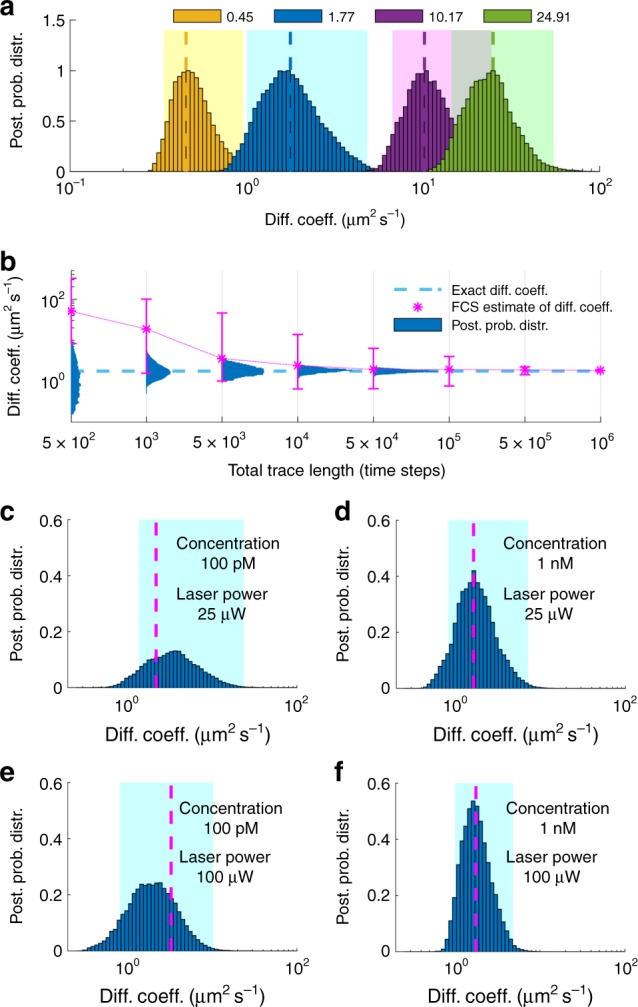
Estimating diffusion coefficients of free Cy3. **a** Posterior probability distributions of diffusion coefficients of free Cy3 in different concentrations of glycerol/water mixture. The legend labels the posteriors according to FCS estimates of long time traces. For clarity, posteriors are normalized to maximum 1 and the horizontal axis is shown in logarithmic scale. Also, the 95% confidence intervals are shown by highlighted regions. Posteriors are obtained from the analyses of time traces acquired at 100 μs for total periods of 100 ms. Different diffusion coefficients are obtained by varying the amount of glycerol from 99 to 50% in the glycerol/water mixture. **b** Posterior probability distributions deduced from traces acquired at 100 μs with total trace lengths of 5 × 10^2^, 1 × 10^3^, 5 × 10^3^, 1 × 10^4^, and 5 × 10^4^ time steps. For the sake of comparison, exact values and FCS estimates are also shown and, for clarity, the vertical axis is shown in logarithmic scale. Error bars in the FCS curve are produced by analyzing multiple windows in the initial trace. To estimate the diffusion coefficient within less than a factor of 2 of the true value, it is typical for FCS to require ≈ 50 × more data than our method. **c**–**f** Posterior probability distributions over the diffusion coefficients of traces generated by different laser powers (25 and 100 μW, respectively) with different concentrations of Cy3 (100 pm and 1 nm, respectively) in a glycerol/water mixture of 94% glycerol. For the sake of comparison, FCS estimates, shown with dashed lines, are obtained from traces each 6 min long

Just as before, the slower a molecule diffuses, the more time it spends in the vicinity of the confocal volume, so the more photons are collected, thereby leading to sharper posterior estimates for the diffusion coefficient; as seen on Fig. 5a.

In Fig. 5b, we illustrate the effect of different time trace length. In this case, we reach the same accuracy as FCS with 100 × times less data. Consistent with the synthetic data shown earlier, we obtain a broader posterior over diffusion coefficients when the number of dyes inside the effective volume is low and sharper posteriors for higher numbers of dyes.

For example, in Fig. 5c–f, we illustrate the effect of different dye concentrations where a trace with stronger signal, anticipated when concentrations are higher, leads to better diffusion coefficient estimates (and thus sharper posteriors) on traces of equal length owing to the higher number of labeled molecules inside the confocal volume. The corresponding full joint posteriors of Fig. 5 are shown in Supplementary Figs 6 and 7.

In general, a posterior’s sharpness depends strongly on the number of molecules in a time trace, their respective locations, and thus their photon emission rates. As the molecular population near the center of the confocal volume may exhibit strong fluctuations, the width of the posterior may also fluctuate from trace to trace, especially when the individual traces are short. Thus, the individual posteriors become sharper only on average as we move to higher numbers of molecules inside the effective volume or molecular brightness.

**Fig. 6:**
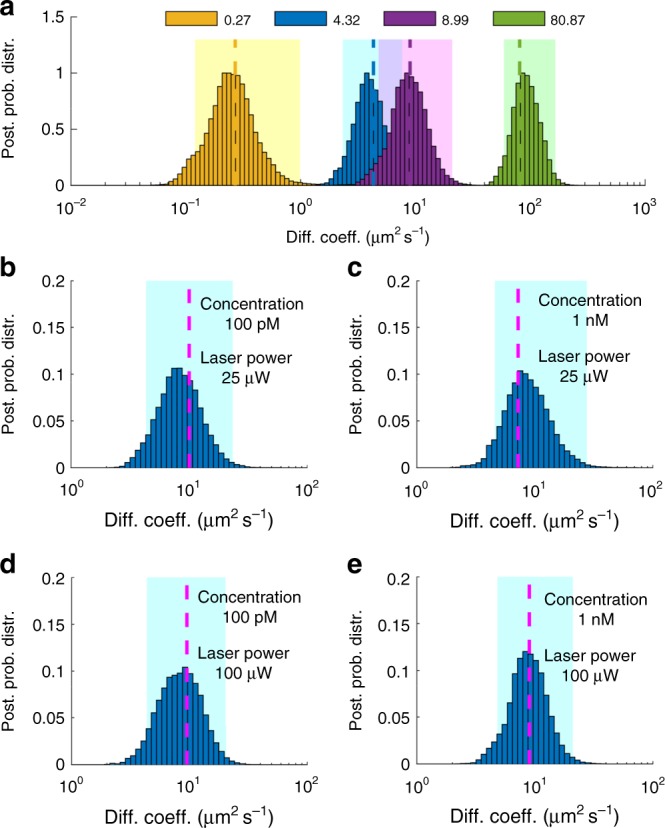
Estimating diffusion coefficients of free streptavidin. **a** Posterior probability distributions of diffusion coefficients of free streptavidin labeled by Cy3 in different concentrations of glycerol/water mixture. The legend labels the posteriors according to FCS estimates of long time traces. For clarity, posteriors are normalized to maximum 1 and the horizontal axis is shown in logarithmic scale. Also, the 95% confidence intervals are shown by highlighted regions. Posteriors are obtained from the analyses of time traces acquired at 100 μs for total periods of 100 ms. Different diffusion coefficients are obtained by varying the amount of glycerol from 94 to 0% in the glycerol/water mixture. **b**–**e** Posterior probability distributions over the diffusion coefficients of traces generated by different laser powers (25 and 100 μW, respectively) with different concentrations of Cy3 (100 pm and 1 nm, respectively) in a glycerol/water mixture of 94% glycerol. For the sake of comparison, FCS estimates, shown by dashed lines, are obtained by traces each 6 min long

To test our method beyond free beads and dyes, we used labeled proteins, namely freely diffusing streptavidin labeled by Cy3. Similar to the previous cases, we tested a range of concentrations, diffusion coefficients, and laser powers. Figure 6 summarizes characteristic results and compares our analyses against the results of FCS, which is applied on longer time traces (6 min). As can be seen, even in this case our method provides acceptable estimates of the diffusion coefficient with 100 × times fewer data points than FCS.

### Additional results

**Fig. 7:**
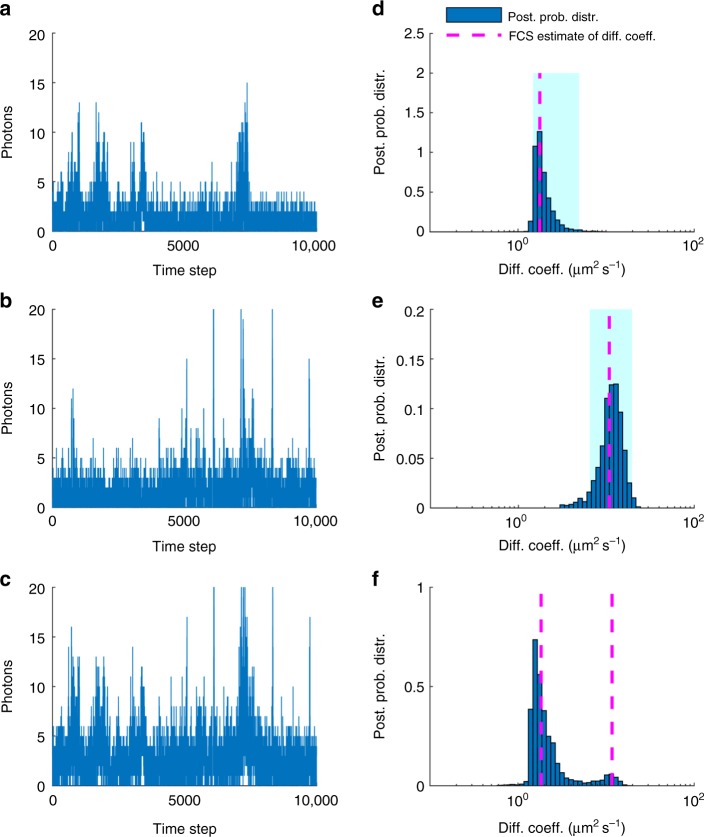
Estimating multiple diffusion coefficients in Cy3 traces. **a**, **b** Experimental traces of free Cy3 in glycerol/water mixtures with 94% and 75% glycerol, respectively. **c** Trace resulting by mixing the traces in **a** and **b**. **d**, **e** Posterior probability distributions resulting from the analysis of the traces in **a** and **b**. **f** Posterior probability distribution resulting from the analysis of the trace in **c**. For comparison, FCS estimates, shown by dashed lines, are produced from five different traces, each of 6 min, i.e., ≈ 100 × longer than the segments shown and analyzed in our method. Posteriors are obtained from the analyses of time traces acquired at 100 μs for a total period of 1 μs

In addition to cases involving a single diffusion coefficient that we have considered thus far, our method can be generalized to treat multiple diffusion coefficients as well. To show this, we artificially mixed (summed) and analyzed experimental traces where dyes diffuse in different amounts of glycerol and so they exhibit different diffusion coefficients. On account of the additivity of photon emissions and detections, artificial mixing of traces allows us to obtain realistic traces of different diffusive species that can be analyzed as if they were diffusing simultaneously within the same confocal volume and separately as well. In Fig. 7, we compare the analysis of intensities created by mixing traces containing slow and fast diffusing Cy3 (94% and 75% glycerol/water, respectively). As can be seen, our estimates obtained under simultaneous diffusion compare favorably to the estimates under separate diffusion, indicating that our method can also identify robustly multiple diffusion coefficients at once. The full joint posterior distributions corresponding to Fig. 7 and additional data are shown in Supplementary Figs 12 and 13.

For all cases described so far, using our method, we estimated more than just diffusion coefficients. For example, we also estimate the population of molecules contributing photons to the traces, their instantaneous photon emission rates and locations relative to the center of the confocal volume, as well as the background photon emission rate. A more-detailed report of our estimates, with presentations of full joint posterior distributions, can be found in the Supplementary Figs 3, 4, 8, and 9.

## Discussion

Single-molecule fluorescence confocal microscopy has the potential to reveal dynamical information at timescales that may be as short as a hundred milliseconds. Here, we have exploited Bayesian non-parametrics to overcome the limitations of specifically fluorescent correlative methods in utilizing short, ≈ 10 ms, and noisy time traces to deduce molecular properties such as diffusion coefficients. Exploiting new analysis, to obtain reliable results from such short traces or excessively noisy traces as those obtained under low laser power, is key to minimizing photo-damage inherent to all methods relying on illumination and especially critical to gaining insight on rapid or light-sensitive processes^24,26^. The analysis of similarly short traces is also required when monitoring non-equilibrium processes that remain stationary or approximately stationary over only short periods of time. Furthermore, novel analysis with increased sensitivity may exploit the entirety of longer traces to tease out subtle dynamical features (such as deducing multiple diffusion coefficients at once).

The deep implication of our method is that it places single-molecule fluorescence confocal microscopy at a competitive advantage over wide-field techniques used in single particle tracking. Indeed, wide-field techniques provide high, super-resolved, spatial accuracy^15^, but with diminished temporal resolution, as molecule localization requires the collection of sufficient photons obtained only after long frame exposures^15^. Such a requirement is especially problematic for photo-sensitive or rapidly diffusing biomolecules^15^.

By contrast to wide-field microscopy, single-molecule fluorescence confocal microscopy yields minimal spatial resolution. However, as our analysis shows, although spatial resolution may be diminished, reduced photo-damage and exceptionally high temporal resolution can be achieved instead.

Since their inception, over half a century ago, correlative methods, such as FCS, have demanded very long traces in order to extract dynamical features from single-molecule fluorescence confocal microscopy data^2,11,40–43^. In this study, we have developed a principled framework capable of taking advantage of all spatio-temporal information nested within time traces of photon counts and, together with novel mathematics, we have reformulated the analysis of single-molecule fluorescence confocal microscopy data.

Existing methods, even those that apply Bayesian techniques such as FCS-Bayes^18–22^, still utilize autocorrelation functions. Therefore, they demand equally long time traces as FCS and implicitly assume that the molecular process probed (e.g., diffusion) remains stationary over the portion of the trace analyzed. By contrast, our method only requires short traces and therefore it avoids stationarity or equilibrium requirements on timescales longer than those of the segments analyzed. In addition, our method also: (i) provides interpretable estimation of errors (i.e., posterior variance) determined exclusively from the information content of the trace supplied (i.e., length and noise) as opposed to ad hoc metrics residuals (i.e., chi square); (ii) tracks instantaneous molecule photon emissions and locations; and (iii) estimates the molecular brightness and background photon emission rates which, if left undetermined, can introduce biases.

As our method is formulated exclusively in the time-domain, it offers a versatile framework for further modifications. For example, it is possible to adapt the present formulation to incorporate scanning FCS^9,10,44^ which involves moving the confocal volume or incorporate demanding illumination profiles, such as those arising in two photon excitation^42,45^, TIRF microscopy^41^ or even Airy patterns^46^ with or without aberrations^47^ by changing the specified point spread function (see Methods section). In addition, it is possible to extend our framework to treat multiple diffusion coefficients (see Supplementary Note 5), confining forces or photon emission kinetics as would be relevant for FCS-FRET^48,49^ and FLIM^50,51^ applications. Also, our method could be extended to handle more complex photophysics^23,52–54^, and, as we explicitly track individual molecules over time, extensions appropriate for fast bimolecular reaction kinetics are also conceivable.

## Methods

### Model overview

Here we describe the formulation and mathematical foundation of our model. Our overarching goal is to start from an experimental time series of photon counts, $\bar w = (w_1,w_2,...,w_K)$ where *w*_*k*_ denotes the photon intensity assessed at time *t*_*k*_ (which includes both background photons as well as photons derived from the labeled molecules of interest), and derive estimates of kinetic quantities such as molecular locations with respect to the center of the confocal volume as well as diffusion coefficients.

To derive estimates for the desired quantities, we need to compute intermediate quantities which include: (i) molecular brightness; (ii) background photon emission rate; and, most importantly, (iii) the unknown population of moving molecules and their relative locations with respect to the center of the confocal volume. Below we explain each one of these in detail. Computational details and a working implementation of the entire method are available in the Supplementary Notes 3 and 4. For convenience, we summarize our notation, abbreviations and mathematical definitions in Supplementary Tables 2–4.

### Model description

The starting point of our analysis is the raw data, namely the photon counts. As our current focus is on deducing dynamical information on timescales exceeding ≈ 1 μs, we ignore triplet state and photon anti-bunching effects that occur on vastly different timescales^16,55,56^.

At the timescale of interest, individual photon detections, assuming saturation is not reached, happen stochastically and independently from each other. Accordingly, the total number of photon counts *w*_*k*_ between successive assessments follows Poisson^15,27^ (shot noise) statistics

1 $$\begin{array}{*{20}{l}} {w_k\sim {\mathrm{Poisson}}\left( {(t_k - t_{k - 1})\left( {\mu _{back} + \mathop {\sum}\limits_n {\mu _k^n} } \right)} \right)} \hfill \end{array}$$

where *μ*_back_ is a background photon emission rate and $\mathop {\sum}\limits_n {\mu _k^n}$ gathers the photon emission rates $\mu _k^n$ from individual fluorescent molecules that we index with *n* = 1, 2, …. The number of molecules involved in the summation above is to be determined. This is the key reason we invoke Bayesian non-parametrics in the model inference section (see below). As we only collect a small fraction of the total photons emitted by the fluorescent molecules, as we describe above, in our framework $\mu _k^n$ coincides with the emission rate of detected photons, as opposed to the true photon emission rate, which might be larger.

Each rate $\mu _k^n$ depends on the position $(x_k^n,y_k^n,z_k^n)$ of the corresponding molecule relative to the center of the confocal volume as well as other features such as laser intensity, laser wavelength, quantum yield, and camera pinhole size^57^. Similar to other studies^40,58,59^, we combine all these effects into a characteristic point spread function (PSF) that combines excitation and emission PSFs

2 $$\mu _k^n = \mu _{{\mathrm{mol}}}{\mathrm{PSF}}(x_k^n,y_k^n,z_k^n).$$

The parameter *μ*_mol_ represents the molecular brightness and, as we discuss in the Supplementary Note 3, it is related to the maximum photon emission rate of a single molecule that is located at the center of the confocal volume. Specific choices of PSF models, such as Gaussian or Gaussian-Lorentzian, are also detailed in the Supplementary Note 3 and a comparison of the different PSF models is shown in Supplementary Fig. 5.

**Fig. 8:**
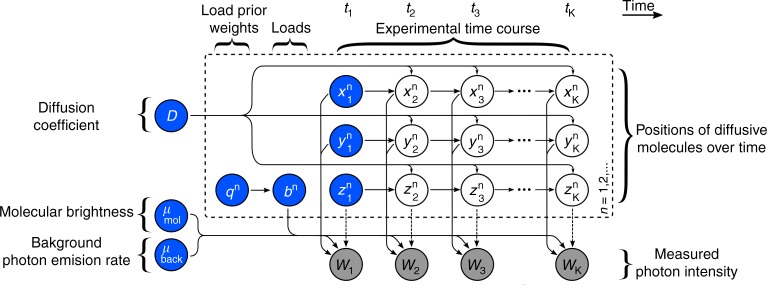
Graphical representation of the formulation. A population of model molecules, labeled by *n* = 1, 2,…, evolves over the course of the experiment that is marked by *k* = 1, 2, …, *K*. Here, $x_k^n$, $y_k^n$, $z_k^n$ denote the location, in Cartesian space, of molecule *n* at time *t*_*k*_; *μ*_mol_ denotes the brightness of an individual molecule; and *μ*_back_ denotes the background photon emission rate. During the experiment, only a single observation *w*_*k*_, combining photon emissions between *t*_*k*−1_ and *t*_*k*_ from every molecule and background is recorded at every time step. The diffusion coefficient *D* determines the evolution of the molecular locations which, in turn, influence the photon emission rates and ultimately the recorded photon intensity *w*_*k*_. Auxiliary variables *b*^*n*^, or “loads”, and corresponding prior weights *q*^*n*^, are introduced in order to estimate the unknown population size. The dashed arrows apply for the 3D Gaussian and 2D-Gaussian-Lorentzian PSFs; while, in the case of the 2D-Gaussian-Cylindrical, there is no dependency of the measurements *w*_*k*_ on the $z_k^n$ coordinates of the molecules (see the Supplementary Note 3 for the definitions of the PSFs)

Finally, we associate individual molecular locations across time by adopting a motion model. Here we assume that molecules are purely diffusive and arrive at

3 $$\begin{array}{l}x_k^n\sim {\mathrm{Normal}}\left( {x_{k - 1}^n,2(t_k - t_{k - 1})D} \right)\\ y_k^n\sim {\mathrm{Normal}}\left( {y_{k - 1}^n,2(t_k - t_{k - 1})D} \right)\\ z_k^n\sim {\mathrm{Normal}}\left( {z_{k - 1}^n,2(t_k - t_{k - 1})D} \right)\end{array}$$

where *D* denotes the diffusion coefficient, which we assume is the same for all molecules. As we explain in the Supplementary Note 4, these probabilities result directly from the diffusion equation. A graphical summary of the entire formulation is shown on Fig. 8.

In addition, in the Supplementary Note 5 and Supplementary Fig. 16, we illustrate how this motion model can be generalized to capture more than one diffusion coefficients.

### Model inference

The quantities that we want to estimate, for example the diffusion coefficient *D*, molecular locations through time $(x_k^n,y_k^n,z_k^n)$, molecular brightness *μ*_mol_ and background photon emission rate *μ*_back_, or the molecular population, are introduced as model variables in the preceding formulation. To estimate values for these variables, we follow the Bayesian paradigm^15,28,38,59^.

Variables such as *D*, *μ*_mol_, and *μ*_back_ are parameters of the model and, as such, require priors. Choices for these priors are straightforward and, for interpretational and computational convenience, we adopt the distributions described in the Supplementary Note 4.

In addition, we must place priors on the initial molecular locations, $(x_1^n,y_1^n,z_1^n)$, i.e., the locations of the molecules at the onset of the measurement period. Specifying a prior on initial molecular locations also entails specifying a prior on the molecular population.

In particular, to allow the dimensionality or, alternatively, the complexity of our model to fluctuate based on the number of molecules that contribute to the fluorescent trace, we abandon traditional Bayesian parametric priors and turn to the non-parametric formulation described below.

Before we proceed any further, we recast Eq. (2) as

4 $$\mu _k^n = b^n\mu _{{\mathrm{mol}}}PSF(x_k^n,y_k^n,z_k^n).$$

The newly introduced variables *b*^*n*^, one for each model molecule, may take only values 1 or 0. In particular, the possibility that *b*^*n*^ = 0, coinciding with the case where molecules do not contribute to the observation, allows us to introduce an arbitrarily large number of molecules, technically an infinite number. With the introduction of *b*^*n*^, we can estimate the number of molecules that contribute photons (termed “active” to distinguish them from those that do not contribute termed “inactive”) simultaneously with the rest of the parameters simply by treating each *b*^*n*^ as a separate parameter and estimating its value (of 1 for active molecules and 0 for inactive ones).

To estimate *b*^*n*^, we place a prior *b*^*n*^ ~ Bernoulli(*q*^*n*^) and subsequently a hyperprior on *q*^*n*^ in order to learn precisely how many model molecules are active. For the latter, we choose *q*^*n*^ ~ Beta(*A*_*q*_, *B*_*q*_) with hyperparameters *A*_*q*_ and *B*_*q*_. Both steps can be combined by invoking the newly developed Beta-Bernoulli process^36,60^ which is described in more detail in the Supplementary Note 4.

Once the choices for the priors above are made, we form a joint posterior probability distribution $p(D,\mu _{{\mathrm{mol}}},\mu _{{\mathrm{back}}},\{ x_k^n,y_k^n,z_k^n,b^n,q^n\} _k^n|\bar w)$ encompassing all unknown variables which we may wish to determine.

The nonlinearities in the PSF with respect to variables $\{ x_k^n,y_k^n,z_k^n\} _k^n$ and the non-parametric prior on {*b*^*n*^, *q*^*n*^}^*n*^ exclude analytic forms for our posterior. For this reason, we develop a computational scheme exploiting Markov chain Monte Carlo^38,61^ that can be used to generate pseudo-random samples from this posterior.

The main bottleneck of a naive implementation of our method, as compared with correlative methods, is its higher computational cost. As we explain in the Supplementary Note 4, to have computations run on an average desktop computer, we adopt mathematical approximations (e.g., photon binning, Anscombe transform^62^ and filter updates^63,64^) that are tested on the synthetic data presented. Specifically, the time trace preparation is described in the Supplementary Note 3 and Supplementary Fig. 14.

A working implementation of the framework described in this study is provided in the source code and the graphical user interface (GUI) is shown on Supplementary Fig. 15.

### Data acquisition

*Synthetic data:* We obtain the synthetic data presented in the Results section by standard pseudo-random computer simulations^65–67^ that mimic the common single-molecule fluorescence confocal setup. We provide details and complete parameter choices in the Supplementary Note 3, Supplementary Tables 5 and 6.

*Experimental data:* For the experimental data acquired with elongated confocal volumes, a stock solution of Cy3B (mono-reactive NHS ester, GE Healthcare) was prepared by dissolving a small amount of solid in 1 mL of doubly-distilled water, and its concentration was determined from the absorbance of the solution using the extinction coefficient provided by the vendors. A 10 nm solution was then prepared by appropriate dilution of the stock and measured on a silicone perfusion chamber mounted on a glass coverslip. Fluorescent beads were purchased from ThermoFisher (Catalog number: F8792. Lot number: 1604237). The average diameter was 0.046 μm as indicated in the certificate of analysis provided by the vendors. Suspensions for FCS measurements were prepared by adding 3 μL of stock solution (9.4 × 1014 particles/mL) to 1 mL of water and sonicating the mixture for 20 min. Measurements were carried out using a home-built instrument. A 532 nm continuous-wave laser (Compass 215M-10, Coherent, Santa Clara, CA) was attenuated to 100 μW and focused onto an PlanApo 100 ×, 1.4 NA, oil-immersion, objective (Olympus, Center Valley, PA). Emitted fluorescence was collected using the same objective and then passed through a 50 μm pinhole to reject the out-of-focus light. The signal was detected using a silicon avalanche photodiode (SPCM-AQR-14; Perkin-Elmer, Fremont, CA). A bandpass filter (Omega 3RD560-620) in front of the detector was employed to further reduce the background signal and an ALV correlator card (ALV 5000/EPP, ALV-GmbH, Langen, Germany) was used to correlate the detected fluorescence signal. Data for our analysis were acquired with 100 μs resolution using a PCI-6602 acquisition card (National Instruments, Austin, TX). Measurements have been tested for saturation separately and shown on Supplementary Fig. 11.

For the experimental data acquired with elliptical confocal volumes, Cy3 dye and Cy3-labeled streptavidin solutions were prepared by suspending Cy3 or streptavidin in glycerol/buffer (pH 7.5, 10 mm Tris-HCl,100 mm NaCl and 10 mm KCl, 2.5 mm CaCl_2_) at different v/v, to a final concentration of either 100 pm or 1 nm. The solutions were added onto a glass-bottomed fluid-cell, mounted on a custom designed single-molecule fluorescence confocal microscope^68,69^ and a 532 nm laser beam was focused to a diffraction-limited spot on the glass coverslip of the fluid-cell using a × 60, 1.42 NA, oil-immersion objective (Olympus). The laser power was measured before the objective and the beam was reflected by a dichroic and focused by the objective on to the sample. The dichroic reflected 95% of the intensity on to the objective. Emitted fluorescence was collected by the same objective and focused onto the detection face of a Single Photon Avalanche Diode (SPAD, Micro Photon Devices) that has a maximum count rate of 11.8 Mc/s. A bandpass filter was placed in front of the detector to transmit only the fluorescence from Cy3 and to block the back-scattered excitation light. TTL pulses, triggered by the arrival of individual photons on the SPAD, were timestamped and recorded at 80 MHz by a field programmable gated array (FPGA, NI Instruments) using custom LabVIEW software^69^ and initially binned at 100 μs.

## Supplementary information

Supplementary Information

## Data Availability

The data generated and analyzed in this study are freely available from the corresponding author upon request.

## Code availability

Source code and GUI versions of the methods developed herewith are freely available to download at [https://cbp.asu.edu/content/steve-presse-lab] or [https://github.com/sinajazani/F-CS].
