# Supplementary material for: An alternative framework for fluorescence correlation spectroscopy
Source: Nat Commun. 2019 Aug 14;10:3662. doi: 10.1038/s41467-019-11574-2 (PMC6694112; doi:10.1038/s41467-019-11574-2)
Supplement: Supplementary file 1 — Supplementary Information [file 41467_2019_11574_MOESM1_ESM.pdf]

An Alternative Framework for Fluorescence Correlation Spectroscopy — Supplementary Information—

Sina Jazani and Ioannis Sgouralis
Center for Biological Physics, Department of Physics
Arizona State University, Tempe, AZ 85287

Omer M. Shafraz and Sanjeevi Sivasankar
Department of Biomedical Engineering, University of California, Davis, CA 95616

Marcia Levitus
Center for Biological Physics,
School of Molecular Sciences and Biodesign Institute
Arizona State University, Tempe, AZ 85287

Steve Pressé
Center for Biological Physics,
Department of Physics and School of Molecular Sciences
Arizona State University, Tempe, AZ 85287

Here we provide supplementary materials and technical details that complement the main text. These include: (i) Additional analysis results that demonstrate the estimation of molecular brightness and background photon emission rates, joint posterior probability distributions, molecule locations, and additional results for multiple diffusive species. These results are repeated for simulated and experimental data. (ii) Additional details of the methods used including descriptions of the motion model, the Stokes-Einstein model, point spread functions (PSFs), various definitions, and time trace preparation. (iii) A complete description of the inference framework developed that includes choices for the prior probability distributions and a computational implementation. (iv) A description of the modifications necessary for the model with multiple diffusive species. (v) Summary of notation and other conventions used throughout this study as well as detailed parameter choices for the simulations and analyses.

Supplementary Note 1: Additional results

1. Analysis of additional simulated data

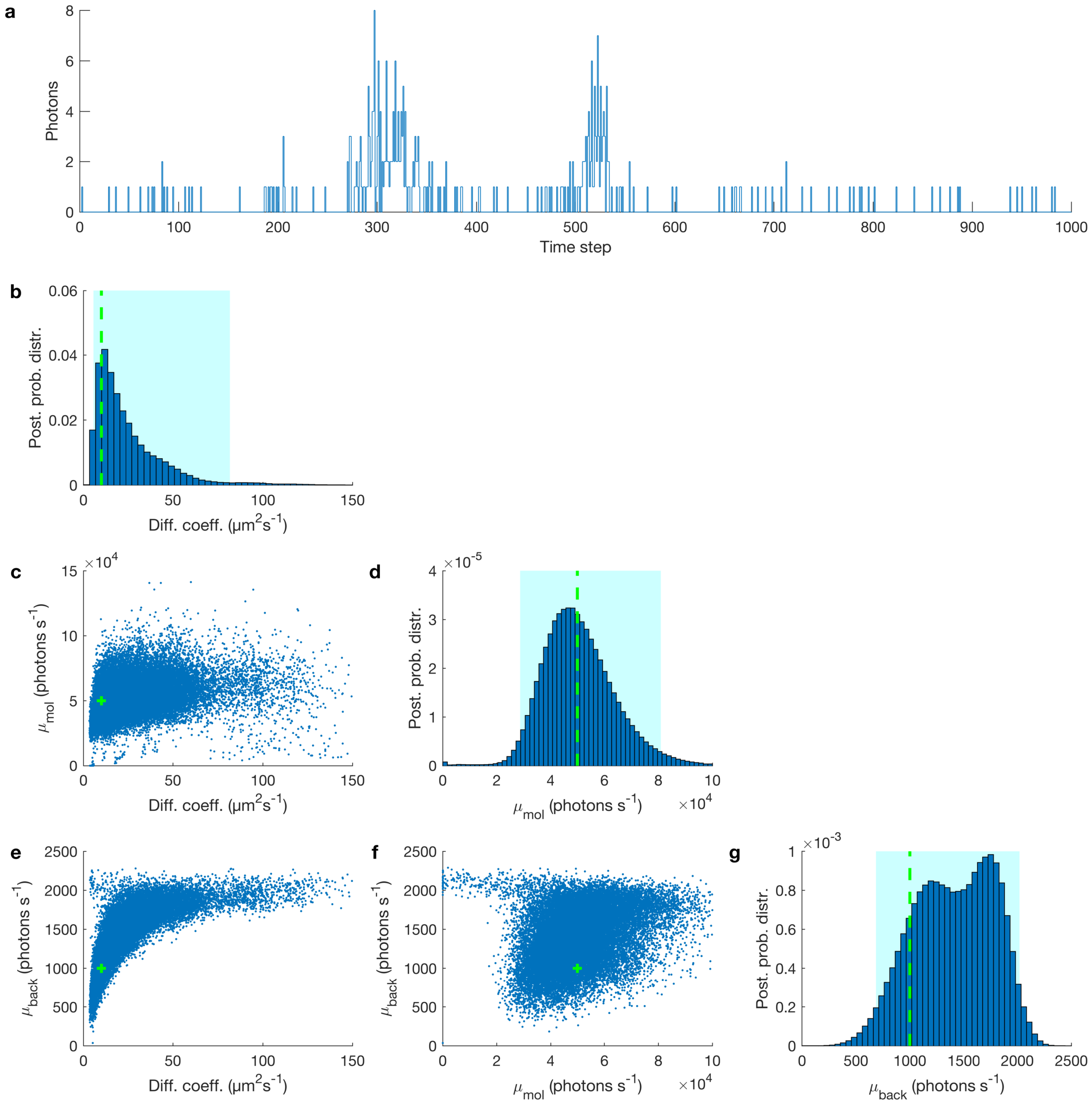

Supplementary Figure 1. **Estimated joint posterior probability distribution. a** Synthetic fluorescent intensity trace used in Fig. 1a with a length of 1000 data points and time step 100 µs. The true values of the diffusion coefficient, molecular brightness and background emission rates are, 10 $\mu m^2 s^{-1}$, 5×10^4 photons s^{-1} and 10^3 photons s^{-1}. **b** The posterior of the diffusion coefficient. **c** The joint probability distribution of the diffusion coefficient and molecular brightness. **d** The posterior probability distribution of the molecular brightness. **e** The joint probability distribution of the diffusion coefficient and molecular brightness. **f** The joint probability distribution of the molecular brightness and background photon emission rates. **g** The posterior probability distribution of the background photon emission rate. The 95% confidence intervals are shown with cyan highlighted regions. The true values are marked throughout by green dashed lines or cross marks.

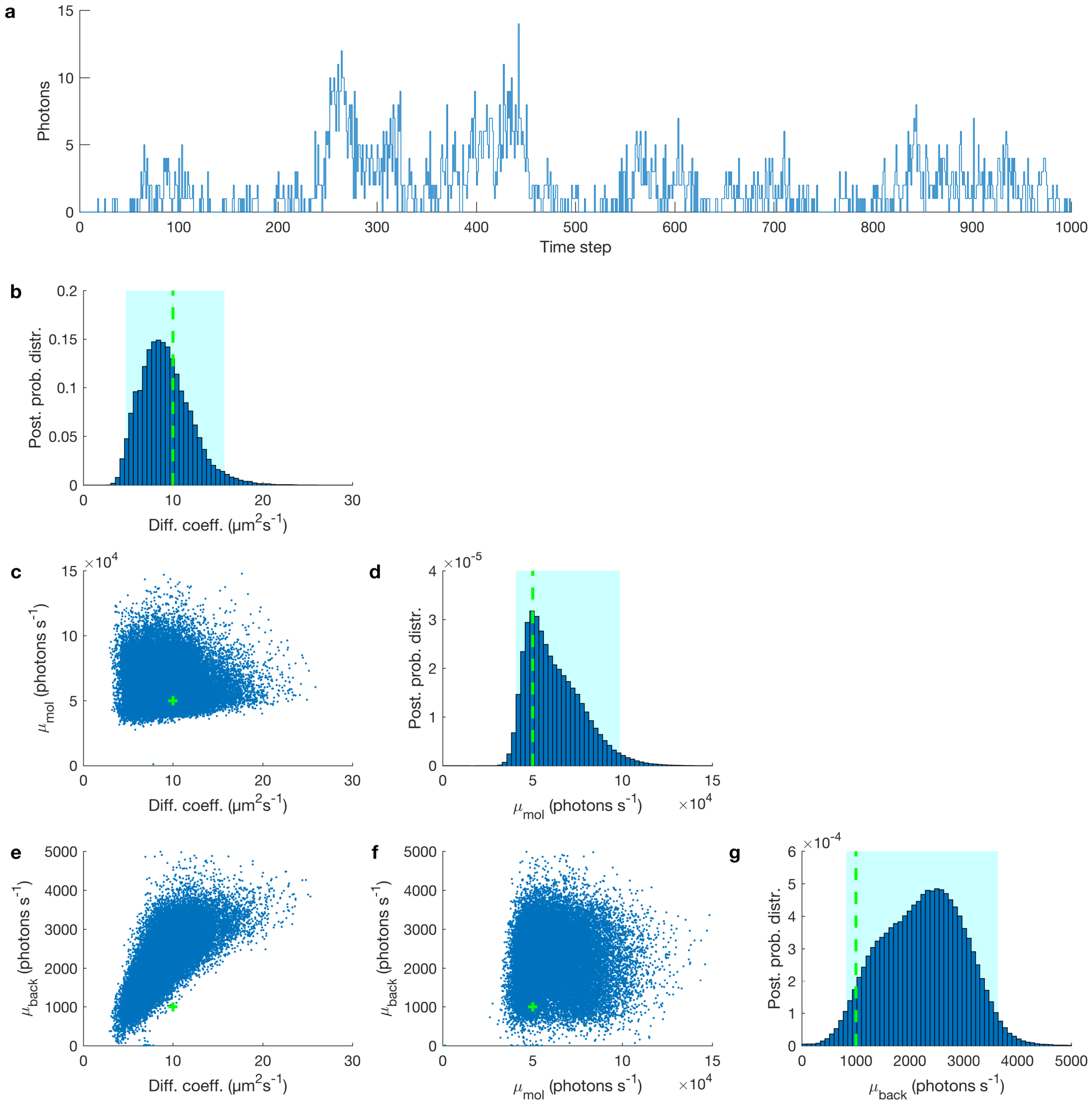

Supplementary Figure 2. **Estimated joint posterior probability distribution.** **a** Synthetic fluorescent intensity trace used in Fig. 1c with a length of 1000 data points and time step 100 μs. The true values of the diffusion coefficient, molecular brightness and background emission rates are, 10 $\mu m^2 s^{-1}$, 5×10^4 photons s^{-1} and 10^3 photons s^{-1}. **b** The posterior of the diffusion coefficient. **c** The joint probability distribution of diffusion coefficient and molecular brightness. **d** The posterior probability distribution of the molecular brightness. **e** The joint probability distribution of the diffusion coefficient and molecular brightness. **f** The joint probability distribution of the molecular brightness and background photon emission rates. **g** The posterior probability distribution of the background photon emission rate. The 95% confidence intervals of the posterior over the number of molecules is highlighted in cyan. The true values are marked throughout by green dashed lines or cross marks.

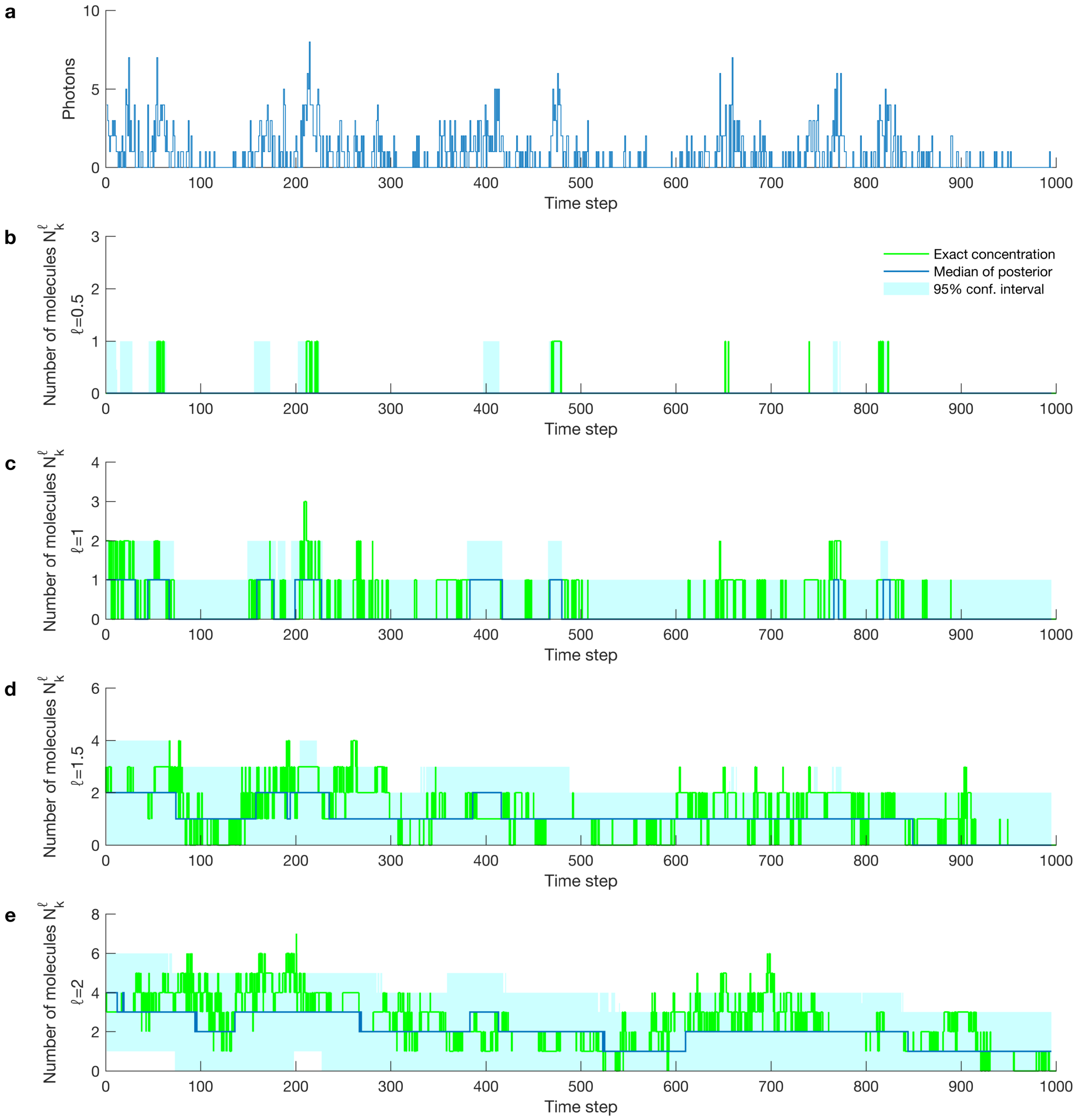

Supplementary Figure 3. **Estimated number of molecules/concentrations.** **a** Synthetic fluorescent intensity trace produced with a molecular brightness of 5×10^4 photons s^{-1} and a background photon emission rate of 10^3 photons s^{-1}, diffusion coefficient of 10 $\mu m^2 s^{-1}$ and 50 molecules. **b–e** Number of molecules estimated from the trace in (**a**) corresponding to normalized distances from the confocal center of $\ell = 0.5, 1, 1.5, 2$. The exact number of molecules is shown by the green lines, the median of the posterior over the number of the molecules is shown by the blue lines, and the 95% confidence intervals of the posteriors over the number of the molecules are highlighted in cyan. For details of the definition of number of molecules N_k^ℓ and the normalized distance ℓ, see Supplementary Equation 23.

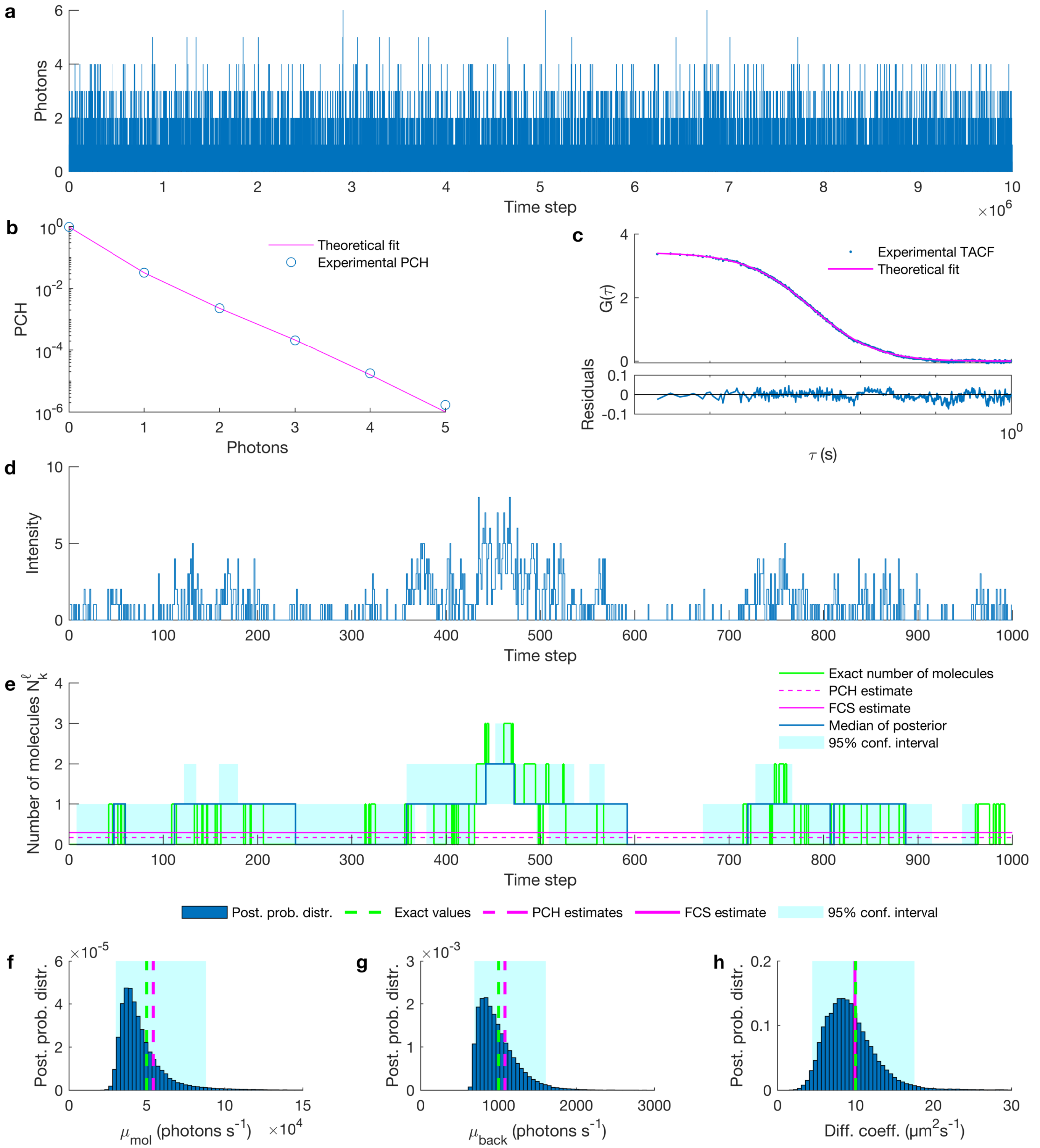

Supplementary Figure 4. Comparison with FCS and PCH. a Targeted synthetic fluorescent intensity trace. The time step is 10 µs and the total duration of the trace is 100 s. **b** PCH curve and the theoretical fit. **c** FCS curve and best theoretical fit. **d** The portion of the trace analyzed by our method rebinned at 100 µs. **e** The number of molecules in the effective volume with ℓ =1, arising from the trace in (**d**). Exact value of the number of molecules is shown by the green line and the PCH and FCS estimates are shown by the dashed and solid pink lines. **g** On the posterior probability distribution of the molecular brightness we superpose the PCH estimate of the molecular brightness (pink dashed line) and the true value (green dashed line). **h** On the posterior probability distribution of the background photon emission rate we superpose the PCH estimate of the background photon emission rate (pink dashed line) and the true value (green dashed line). **f** The posterior probability distribution of the diffusion coefficient obtained by analyzing the trace in (**d**). The FCS estimate of the diffusion coefficient obtained by analyzing the total trace, shown in (**a**), illustrated by a pink dashed line with the exact value (green dashed line). The targeted synthetic trace is generated by freely diffusive molecules with diffusion coefficient, molecular brightness and background photon emission rates of of 10 µm^{2}s^{-1}, 5×10^4 photons s^{-1} and 10^3 photons s^{-1}, respectively. The 95% confidence intervals of the posteriors over the diffusion coefficient, molecular brightness and background photon emission rates are highlighted in cyan.

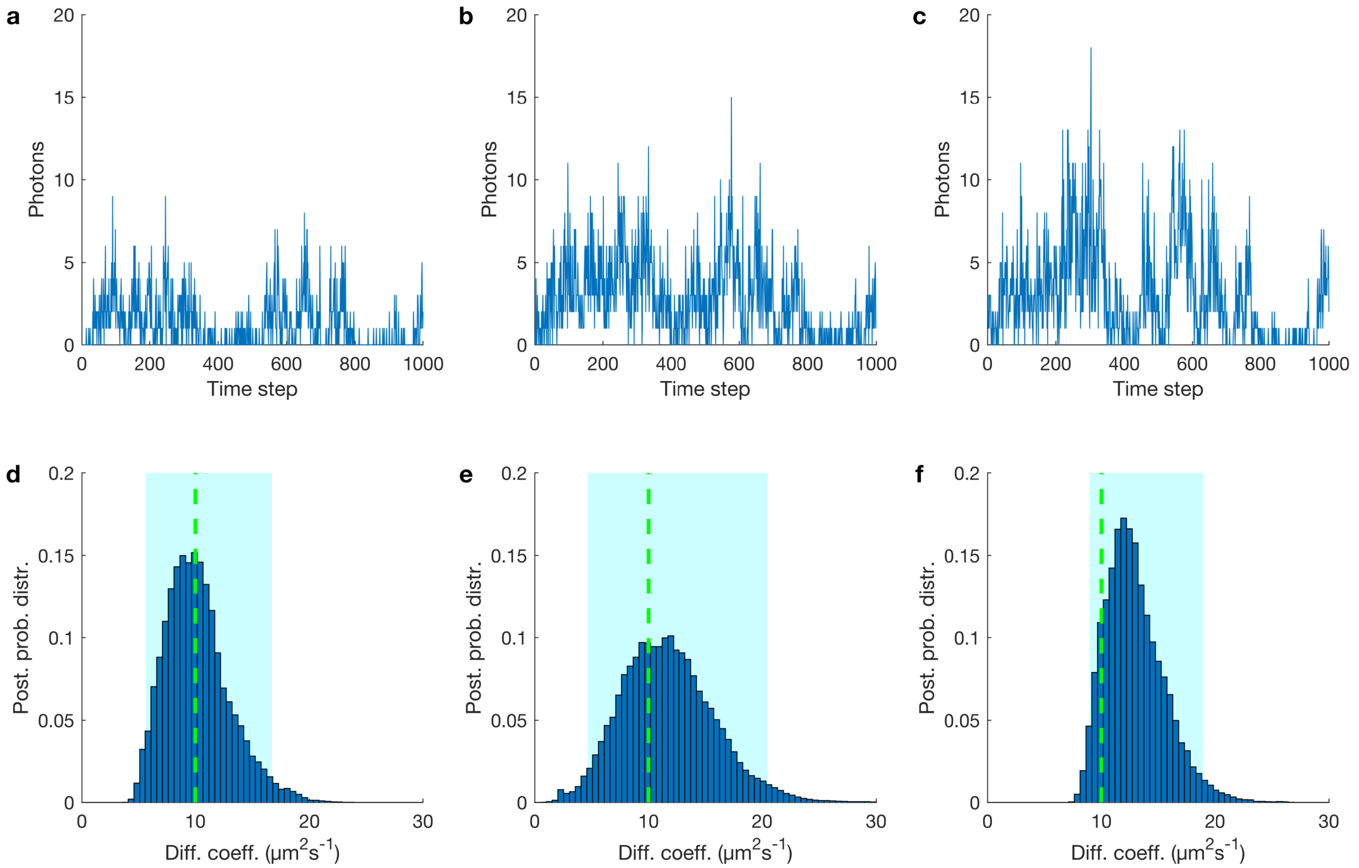

Supplementary Figure 5. **Comparison of the different PSF models**. **a** Synthetic fluorescent intensity trace produced with a 3DG PSF, Supplementary Equation 16. **b** Synthetic fluorescent intensity trace produced with a 2DGL PSF, Supplementary Equation 18. **c** Synthetic fluorescent intensity trace produced with a 2DGC PSF, Supplementary Equation 17. **d** Posterior of the diffusion coefficient using a 3DG PSF model on the trace in (**a**). **e** Posterior of the diffusion coefficient using a 2DGL PSF model on the trace in (**b**). **f** Posterior of the diffusion coefficient using a 2DGC PSF model on the trace in (**c**). To facilitate the comparison, all traces analyzed are generated using the same underlying molecule trajectories with molecular brightness and background photon emission rates set at 5×10^4 photons s^{-1} and 10^3 photons s^{-1} and a diffusion coefficient set at 10 $\mu\mathrm{m}^2\mathrm{s}^{-1}$ (shown by green dashed lines).

2. Analysis of additional experimental data

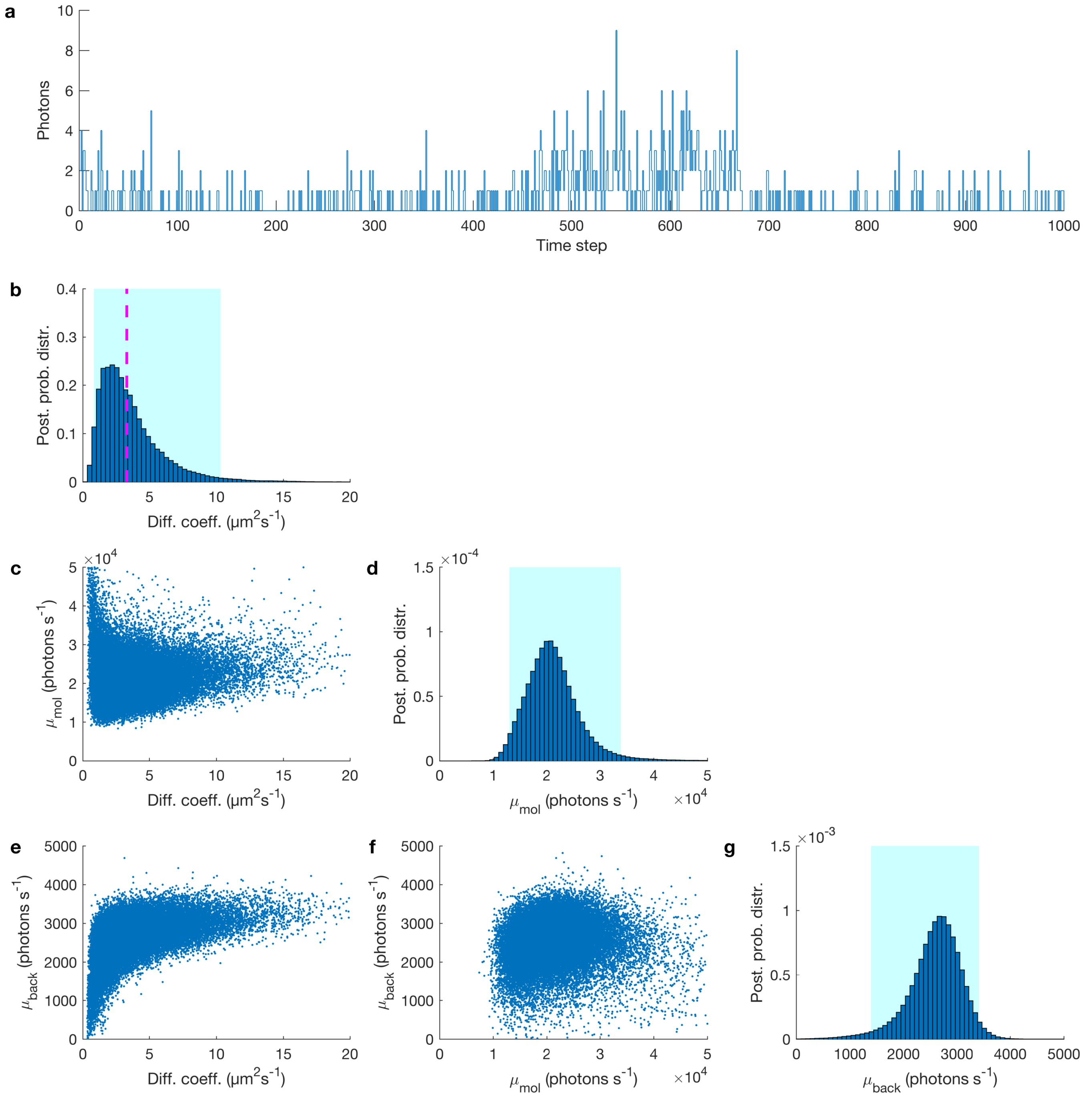

Supplementary Figure 6. **Estimated joint posterior probability distribution.** **a** Experimental fluorescent intensity trace used in Fig. 5e with a length of 1000 data points and time step 100 μs. **b** The posterior of the diffusion coefficient. The FCS estimate is shown by a magenta dashed line. **c** The joint probability distribution of the diffusion coefficient and the molecular brightness. **d** The posterior probability distribution of the molecular brightness. **e** The joint probability distribution of the diffusion coefficient and molecular brightness. **f** The joint probability distribution of the molecular brightness and background photon emission rates. **g** The posterior probability distribution of the background photon emission rate and the 95% confidence intervals of the posteriors are highlighted in cyan. The experimental fluorescent intensity trace was produced with a concentration of 100 pM of Cy3 in a 94% glycerol/water mixture.

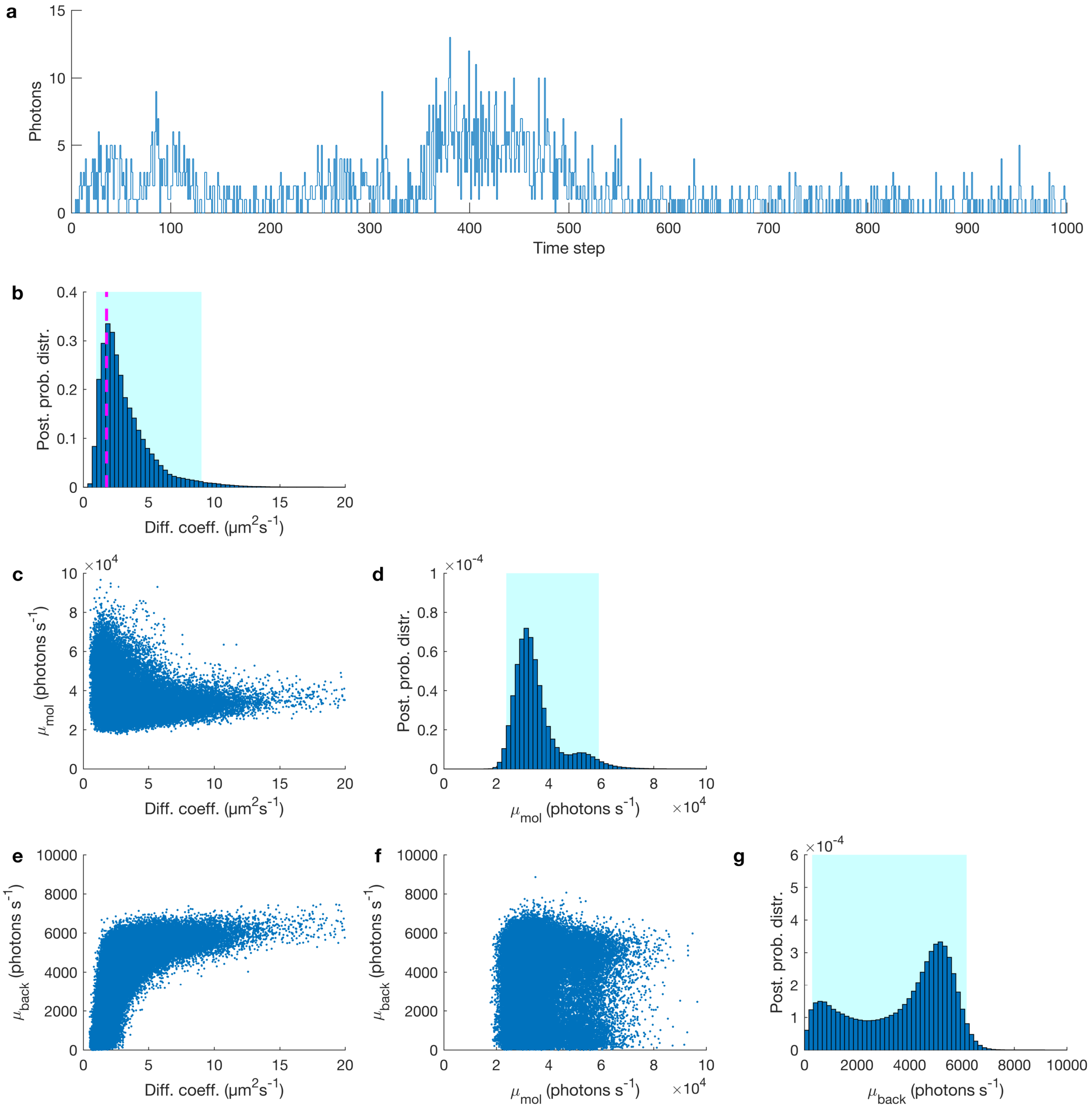

Supplementary Figure 7. **Estimated joint posterior probability distribution.** **a** Experimental fluorescent intensity trace used in Fig. 5f with a length of 1000 data points and time step 100 μs. **b** The posterior of the diffusion coefficient. The FCS estimate is shown by a magenta dashed line. **c** The joint probability distribution of the diffusion coefficient and the molecular brightness. **d** The posterior probability distribution of the molecular brightness. **e** The joint probability distribution of the diffusion coefficient and the molecular brightness. **f** The joint probability distribution of the molecular brightness and background photon emission rates. **g** The posterior probability distribution of the background photon emission rate and the 95% confidence intervals of the posteriors are highlighted in cyan. The experimental fluorescent intensity trace produced is with a concentration of 1 nM of Cy3 in a 94% glycerol/water mixture.

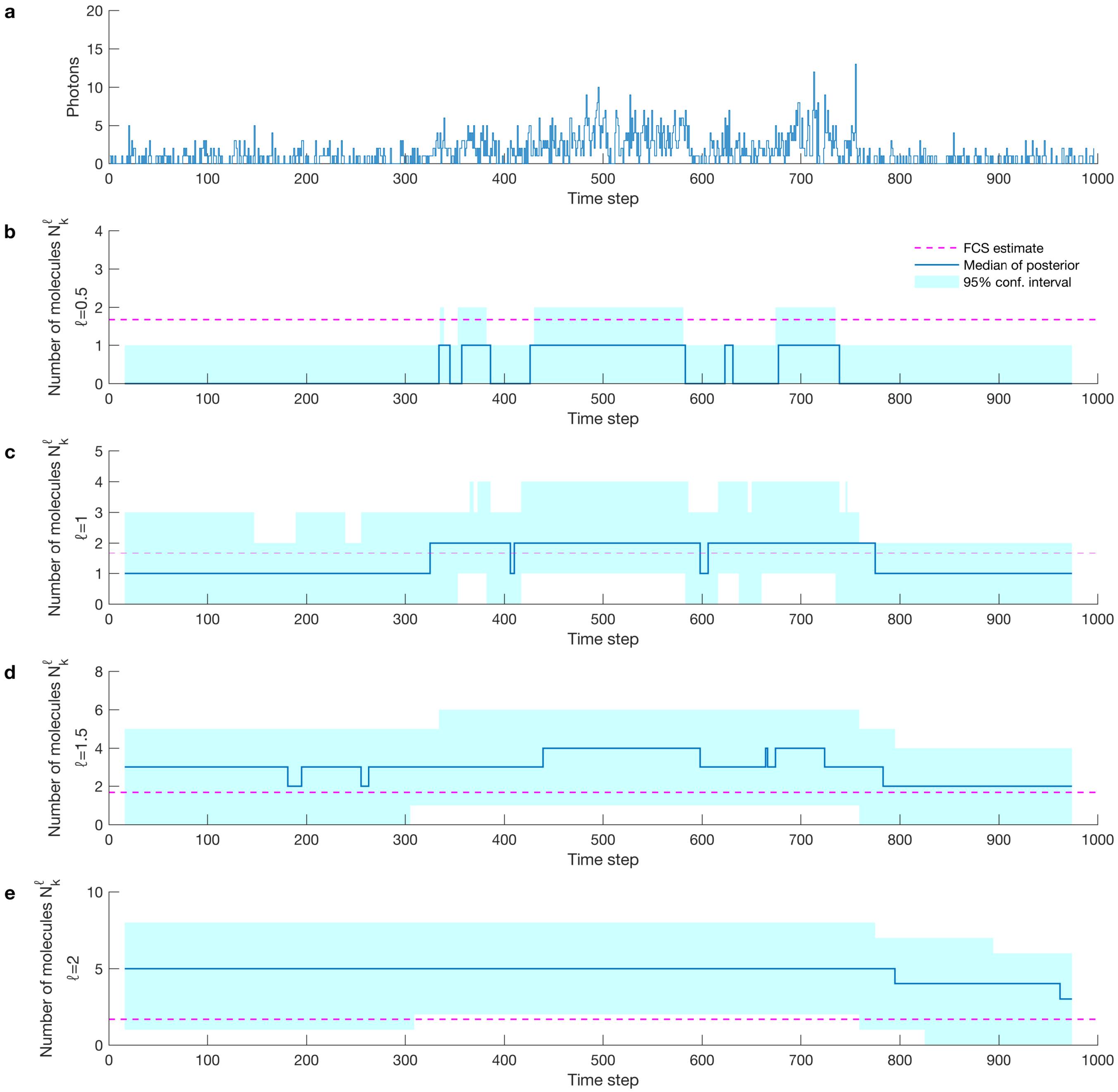

Supplementary Figure 8. **Estimated number of molecules/concentrations**. **(a)** Experimental fluorescent intensity trace produced with a concentration of 1 nM of Cy3 in a 94% glycerol/water mixture. **b–e** Number of molecules estimated from the trace in (**a**) with $\ell = 0.5, 1, 1.5, 2$, respectively. The FCS estimate of the average number of molecules in the effective volume ($\sim$1.68 molecules) by analyzing a 3 minutes long time trace, is shown by the magenta dashed lines and the median of the posterior over the number of the molecules is shown by a blue line. The 95% confidence interval of the posterior over the number of the molecules is highlighted in cyan. For details of the definition of number of the molecules N_k^ℓ and the normalized distance ℓ, see Supplementary Equation 23, below.

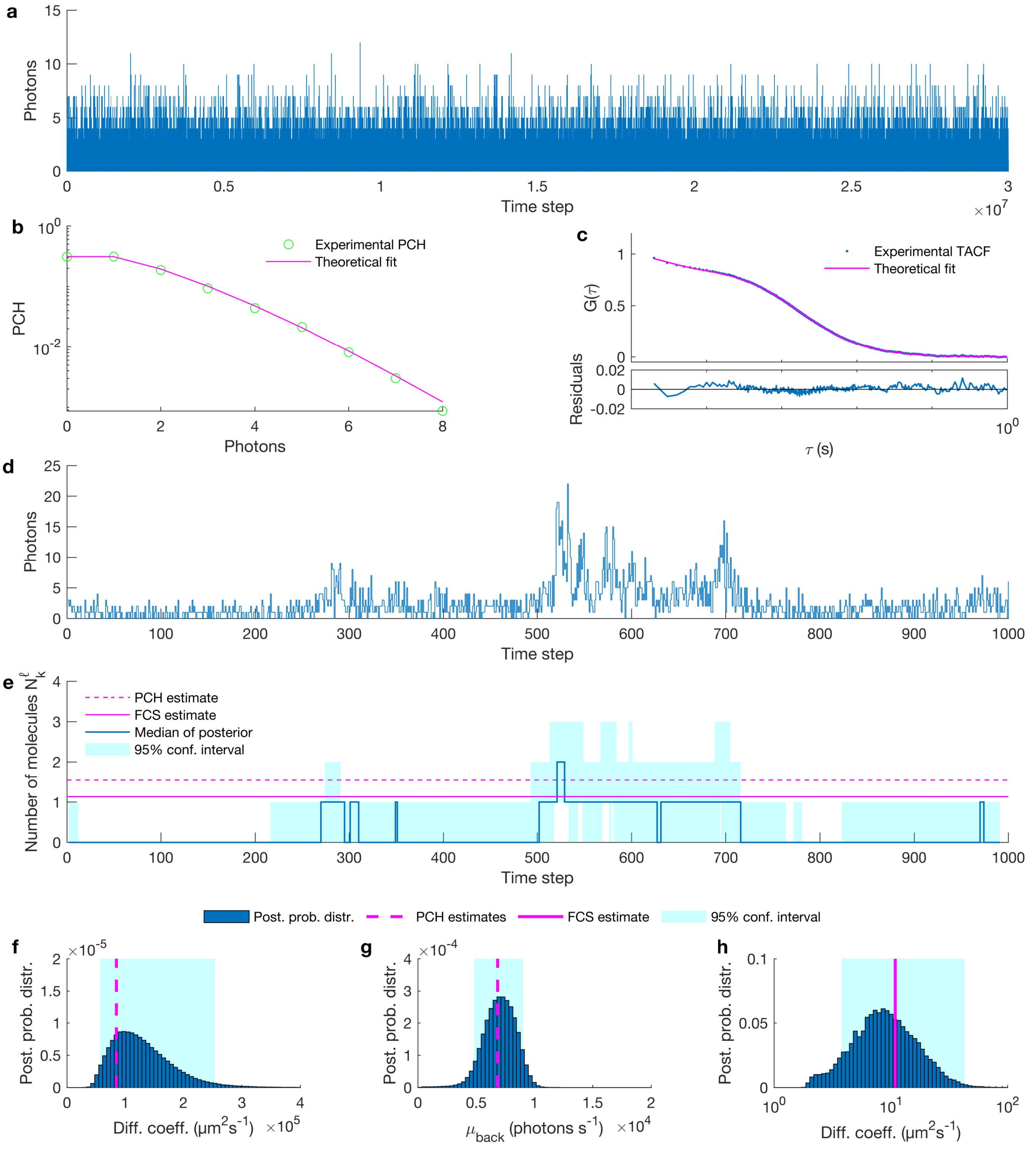

Supplementary Figure 9. **Comparison with FCS and PCH. a** Targeted experimental fluorescent intensity trace. The time step is 10 μs with a total time of 5 min. **b** PCH curve and the theoretical fit. **c** FCS curve and the best theoretical fit. **d** The portion of the trace analyzed by our method rebinned at 100 μs. **e** The concentration of Cy3 in the effective volume with $\ell = 1$, arising from the trace in (**d**). The experimental concentration is shown by the green line and the PCH estimated is shown by the pink line. **g** The posterior probability distribution of the molecular brightness with the PCH estimated of the molecular brightness shown by a solid green line. **h** The posterior probability distribution of the background photon emission rate with the PCH estimate of the background photon emission rate shown by a solid green line. **f** The posterior probability distribution of the diffusion coefficient obtained by analyzing the trace in (**d**). The FCS estimate of the diffusion coefficient obtained by analyzing the total time trace, shown in (**a**), is denoted by a pink solid line. The targeted experimental trace is generated by free diffusive Cy3 in a mixture of water and glycerol with 75% glycerol, a laser power of 100 μW and a concentration of Cy3 at 1 nM, excitation wavelength, NA and refractive index used are 532 nm, 1.42 and 1.4, respectively.

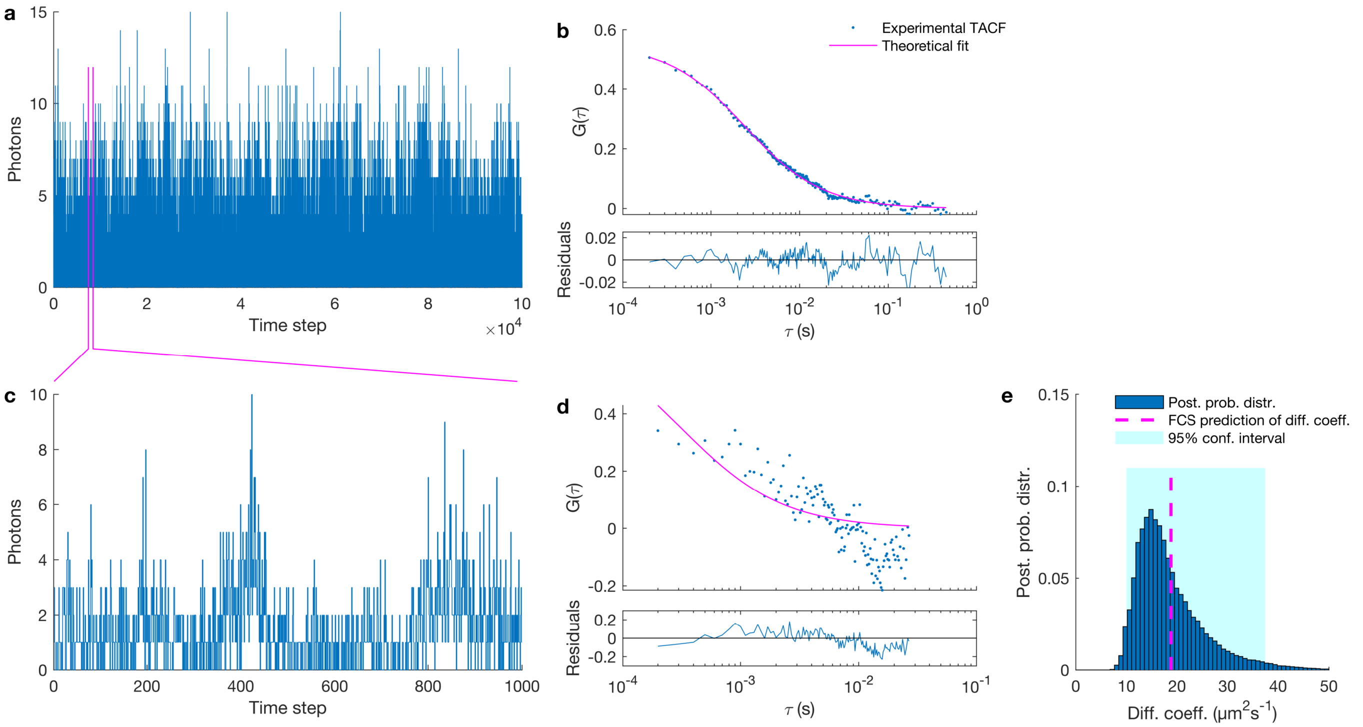

Supplementary Figure 10. **Experimental traces of free Cy3B dyes using an elongated confocal volume.** **a** Experimental fluorescent intensity trace used in FCS. The time trace is generated by 2.5 nM Cy3B dyes in glycerol/water mixture with 70% glycerol and laser power of 100 μW. **b** Auto-correlation curve of the trace in (**a**) and best theoretical fit. **c** Portion of the trace in (a) to be used as the input to FCS and our method. **d** Auto-correlation curve of trace in (**c**). **e** Posterior probability distribution over the diffusion coefficient estimated from the trace in (**c**). Traces shown in (**a**) and (**c**) are acquired at 100 μs for a total of 10 s and 0.1 s respectively. The laser power use to generate the signal (**a**) is 100 μW (measured before the beam enters the objective). The estimation of the diffusion coefficient as the results of autocorrelation fitting in (**a**) matched with Stokes-Einstein prediction, equal to 18.79 $\mu m^2 s^{-1}$ and in (**d**) is 145.75 $\mu m^2 s^{-1}$.

3. Saturation test

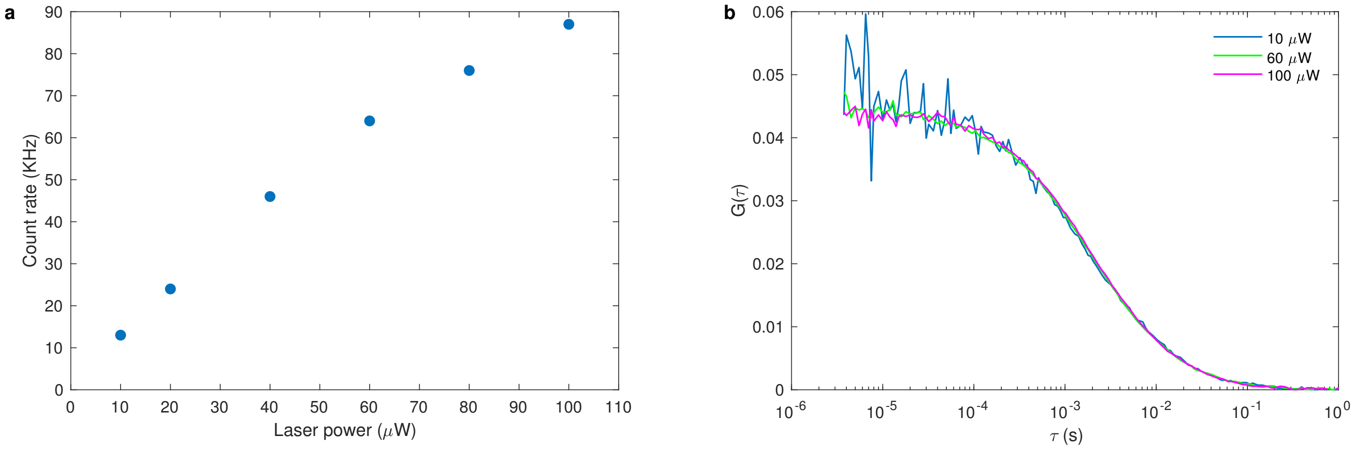

Supplementary Figure 11. **Testing the experimental setup for effects of saturation.** **a** Fluorescence count rate shown here is plotted with respect to the laser intensity. Laser powers are, conservatively, measured at the output of the laser. As such, we expect these to be lower at the entrance of the objective. **b** Auto-correlation curves of the time traces used in (**a**). Only three of the six acquired FCS decays are shown for clarity (they all overlap within experimental error). Fluorescent intensity time traces were obtained using a solution of Cy3B dyes in glycerol/water mixture with 70% glycerol at six different laser powers in the 10-100 μW range. Despite a slight nonlinearity in (**a**), there is no effect of saturation within the experimental error in (**b**).

4. Analysis of additional data for multiple diffusive species

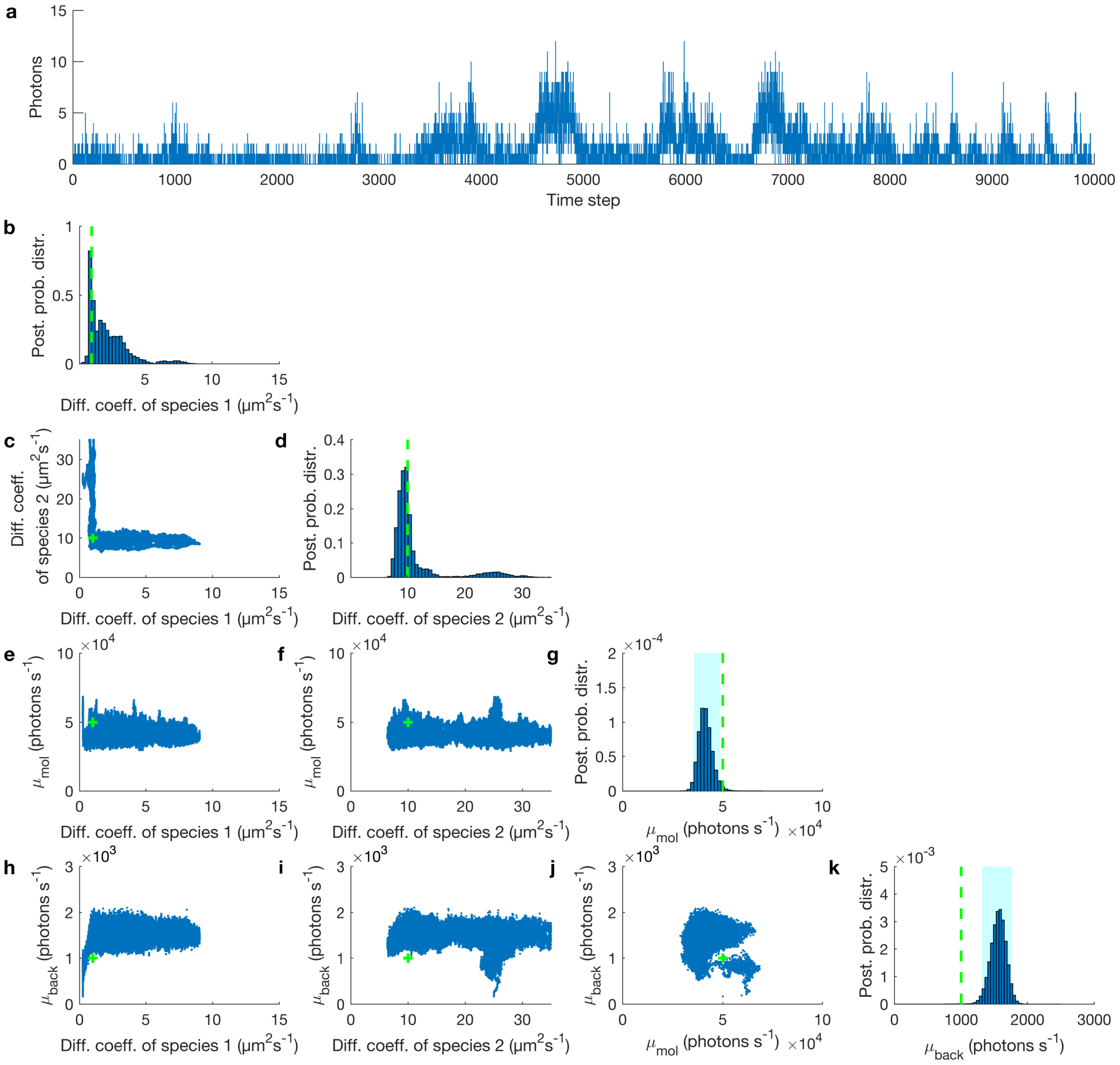

Supplementary Figure 12. **Joint posterior probability distribution of multiple diffusive species**. **a** A mixed fluorescent intensity trace was obtained by combining the traces from two different synthetic signals with molecular brightness and background emission rates of 5×10^4 and 10^3 photons/s, respectively, and diffusion coefficients of 1 and 10 μm^{2}s^{-1}. **b** The posterior probability distribution of the diffusion coefficient of diffusive species 1. **c** The joint probability distribution of the diffusion coefficient for diffusive species 1 and diffusive species 2. **d** The posterior probability distribution of the diffusion coefficient of diffusive species 2. **e** The joint probability distribution of diffusion coefficient of diffusive species 1 along with the molecular brightness. **f** The joint probability distribution of diffusion coefficient of diffusive species 2 along with the molecular brightness. **g** The posterior probability distribution of the molecular brightness. **h** The joint probability distribution of diffusion coefficient for diffusive species 1 along with the background photon emission rate. **i** The joint probability distribution of the diffusion coefficient of diffusive species 2 and the background photon emission rate. **j** The joint probability distribution of the molecular brightness and background photon emission rate. **k** The posterior probability distribution of the background photon emission rate. The trace is binned at 100 μs with a total trace duration of 1 s. The exact values of the parameters are shown by green dashed lines and the 95% confidence intervals of the posteriors are highlighted in cyan.

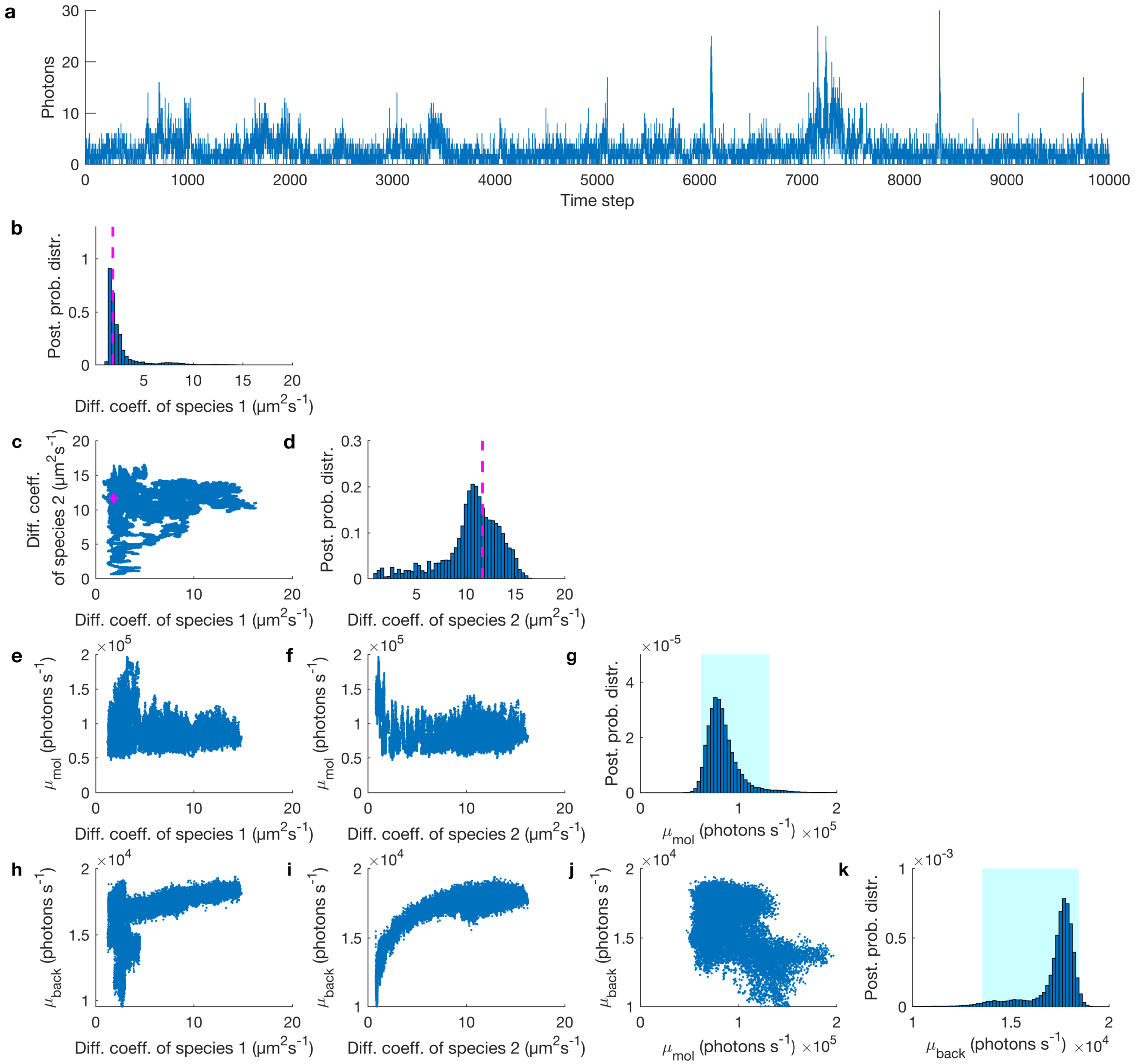

Supplementary Figure 13. **Joint posterior probability distribution of multiple diffusive species.** **a** Experimental fluorescent intensity trace used in Fig. 7c with length 10^4 data points and step 100μs. **b** The posterior probability distribution of the diffusion coefficient of diffusive species 1. **c** The joint probability distribution of diffusion coefficient of diffusive species 1 and diffusive species 2. **d** The posterior probability distribution of the diffusion coefficient of diffusive species 2. **e** The joint probability distribution of diffusion coefficient of diffusive species 1 along with the molecular brightness. **f** The joint probability distribution of diffusion coefficient of diffusive species 2 along with the molecule photon emission rate. **g** The posterior probability distribution of the molecular brightness. **h** The joint probability distribution of diffusion coefficient of diffusive species 1 and background photon emission rate. **i** The joint probability distribution of diffusion coefficient of diffusive species 2 and background photon emission rate. **j** The joint probability distribution of the molecular brightness and background photon emission rate. **k** The posterior probability distribution of the background photon emission rate. The trace is generated by mixing two experimental traces of concentration 1 nM of freely diffusive Cy3 in a water/glycerol mixtures with 94% and 75% glycerol each. The laser power, wavelength, NA and refractive index are 100 μW, 532 nm, 1.42 and 1.4, respectively. The FCS estimates are shown by a magenta dashed lines and the 95% confidence intervals of the posteriors are highlighted in cyan.

Supplementary Note 2: Summary of point estimates

Supplementary Table 1. Summary of the posterior probability distributions as point estimates

	D		μ_{mol}		μ_{back}	
	mean	std	mean	std	mean	std
	$\mu m^2 s^{-1}$	$\mu m^2 s^{-1}$	photons s^{-1}	photons s^{-1}	photons s^{-1}	photons s^{-1}
Fig. 1(a)	25.07	20.61	5.06×10^4	1.35×10^4	1.52×10^3	0.40×10^3
Fig. 1(c)	9.21	2.82	5.96×10^4	1.49×10^4	2.32×10^3	0.79×10^3
Fig. 2(a)	1.07×10^{-2}	0.48×10^{-2}	4.69×10^4	1.31×10^4	1.11×10^3	0.79×10^3
	1.23×10^{-1}	0.64×10^{-1}	4.11×10^4	2.01×10^4	1.46×10^3	1.13×10^3
	1.41	0.67	6.53×10^4	1.50×10^4	3.85×10^3	0.96×10^3
	9.27	2.82	5.96×10^4	1.35×10^4	5.63×10^3	1.48×10^3
	180.45	173.25	6.11×10^4	5.18×10^4	3.87×10^3	0.31×10^3
Fig. 2(b)	8.75	7.04	3.64×10^4	8.14×10^3	4.64×10^3	2.17×10^3
	10.22	2.98	3.55×10^4	5.54×10^4	4.16×10^3	1.13×10^3
	9.73	1.97	5.64×10^4	8.36×10^3	6.12×10^3	0.56×10^3
	10.53	1.18	5.08×10^4	4.53×10^3	4.87×10^3	0.37×10^3
	10.05	0.42	4.89×10^4	2.13×10^3	1.35×10^3	0.23×10^3
Fig. 3(a)	10.65	1.68	9.85×10^4	1.36×10^4	2.17×10^3	0.91×10^3
Fig. 3(b)	10.04	2.38	5.48×10^4	6.47×10^3	2.09×10^3	0.53×10^3
Fig. 3(c)	11.29	4.14	1.02×10^4	6.97×10^3	2.66×10^3	0.19×10^3
Fig. 4(c)	10.17	3.09	1.46×10^4	1.42×10^3	2.87×10^3	0.43×10^3
Fig. 5(a)	0.55	0.16	2.18×10^4	1.44×10^4	1.79×10^3	2.21×10^3
	2.28	1.03	3.51×10^4	9.21×10^3	3.55×10^3	1.85×10^3
	12.80	4.51	1.30×10^5	5.17×10^4	1.00×10^4	1.96×10^3
	27.96	10.30	9.15×10^4	2.37×10^4	7.01×10^3	0.46×10^3
Fig. 5(b)	7.62	106.51	5.15×10^4	5.14×10^4	1.08×10^3	3.98×10^3
	2.28	1.03	3.51×10^4	9.21×10^3	3.55×10^3	1.85×10^3
	2.04	0.33	3.21×10^4	8.05×10^3	8.25×10^3	0.27×10^3
	1.90	0.06	3.14×10^4	6.54×10^3	6.23×10^3	0.48×10^3
	1.78	0.02	3.07×10^4	4.84×10^3	5.68×10^3	0.69×10^3
Fig. 5(c)	7.50	6.82	2.20×10^4	9.55×10^3	1.06×10^3	0.22×10^3
Fig. 5(d)	3.68	2.73	4.32×10^4	2.13×10^4	2.59×10^3	0.52×10^3
Fig. 5(e)	2.70	2.68	2.31×10^4	5.47×10^3	2.94×10^3	2.14×10^3
Fig. 5(f)	2.28	1.03	3.51×10^4	9.21×10^3	3.55×10^3	1.85×10^3
Fig. 6(a)	0.36	0.22	7.19×10^3	2.31×10^3	1.42×10^3	1.98×10^3
	4.48	1.35	2.75×10^4	3.03×10^3	0.98×10^3	0.94×10^3
	10.66	4.06	6.61×10^4	1.33×10^4	2.33×10^3	1.51×10^3
	102.26	27.74	2.36×10^5	5.70×10^4	9.33×10^3	0.77×10^3
Fig. 6(b)	10.86	4.91	3.42×10^4	2.35×10^4	2.59×10^3	0.59×10^3
Fig. 6(c)	10.54	4.22	5.03×10^4	2.03×10^4	3.78×10^3	0.62×10^3
Fig. 6(d)	11.76	6.42	3.12×10^4	6.09×10^3	8.41×10^3	1.21×10^3
Fig. 6(e)	10.66	4.06	6.61×10^4	1.33×10^4	2.33×10^3	1.51×10^3
Fig. 7(a)	1.98	0.90	2.09×10^5	2.10×10^4	1.06×10^4	1.64×10^3
Fig. 7(b)	12.65	3.22	4.22×10^4	1.54×10^4	2.57×10^3	0.49×10^3
Fig. 7(c)	-	-	6.79×10^4	4.58×10^3	1.46×10^4	0.71×10^3
Supplementary Figure 4	9.44	3.97	4.62×10^4	1.59×10^4	0.98×10^3	0.25×10^3
Supplementary Figure 5(a)	10.16	2.81	2.71×10^4	3.63×10^3	2.26×10^3	0.62×10^3
Supplementary Figure 5(b)	11.71	4.04	4.89×10^4	1.06×10^4	6.78×10^3	1.79×10^3
Supplementary Figure 5(c)	12.93	2.95	6.05×10^4	1.31×10^4	3.34×10^3	0.98×10^3
Supplementary Figure 9	15.73	10.43	1.29×10^5	5.17×10^4	7.21×10^3	1.15×10^3
Supplementary Figure 10(c)	18.72	7.18	2.63×10^4	5.93×10^3	7.26×10^3	6.46×10^3
Supplementary Figure 12	-	-	4.14×10^4	3.38×10^3	1.55×10^3	0.13×10^3

Here we list characteristic values (point estimates) summarizing the posterior probability distributions of this study. Mean and std refer to posterior mean value and standard deviation (i.e. square root of variance). Values are listed according to figures.

Supplementary Note 3: Detailed methods description

1. Representation of molecular diffusive motion

Consider a particle moving in 1D diffusion. The probability distribution $p(x,t)$ of the particle's location obeys Fick's second law [1–3] and is given by the diffusion equation

$$\frac{\partial p}{\partial t} = D\frac{\partial^2 p}{\partial x^2} \qquad \text{Supplementary Equation 1}$$

where D is the particle's diffusion coefficient. Assuming the particle is located at x_{k-1} at a time t_{k-1}, i.e. assuming the initial condition $p(x, t_{k-1}) = \delta(x - x_{k-1})$, and a free space boundary, i.e. $\lim_{x\to\pm\infty} p(x,t) = 0$, we can solve this equation to obtain $p(x,t)$ for any later time t. The solution is

$$p(x,t) = \frac{\exp\left(-\frac{(x-x_{k-1})^2}{4(t-t_{k-1})D}\right)}{\sqrt{4\pi(t-t_{k-1})D}} \qquad \text{Supplementary Equation 2}$$

which equals to the probability density of a normal random variable with mean x_{k-1} and variance $2(t - t_{k-1})D$, see Supplementary Table 4. At time $t = t_k$, we therefore have

$$x_k|x_{k-1} \sim \mathbf{Normal}\left(x_{k-1}, 2(t_k - t_{k-1})D\right). \qquad \text{Supplementary Equation 3}$$

Similarly, solving the diffusion equation for particles following isotropic 3D diffusion in free space, we have

$$\begin{aligned} x_k|x_{k-1} &\sim \mathbf{Normal}\left(x_{k-1}, 2(t_k - t_{k-1})D\right) \\ y_k|y_{k-1} &\sim \mathbf{Normal}\left(y_{k-1}, 2(t_k - t_{k-1})D\right) \\ z_k|z_{k-1} &\sim \mathbf{Normal}\left(z_{k-1}, 2(t_k - t_{k-1})D\right) \end{aligned} \qquad \text{Supplementary Equation 4}$$

which constitute the molecular motion model used throughout this study.

2. Description of Stokes-Einstein model

For the experimental data, we benchmark our estimates of the diffusion coefficient against the Stokes-Einstein prediction [2, 3]. Namely, for a spherical particle in a quiescent fluid at uniform temperature

$$D = \frac{kT}{6\pi r\eta} \qquad \text{Supplementary Equation 5}$$

where, D is the diffusion coefficient, k is Boltzmann's constant, T is the solution's absolute temperature, r is the hydrodynamic radius of the particle and η is the solution's dynamic viscosity [4].

3. FCS formulation

The formulation we used in this study to autocorrelate the synthetic and experimental time traces is

$$G_{ex}(\tau) = \frac{\langle I(t+\tau)I(t)\rangle}{\langle I(t)\rangle^2} - 1 \qquad \text{Supplementary Equation 6}$$

where the $I(t)$ is the number of detected photons at time t. The computational implementation uses the Wiener-Khinchin theorem [5].

The theoretical function [6–9] used to fit the autocorrelation curves, for a 3DG PSF, is

$$G_{th}(\tau) = \frac{1}{\langle N\rangle}\frac{1 - F + Fe^{-\frac{\tau}{\tau_F}}}{(1-F)}\frac{1}{1+\frac{4D\tau}{\omega_{\mathrm{xy}}^2}}\frac{1}{(1+\frac{4D\tau}{\omega_{\mathrm{z}}^2})^{\frac{1}{2}}} \qquad \text{Supplementary Equation 7}$$

and, for the 2DGL PSFs, is

$$G_{th}(\tau) = \frac{1}{\langle N \rangle} \frac{1 - F + Fe^{-\frac{\tau}{\tau_F}}}{(1-F)} \frac{1}{1 + \frac{4D\tau}{\omega_{\mathrm{xy}}^2}} + 1 \qquad \text{Supplementary Equation 8}$$

where $\langle N \rangle$ is the average number of molecule in the effective volume, D is the diffusion coefficient, τ_F is the triplet state relaxation time and F is the fraction molecules populating the triplet state.

To find the best fit, we use χ^2 minimization [10–12]

$$\chi^2 = \sum_{\tau} (G_{th}(\tau) - G_{ex}(\tau))^2. \qquad \text{Supplementary Equation 9}$$

4. Definition of molecular brightness

As the definition of molecular brightness in Eq. 2, we use the emission rate of detected photons of a single fluorophore. For a fluorophore located at (x, y, z) this is formulated as

$$\mu(x,y,z) = \mu_0\ \varphi_{\mathrm{d}}\ \varphi_{\mathrm{qe}}\ \varphi_{\mathrm{f}}\ \sigma\ \mathrm{EXC}(x,y,z)\ \mathrm{CEF}(x,y,z) \qquad \text{Supplementary Equation 10}$$

where, μ_0 is the maximum excitation intensity which occurs at the center of the confocal volume, φ_{d} is the efficiency of the photon collection at the center of the confocal volume, φ_{qe} is the quantum efficiency of the detector, φ_{f} is the quantum efficiency of the fluorophore (i.e. quantum yield), σ is the absorption cross-section of the fluorophore, $\mathrm{EXC}(x, y, z)$ is the excitation profile and $\mathrm{CEF}(x, y, z)$ is the detection profile, i.e. collection efficiency function, which equals the fraction of the photons collected by the detector to the total photons emitted by a point source [13].

To obtain Eq. 2, we cast Supplementary Equation Supplementary Equation 10 in the simplified form

$$\mu(x,y,z) = \mu_{\mathrm{mol}} \mathrm{PSF}(x,y,z) \qquad \text{Supplementary Equation 11}$$

where $\mu_{\mathrm{mol}} = \mu_0\, \varphi_{\mathrm{d}}\, \varphi_{\mathrm{qe}}\, \varphi_{\mathrm{f}}\, \sigma$, which we term molecular brightness at the center of the confocal volume [14], and $\mathrm{PSF}(x, y, z) = \mathrm{EXC}(x, y, z)\, \mathrm{CEF}(x, y, z)$, which we term the PSF.

To relate the parameter μ_{mol} to the average photon count rate, which is commonly estimated in bulk experiments [15–17], we consider the spatial average of $\mu(x, y, z)$ as follows

$$\langle \mu(x,y,z) \rangle = \mu_{\mathrm{mol}} \langle \mathrm{PSF}(x,y,z) \rangle. \qquad \text{Supplementary Equation 12}$$

For the specific choice of a 3DG PSF (see below), the average is computed as follows

$$\langle \mathrm{PSF}(x,y,z) \rangle = \frac{\int_{-\infty}^{+\infty} \int_{-\infty}^{+\infty} \int_{-\infty}^{+\infty} \exp\left(-2\frac{x^2}{\omega_{\mathrm{xy}}^2} - 2\frac{y^2}{\omega_{\mathrm{xy}}^2} - 2\frac{z^2}{\omega_{\mathrm{z}}^2}\right) dxdydz}{V_{\mathrm{eff}}} = \sqrt{\frac{\pi}{2}\omega_{\mathrm{xy}}^2} \sqrt{\frac{\pi}{2}\omega_{\mathrm{xy}}^2} \sqrt{\frac{\pi}{2}\omega_{\mathrm{z}}^2} \frac{1}{V_{\mathrm{eff}}} \qquad \text{Supplementary Equation 13}$$

where V_{eff} denotes the effective volume of 3DG PSF [8, 18] and it is given by

$$V_{\mathrm{eff}} = \pi^{\frac{3}{2}} \omega_{\mathrm{xy}}^2 \omega_{\mathrm{z}}. \qquad \text{Supplementary Equation 14}$$

Consequently, Supplementary Equation Supplementary Equation 13 implies

$$\mu_{\mathrm{mol}} = \sqrt{8}\, \langle \mu(x,y,z) \rangle. \qquad \text{Supplementary Equation 15}$$

In other words, the molecular brightness is, by definition, approximately 2.8 times larger than the average photon count rate of a single molecule [15–17].

5. Definition of point spread function models

In this study we use three different point spread functions as approximations to the more realistic Airy function [19–21], namely a 3D-Gaussian (3DG) [22], a 2D-Gaussian-Cylindrical (2DGC) [22] and a 2D-Gaussian-Lorentzian (2DGL) [23–26].

The definition of the PSF for the 3DG case is

$$\text{PSF}_{\text{3DG}}(x,y,z) = \exp\left(-2\frac{x^2+y^2}{\omega_{\text{xy}}^2} - 2\frac{z^2}{\omega_{\text{z}}^2}\right) \qquad \text{Supplementary Equation 16}$$

while, the definition of the PSF for the 2DGC case is

$$\text{PSF}_{\text{2DGC}}\,(x,y,z) = \exp\left(-2\frac{x^2}{\omega_{\text{xy}}^2} - 2\frac{y^2}{\omega_{\text{xy}}^2}\right). \qquad \text{Supplementary Equation 17}$$

For both cases, ω_{xy} and ω_z are the semi-axes lateral and parallel to the optical axis. These are represented in terms of the excitation wavelength λ_{exc}, solution refraction index n_{sol}, and numerical aperture NA of the microscope as $\omega_{\text{xy}} = 0.61\lambda_{\text{exc}}/\text{NA}$ and $\omega_z = 1.5n_{\text{sol}}\lambda_{\text{exc}}/\text{NA}^2$; for example see [27, 28]. For more realistic representations, ω_{xy} and ω_{z} can be estimated directly based on calibration experiments with known diffusion coefficients; for example see [29].

The definition of the PSF for the 2DGL case is

$$\text{PSF}_{\text{2DGL}}\,(x,y,z) = \frac{1}{1+\left(\frac{z}{z_{\text{R}}}\right)^2}\exp\left(\frac{-2\frac{x^2+y^2}{\omega_{\text{xy}}^2}}{1+\left(\frac{z}{z_R}\right)^2}\right) \qquad \text{Supplementary Equation 18}$$

where ω_{xy}, λ_{exc}, and n_{sol} are similar to the 3DG or 2DG cases and $z_{\text{R}} = n_{\text{sol}}\pi\omega_{\text{xy}}^2/\lambda_{\text{exc}}$.

6. Description of the data simulation

To generate fluorescence intensity time traces that mimic a realistic confocal setup, we simulate molecules moving [1, 30] through an illuminated 3D volume. The number of moving molecules N is prescribed in each simulation. To maintain a relatively stable concentration of molecules near the confocal volume, and so to avoid generating traces where every molecule eventually strays into un-illuminated regions, we impose periodic rectangular boundaries to our volume. The boundaries are placed at $\pm L_{\text{xy}}$ perpendicular to the focal plane and $\pm L_z$ perpendicular to the optical axis.

We assess the locations of the molecules x_k^n, y_k^n, z_k^n, where $k = 1,\dots,K$ label time levels and $n = 1,\dots,N$ label molecules, at equidistant time intervals $t_1, t_2, \dots, t_K$. The time interval between successive assessments $\delta t = t_k - t_{k-1}$, as well as the total trace duration $T_{\text{total}} = t_K - t_0$, are prescribed.

Molecule locations at the first assessment x_1^n, y_1^n, z_1^n are sampled randomly from a uniform distribution with limits equal to the boundaries $\pm L_{\text{xy}}$ and $\pm L_{\text{z}}$ of our pre-specified volume. Subsequent locations are generated according to the diffusion model described above under a prescribed diffusion coefficient D.

Finally, we obtain individual photon emissions w_k by simulating Bernoulli random variables of success probability $q_k = 1 - e^{-\mu_k \delta t}$, where the rate μ_k gathers single photon contributions from the background and the entire molecule population according to

$$\mu_k = \mu_{\text{back}} + \mu_{\text{mol}} \sum_{n=1}^{N} \text{PSF}(x_k^n, y_k^n, z_k^n) \qquad \text{Supplementary Equation 19}$$

where both background and molecular brightness, μ_{back} and μ_{mol}, are prescribed.

The PSF model is also prescribed. To avoid artifacts induced by the periodic boundaries we impose in our volume, we ensure that $L_{\text{xy}} \gg \omega_{\text{xy}}$, $L_{\text{z}} \gg \omega_{\text{z}}$, or $L_{\text{z}} \gg z_{\text{R}}$, where ω_{xy}, ω_{z} and z_{R} characterize the geometry of the confocal volume, see Supplementary Equation 16 – Supplementary Equation 18, above.

Detailed parameter choices for all simulations performed are listed in Supplementary Table 5.

7. Definition of normalized distance and numbers of molecules

As we need to estimate the positions of the molecules with respect to the center of the confocal volume, which is the point of origin, in order to ultimately estimate the number of molecules as a proxy for molecule concentration, for example Supplementary Figure 3 and Supplementary Figure 8, we must address difficulties associated with symmetries

of the confocal PSF with respect to rotations around the optical axis or the focal plane. [31] For this, for a molecule at (x_k^n, y_k^n, z_k^n), when the 3DG PSF is used, Supplementary Equation 16, we rely on

$$d_k^n = \sqrt{\left(\frac{x_k^n}{\omega_{\mathrm{xy}}}\right)^2 + \left(\frac{y_k^n}{\omega_{\mathrm{xy}}}\right)^2 + \left(\frac{z_k^n}{\omega_{\mathrm{z}}}\right)^2} \qquad \text{Supplementary Equation 20}$$

while, when the 2DGL PSF is used, Supplementary Equation 18, we rely on

$$d_k^n = \sqrt{\frac{\left(\frac{x_k^n}{\omega_{\mathrm{xy}}}\right)^2 + \left(\frac{y_k^n}{\omega_{\mathrm{xy}}}\right)^2}{1 + \left(\frac{z_k^n}{z_{\mathrm{R}}}\right)^2} + \frac{1}{2}\log\left(1 + \left(\frac{z_k^n}{z_{\mathrm{R}}}\right)^2\right)} \qquad \text{Supplementary Equation 21}$$

where d_k^n is the normalized distance with respect to the center of the confocal volume of molecule n at time k. Similarly, when a 2DGC PSF is used, Supplementary Equation 17, we rely on

$$d_k^n = \sqrt{\left(\frac{x_k^n}{\omega_{\mathrm{xy}}}\right)^2 + \left(\frac{y_k^n}{\omega_{\mathrm{xy}}}\right)^2} \qquad \text{Supplementary Equation 22}$$

where d_k^n is the normalized distance with respect to the optical axis of molecule n at time k.

These distances are obtained by setting the respective PSFs equal to $\exp(-(d_k^n)^2)$ and are unaffected by the aforementioned symmetries, i.e. $x_k^n \mapsto -x_k^n$, $y_k^n \mapsto -y_k^n$, and $z_k^n \mapsto -z_k^n$.

For a given normalized distance ℓ, we define the number of molecules N_k^ℓ as the number of estimated (active) molecules within the corresponding distance. That is

$$N_k^\ell = \sum_n b^n H\left(1 - \frac{d_k^n}{\ell}\right) \qquad \text{Supplementary Equation 23}$$

where H is the Heaviside step function, b^n is the load of molecule n, and V_ℓ is the volume of a designated effective region chosen to agree with the effective volume V_{eff} used in FCS [8].

8. Description of the time trace preparation

The initial time trace consists of single photon arrival times which are computationally too expensive to analyze. Our method instead operates on photon intensity traces which are either obtained directly during an experiment or obtained from individual photon arrival time traces after binning. To transform single photon arrival time traces into intensity time traces, we use time bins of fixed size (main size) that typically span multiple photon arrival times. To speed up the computations further, as some bins have none of very few photons, over certain portions of the trace we use larger bins (auxiliary size).

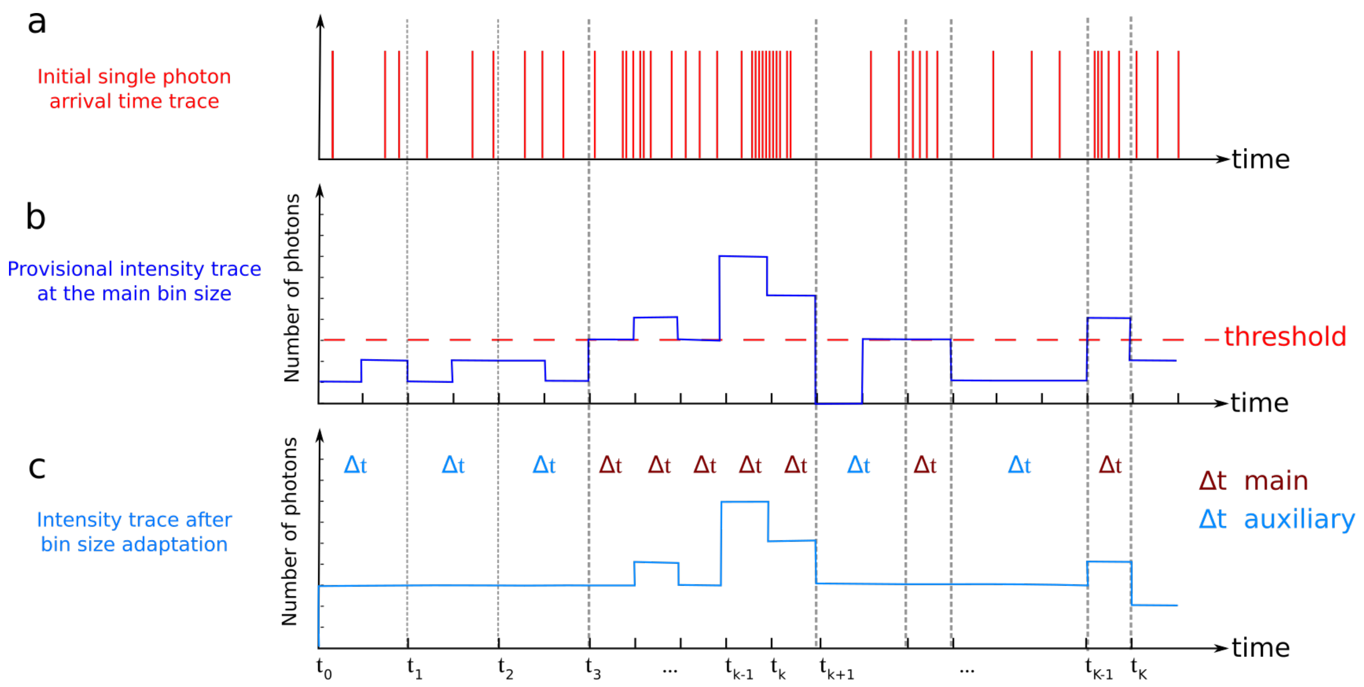

Supplementary Figure 14. Illustration of time trace preparation. a Initial trace of single photon arrivals. Here, each vertical line represents the arrival time of a single photon. **b** Time trace of photon intensities provisionally binned at the main bin size. The horizontal line denotes the imposed lower threshold on the minimum number of photons in the individual bins. **c** Time trace of photon intensities after bin size adaptation. Here, bins, preselected at the main size, with intensities below the imposed threshold are uniformly readjusted to achieve an average intensity similar to the threshold.

Briefly, the user specifies a minimum number of photons per bin as a lower threshold. As illustrated in Supplementary Figure 14, those bins, preselected at the main size, containing fewer photons than the specified threshold are enlarged uniformly in order to achieve an average of at least as many photons as specified by the threshold. This occasional adaptation, from the main to the auxiliary bins, becomes important in the analysis of traces from experiments held near single molecule resolution where molecule concentrations are low so that on average only one molecular passage through the confocal volume happens. Consequently, photon intensities are low, and thus the bulk of computational time otherwise would had been spent processing trace portions of poor quality (i.e. with few or no photons).

To carry our the necessary computations, as we detail shortly, we use the Anscombe transformation [32] to approximate the Poissonian likelihoods of photon intensities (see below). This approximation is robust as long as bins contain on average 4 photons or more. Thus, as a minimum requirement, we also use the aforementioned threshold to ensure the validity of the approximations.

Supplementary Note 4: Detailed description of the inference framework

1. Description of prior probability distributions

The model parameters in our framework that require priors are: the diffusion coefficient D; the molecular brightness and background photon emission rates μ_{mol} and μ_{back}; the initial molecule locations x_1^n,y_1^n,z_1^n; and load prior weights q^n. As we already mentioned in the main text, a prior on the population of diffusing molecules is implicitly defined by the prior on both b^n and q^n. Our choices are described below.

1.1. Prior on the diffusion coefficient

To ensure that the D sampled in our formulation attains only positive values, we place an inverse-Gamma prior

$$D \sim \textbf{InvGamma}\left(\alpha_D, \beta_D\right). \qquad \text{Supplementary Equation 24}$$

Besides ensuring a positive D, this prior is also conjugate to the motion model we use which facilitates the computations (see below).

1.2. Priors on molecular brightness and background photon emission rates

To ensure that μ_{mol} and μ_{back} sampled in our formulation attain only positive values, we place Gamma priors on both

$$\begin{aligned} \mu_{\text{mol}} &\sim \textbf{Gamma}\left(\alpha_{\text{mol}}, \beta_{\text{mol}}\right) \\ \mu_{\text{back}} &\sim \textbf{Gamma}\left(\alpha_{\text{back}}, \beta_{\text{back}}\right). \end{aligned} \qquad \text{Supplementary Equation 25}$$

Due to the specific dependencies of the likelihood (that we will discuss shortly) on the photon emission rates, conjugate priors cannot be achieved for μ_{mol} and μ_{back}. So, the above choice offers no computational advantage (see below) and could be readily replaced with more physically motivated choices if additional information on molecular brightness becomes available.

1.3. Priors on initial molecule locations

Due to the symmetries in the confocal PSF, i.e. a molecule at a location (x, y, z) emits the same average number of photons as a molecule at locations $(\pm x, \pm y, \pm z)$, we are unable to gain insight regarding the octant of the 3D Cartesian space in which each molecule is located. To avoid imposing further assumptions on our framework that may determine each molecule's octant uniquely, but may limit the framework's scope to specific experimental setups, we place priors on the initial locations that respect these symmetries. Accordingly, in our framework, at the onset of the measuring period, molecules are equally likely to be located at any of the positions $(\pm x_1^n, \pm y_1^n, \pm z_1^n)$.

To facilitate the computations (see below), we place independent symmetric normal distributions, see Supplementary Table 4, on each Cartesian coordinate of the model molecules

$$\begin{aligned} x_1^n &\sim \textbf{SymNormal}\left(\mu_{\text{xy}}, \sigma_{\text{xy}}^2\right) \\ y_1^n &\sim \textbf{SymNormal}\left(\mu_{\text{xy}}, \sigma_{\text{xy}}^2\right) \\ z_1^n &\sim \textbf{SymNormal}\left(\mu_{\text{z}}, \sigma_{\text{z}}^2\right). \end{aligned} \qquad \text{Supplementary Equation 26}$$

We want to emphasize that the symmetric priors above do not affect our estimates. According to the motion model we employ, no matter where molecules are initiated, they may subsequently move freely and eventually switch to a different octant if warranted by the data. Our symmetric priors merely indicate that for each individual molecular trajectory considered, there are another 7 symmetric trajectories that are equally likely to have occurred.

1.4. Priors and hyperpriors for molecule loads

To facilitate the computations (described next), we use a finite, but large, model population consisting of N molecules containing both active and inactive molecules. These model molecules are collectively indexed by $n =$

$1, 2, \ldots, N$. As explained in the main text, estimating how many molecules are actually warranted by the data under analysis is equivalent to estimating how many of those N molecules are active, i.e. $b^n = 1$, while the remaining inactive ones, i.e. $b^n = 0$, have no impact whatsoever and are instantiated only for computational purposes.

To ensure that each load b^n takes only values 0 or 1, we place a Bernoulli prior of weight q^n. In turn, on each weight q^n, we place a conjugate Beta hyperprior

$$b^n|q^n \sim \textbf{Bernoulli}(q^n) \quad \text{Supplementary Equation 27}$$
$$q^n \sim \textbf{Beta}\left(A_q, B_q\right). \quad \text{Supplementary Equation 28}$$

To ensure that the resulting formulation avoids overfitting, we make the specific choices $A_q = \alpha_q/N$ and $B_q = \beta_q(N-1)/N$. Under these choices [33–36], and in the limit that $N \to \infty$; that is, when the assumed molecule population is allowed to be large, this prior/hyperprior converge to a Beta-Bernoulli process. Consequently, for $N \gg 1$, the posterior remains well defined and becomes independent of the chosen value of N. In other words, provided N is large enough, its impact on the results is insignificant; while its precise value has only computational implications (see below).

2. Summary of model equations

For concreteness, below we summarize the entire set of equations used in our framework, including a complete list of priors and hyperpriors

$$D \sim \textbf{InvGamma}\left(\alpha_D, \beta_D\right) \quad \text{Supplementary Equation 29}$$

$$\mu_{\text{mol}} \sim \textbf{Gamma}\left(\alpha_{\text{mol}}, \beta_{\text{mol}}\right) \quad \text{Supplementary Equation 30}$$
$$\mu_{\text{back}} \sim \textbf{Gamma}\left(\alpha_{\text{back}}, \beta_{\text{back}}\right) \quad \text{Supplementary Equation 31}$$

$$q^n \sim \textbf{Beta}\left(\frac{\alpha_q}{N}, \beta_q \frac{N-1}{N}\right) \quad \text{Supplementary Equation 32}$$
$$b^n|q^n \sim \textbf{Bernoulli}(q^n) \quad \text{Supplementary Equation 33}$$

$$x_1^n \sim \textbf{SymNormal}\left(\mu_{\text{xy}}, \sigma_{\text{xy}}^2\right) \quad \text{Supplementary Equation 34}$$
$$y_1^n \sim \textbf{SymNormal}\left(\mu_{\text{xy}}, \sigma_{\text{xy}}^2\right) \quad \text{Supplementary Equation 35}$$
$$z_1^n \sim \textbf{SymNormal}\left(\mu_{\text{z}}, \sigma_{\text{z}}^2\right) \quad \text{Supplementary Equation 36}$$

$$x_k^n|x_{k-1}^n, D \sim \textbf{Normal}\left(x_{k-1}^n, 2(t_k - t_{k-1})D\right), \qquad k = 2, \ldots, K \quad \text{Supplementary Equation 37}$$
$$y_k^n|y_{k-1}^n, D \sim \textbf{Normal}\left(y_{k-1}^n, 2(t_k - t_{k-1})D\right), \qquad k = 2, \ldots, K \quad \text{Supplementary Equation 38}$$
$$z_k^n|z_{k-1}^n, D \sim \textbf{Normal}\left(z_{k-1}^n, 2(t_k - t_{k-1})D\right), \qquad k = 2, \ldots, K \quad \text{Supplementary Equation 39}$$

$$w_k|\{x_k^n, y_k^n, z_k^n, b^n\}_n, \mu_{\text{mol}}, \mu_{\text{back}} \sim \textbf{Poisson}\left(\mu_k\right), \qquad k = 1, \ldots, K \quad \text{Supplementary Equation 40}$$
$$\mu_k = (t_k - t_{k-1})\left(\mu_{\text{back}} + \mu_{\text{mol}} \sum_n b^n \ \text{PSF}(x_k^n, y_k^n, z_k^n)\right). \quad \text{Supplementary Equation 41}$$

For molecules diffusing in a confocal volume that is extremely elongated over the optical axis, the PSF approaches a cylindrical one. In this case, it is safe to eliminate the z_k^n positions from the motion model and simplify Supplementary Equation 40 and Supplementary Equation 41 to

$$w_k|\{x_k^n, y_k^n, b^n\}_n, \mu_{\text{mol}}, \mu_{\text{back}} \sim \textbf{Poisson}\left(\mu_k\right), \qquad k = 1, \ldots, K \quad \text{Supplementary Equation 42}$$
$$\mu_k = (t_k - t_{k-1})\left(\mu_{\text{back}} + \mu_{\text{mol}} \sum_n b^n \ \text{PSF}(x_k^n, y_k^n)\right). \quad \text{Supplementary Equation 43}$$

3. Description of the computational scheme

The joint probability distribution of our framework is $p(D, \mu_{\text{mol}}, \mu_{\text{back}}, \{q^n, b^n, \overline{x}^n, \overline{y}^n, \overline{z}^n\}_n | \overline{w})$, where molecular trajectories and intensities (measurements) are gathered in

$$\overline{x}^n = (x_1^n, x_2^n, \dots, x_K^n) \quad \text{Supplementary Equation 44}$$
$$\overline{y}^n = (y_1^n, y_2^n, \dots, y_K^n) \quad \text{Supplementary Equation 45}$$
$$\overline{z}^n = (z_1^n, z_2^n, \dots, z_K^n) \quad \text{Supplementary Equation 46}$$
$$\overline{w} = (w_1, w_2, \dots, w_K). \quad \text{Supplementary Equation 47}$$

Due to the nonlinearities in the PSF and the non-parametric prior on q^n and b^n, analytic evaluation or direct sampling of this posterior is impossible. For this reason, we develop a specialized Markov chain Monte Carlo (MCMC) scheme that can be used to generate pseudo-random samples [10, 11, 37–39]. This scheme is explained in detail below.

In order to terminate the MCMC sampler, we need to determine when a representative number of samples has been computed. To do so, we divide the samples already computed into four portions and compare the mean values of the diffusion coefficient of the two last ones

$$\eta_1 = \frac{\sum_{i=2I/4}^{3I/4} D_i}{I/4}, \qquad \eta_2 = \frac{\sum_{i=3I/4}^{I} D_i}{I/4} \quad \text{Supplementary Equation 48}$$

where, η_1 and η_2 are the mean values of the two last portion of the sampled diffusion coefficients denoted D_i and I is the total number of computed MCMC samples thus far. Following [37, 38], we terminate the sampler when $|\eta_1 - \eta_2| < \epsilon_{\text{thr}}$, where ϵ_{thr} is a pre-specified threshold. Also, to avoid incorporating burn-in samples in the calculations, we ensure a minimum number of iterations I of no less than 10^4.

A working implementation of the resulting scheme in source code and GUI forms, see Supplementary Figure 15, are available through the provided source code.

3.1. Overview of the sampling updates

Our MCMC exploits a Gibbs sampling scheme [10, 37, 38]. Accordingly, posterior samples are generated by updating each one of the variables involved sequentially by sampling conditioned on all other variables and measurements $\overline{w}$.

Conceptually, the steps involved in the generation of each posterior sample $(D, \mu_{\text{mol}}, \mu_{\text{back}}, \{q^n, b^n, \overline{x}^n, \overline{y}^n, \overline{z}^n\}_n)$ are:

(1) For each n in the molecule population
  (i) Update trajectory $\overline{x}^n$ of molecule n
  (ii) Update trajectory $\overline{y}^n$ of molecule n
  (iii) Update trajectory $\overline{z}^n$ of molecule n
(2) Update the diffusion coefficient D
(3) Update jointly the prior weights q^n for all molecules
(4) Update jointly the loads b^n for all molecules
(5) Update jointly the molecular brightness and background photon emission rates μ_{mol} and μ_{back}, respectively

Since the locations of the inactive molecules are not associated with the measurements $\overline{w}$, see 8, and those are updated independently of the locations of the active ones, to make the algorithm computationally more efficient we carry out the above scheme in the equivalent order

(1) For each n of the active molecules
  (i) Update trajectory $\overline{x}^n$ of active molecule n
  (ii) Update trajectory $\overline{y}^n$ of active molecule n
  (iii) Update trajectory $\overline{z}^n$ of active molecule n
(2) Update jointly the trajectories $\overline{x}^n, \overline{y}^n, \overline{z}^n$ for all n of the inactive molecules
(3) Update the diffusion coefficient D
(4) Update jointly the prior weights q^n for all model molecules

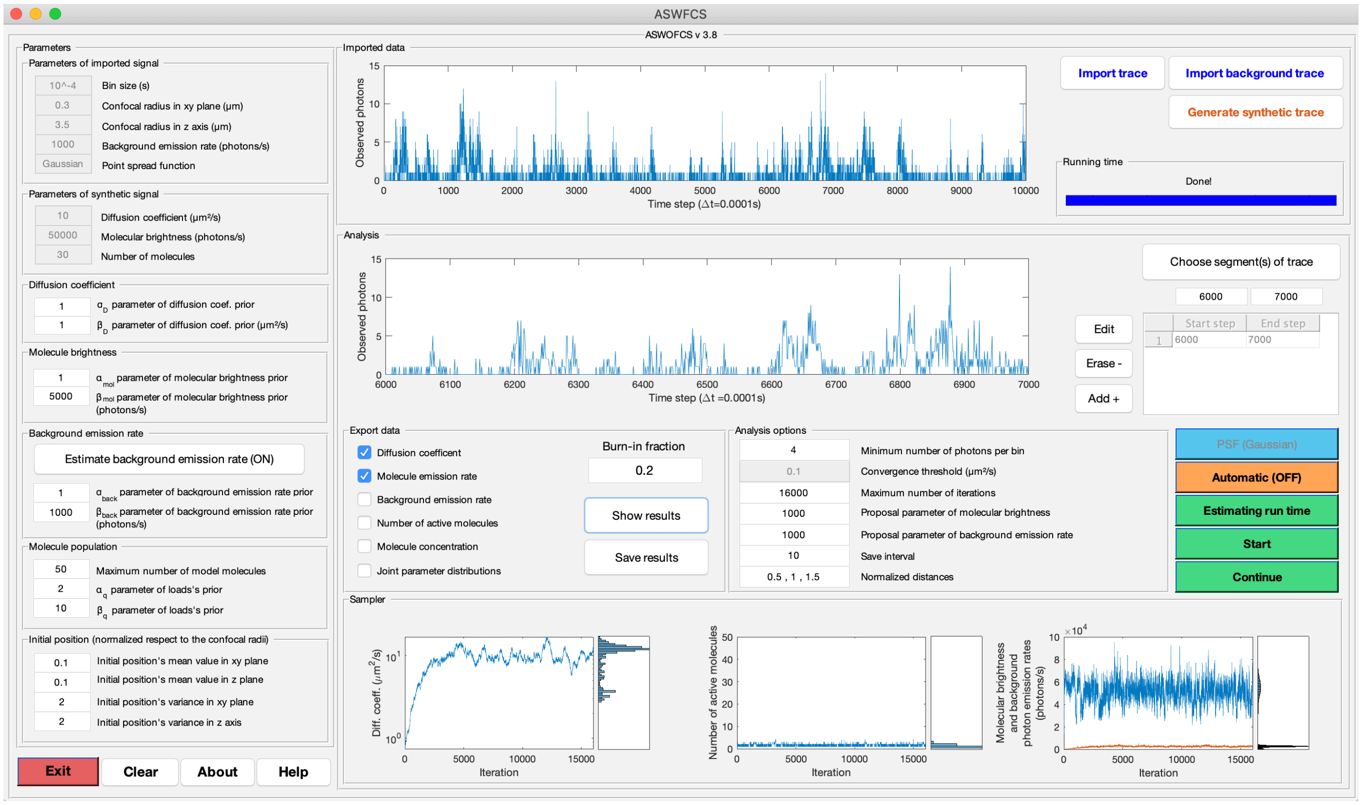

Supplementary Figure 15. A working implementation of the framework described in this study is available through the provided source code. Along with this implementation, we provide a graphical user interface (GUI) that can be used to analyze intensity traces from confocal microscopy.

(5) Update jointly the loads b^n for all model molecules

(6) Update jointly the molecular brightness and background photon emission rates μ_{mol} and μ_{back}, respectively

These steps are described in detail below.

3.2. Sampling of active molecule trajectories

For a given active molecule n, we update the trajectory $\overline{x}^n$ by sampling from the corresponding conditional $p(\overline{x}^n|D, \mu_{\text{mol}}, \mu_{\text{back}}, \{b^{n'}, \overline{y}^{n'}, \overline{z}^{n'}\}_{n'}, \{\overline{x}^{n'}\}_{n'\neq n}, \overline{w})$, which we achieved through backward sampling [40–42]. To be able to sample a trajectory $\overline{x}^n$ in backward sampling, we factorize the density $p(\overline{x}^n|D, \mu_{\text{mol}}, \mu_{\text{back}}, \{b^{n'}, \overline{y}^{n'}, \overline{z}^{n'}\}_{n'}, \{\overline{x}^{n'}\}_{n'\neq n}, \overline{w})$ as

$$\begin{aligned}
&p(\overline{x}^n|D, \mu_{\text{mol}}, \mu_{\text{back}}, \{b^{n'}, \overline{y}^{n'}, \overline{z}^{n'}\}_{n'}, \{\overline{x}^{n'}\}_{n'\neq n}, \overline{w}) \\
&= p(x_1^n|x_2^n, D, \mu_{\text{mol}}, \mu_{\text{back}}, \{b^{n'}, y_1^{n'}, z_1^{n'}\}_{n'}, \{x_1^{n'}\}_{n'\neq n}, \overline{w}) \\
&\times p(x_2^n|x_3^n, D, \mu_{\text{mol}}, \mu_{\text{back}}, \{b^{n'}, y_2^{n'}, z_2^{n'}\}_{n'}, \{x_2^{n'}\}_{n'\neq n}, \overline{w}) \\
&\vdots \\
&\times p(x_{K-1}^n|x_K^n, D, \mu_{\text{mol}}, \mu_{\text{back}}, \{b^{n'}, y_{K-1}^{n'}, z_{K-1}^{n'}\}_{n'}, \{x_{K-1}^{n'}\}_{n'\neq n}, \overline{w}) \\
&\times p(x_K^n|D, \mu_{\text{mol}}, \mu_{\text{back}}, \{b^{n'}, y_K^{n'}, z_K^{n'}\}_{n'}, \{x_K^{n'}\}_{n'\neq n}, \overline{w}).
\end{aligned}$$

Supplementary Equation 49

According to this factorization, we sample $\overline{x}^n$, starting from x_K^n and move backward towards x_1^n. To start the sampling steps, we need to determine each one of the individual densities $p(x_K^n|D, \mu_{\text{mol}}, \mu_{\text{back}}, \{b^{n'}, y_K^{n'}, z_K^{n'}\}_{n'}, \{x_K^{n'}\}_{n'\neq n}, \overline{w})$ and $p(x_k^n|x_{k+1}^n, D, \mu_{\text{mol}}, \mu_{\text{back}}, \{b^{n'}, y_k^{n'}, z_k^{n'}\}_{n'}, \{x_k^{n'}\}_{n'\neq n}, \overline{w})$. We do this in a forward filtering approach [31, 40–44] which is described in detail below.

a. Forward filtering

By applying Bayes' rule, each one of the individual densities in Supplementary Equation 49 factorizes as

$$p(x_k^n|x_{k+1}^n, D, \dots, \overline{w}) \propto p(x_{k+1}^n|x_k^n, D)p(x_k^n|D, \dots, w_{1:k}) \qquad \text{Supplementary Equation 50}$$

where $w_{1:k}$ is an abbreviation for $w_1, \dots, w_k$ and excess parameters are shown by dots. Since the density $p(x_{k+1}^n|x_k^n, D)$ is already known, to sample x_k^n in backward sampling, we only need to determine the filter density $p(x_k^n|D, \dots, w_{1:k})$.

To be able to apply forward filtering and compute $p(x_k^n|D, \dots, w_{1:k})$ efficiently [45], we use an approximate model [46], where Supplementary Equation 40, is replaced with

$$\mathbb{T}_{\text{data}}(w_k)|\{x_k^{n'}, y_k^{n'}, z_k^{n'}, b^{n'}\}_{n'}, \mu_{\text{mol}} \sim \textbf{Normal}\left(\mathbb{T}_{\text{mean}}(\mu_k), 1\right), \qquad k = 1, \dots, K. \qquad \text{Supplementary Equation 51}$$

Here, μ_k stems from Supplementary Equation 41 for 3D models and Supplementary Equation 43 for 2D models; while, $\mathbb{T}_{\text{data}}(w)$ and $\mathbb{T}_{\text{mean}}(\mu)$ denote Anscombe transformed [32] variables defined as follows

$$\mathbb{T}_{\text{data}}(w) = 2\sqrt{w + \frac{3}{8}} \qquad \text{Supplementary Equation 52}$$

$$\mathbb{T}_{\text{mean}}(\mu) = 2\sqrt{\mu + \frac{3}{8}} - \frac{1}{4\sqrt{\mu}}. \qquad \text{Supplementary Equation 53}$$

The Anscombe transform exploited here offers a way of transforming Poisson random variables into (approximately) normal ones [32] which facilitates the filtering process described next. The approximation we employ is highly accurate for $\mu \gg 1$, while acceptable accuracy is maintained so long as $\mu > 4$ photons. Increasing the accuracy of the above approximation is achieved with either longer data acquisition times or higher laser powers.

Under the Anscombe transform, the densities of both the dynamics, Supplementary Equation 37, and observations, Supplementary Equation 51, are normally distributed. So, we can compute the filter distribution $p(x_k^n|D, \dots, w_{1:k})$ of the approximate model similar to the standard theory underlying nonlinear Kalman filters [31, 47–53].

More specifically, because the mean of the transformed observation distribution, $T_{\text{mean}}(\mu_k)$ is a nonlinear function of the location x_k^n, to apply the Kalman filters we need to approximate the transformed observation distribution in such a way that its mean becomes a linear function of the location x_k^n. To do so, we use two common approaches: (i) extended Kalman filter (EKF) [47, 54–57], which locally approximate the transformed observation distribution around selected values; and (ii) unscented Kalman filter (UKF) [48–50], which globally approximate the transformed observation distribution.

As explained in detail in [31], the linearization alone is not sufficient to properly approximate the filter. This is because both EKF and UKF assume that the filter is a normal density. This assumption is problematic for our particular case which is symmetric across the origin, i.e. observations provide equal probabilities for the molecule to be in negative or positive side of the center of the PSF, i.e. $\pm x_k^n$. Due to this symmetry across the yz-plane, the filtering distribution consists of two modes centered symmetrically across the origin [31]. Therefore, we compute an approximate filter distribution of the form

$$p\left(x_k^n|D, \dots, w_{1:k}\right) \approx \textbf{SymNormal}\left(x_k^n; m_k^n, c_k^n\right) \qquad \text{Supplementary Equation 54}$$

where $\textbf{SymNormal}\left(m_k^n, c_k^n\right)$ denotes the symmetric normal distribution (see Supplementary Table 4). The filter's parameters m_k^n and c_k^n can be computed recursively according to

$$p\left(x_k^n|D, \dots, w_{1:k}\right) \propto p\left(w_k|x_k^n, y_k^n, z_k^n, \mu_{\text{mol}}, \mu_{\text{back}}, \{b^{n'}, x^{n'}, y^{n'}, z^{n'}\}_n'\right) \times \int_{x_{k-1}^n} p\left(x_{k-1}^n|D, \dots, w_{1:k-1}\right) p\left(x_k^n|x_{k-1}^n, D\right) dx_{k-1}^n \qquad \text{Supplementary Equation 55}$$

which, for our model, reduces to

$$p\left(x_k^n|D, \dots, w_{1:k}\right) \propto \textbf{Normal}\left(\mathbb{T}_{\text{data}}(w_k); \mathbb{T}_{\text{mean}}(\mu_k), 1\right) \textbf{SymNormal}\left(x_k^n; m_{k-1}^n, c_{k-1}^n + 2D\left(t_k - t_{k-1}\right)\right) \qquad \text{Supplementary Equation 56}$$

and, in turn, is approximated as

$$p\left(x_k^n|D, \dots, w_{1:k}\right) \approx \textbf{SymNormal}\left(x_k^n; m_k^n, c_k^n\right). \qquad \text{Supplementary Equation 57}$$

To summarize, in the forward pass of the FFBS, we compute m_k^n and c_k^n of the filter of the molecule n, for all time levels $k = 1, \dots, K$, by linearizing the approximate model around $x_1^n = \mu_{\text{xy}}$ for $k = 1$, and around $x_k^n = m_{k-1}^n$

for $k = 2, \ldots, K$. Since our observation is nonlinear, to calculate the filter, we opt between two different methods: **(i)** Extended Kalman filter (EKF) and **(ii)** Unscented Kalman filter (UKF).

In the EKF, we linearize the observations to obtain a closed form for the filter (local approximation) and in the UKF we approximate the joint probability distribution of observations and locations with a multivariate normal distribution (global approximation). The reason to use either of these filters is that the EKF is computationally cheaper but less accurate. According to our analysis it may fail to provide unbiased estimates of the background photon emission rate. On the other hand, the UKF is more robust and provides background emission rate estimates, but these benefits come at an increased computational cost.

In this study, we provide both filters and allow the user to choose between them.

Extended Kalman filter

Within the EKF approximation, the normal probability distribution preceding the symmetric normal of Supplementary Equation 56 is linearized in order for their product to become a symmetric normal one.

In this case, we linearize the mean of the observation density $\mathbf{Normal}\left(\mathbb{T}_{\text{data}}(w_k); \mathbb{T}_{\text{mean}}(\mu_k), 1\right)$, around the modes of the filter in the previous time step

$$\begin{aligned} \mathbb{T}_{\text{mean}}\left(\mu_k\left(x_k^n\right)\right) &\approx \mathbb{T}_{\text{mean}}\left(\mu_k\left(-m_{k-1}^n\right)\right) + \frac{\partial \mathbb{T}_{\text{mean}}\left(\mu_k\left(x_k^n\right)\right)}{\partial x_k^n}\bigg|_{x_k^n=-m_{k-1}^n}\left(x_k^n + m_{k-1}^n\right) \\ \mathbb{T}_{\text{mean}}\left(\mu_k\left(x_k^n\right)\right) &\approx \mathbb{T}_{\text{mean}}\left(\mu_k\left(+m_{k-1}^n\right)\right) + \frac{\partial \mathbb{T}_{\text{mean}}\left(\mu_k\left(x_k^n\right)\right)}{\partial x_k^n}\bigg|_{x_k^n=+m_{k-1}^n}\left(x_k^n - m_{k-1}^n\right) \end{aligned} \qquad \text{Supplementary Equation 58}$$

where the first term linearizes around $x_k^n = -m_{k-1}^n$ and the second term linearizes around $x_k^n = +m_{k-1}^n$. Under these approximations, Supplementary Equation 56 attains an analytical solution. In detail

$$\begin{aligned} &\mathbf{Normal}\left(\mathbb{T}_{\text{data}}(w_k); \mathbb{T}_{\text{mean}}(\mu_k), 1\right) \mathbf{SymNormal}\left(x_k^n; m_{k-1}^n, c_{k-1}^n + 2D\left(t_k - t_{k-1}\right)\right) \\ &= \mathbf{Normal}\left(\mathbb{T}_{\text{data}}(w_k); \mathbb{T}_{\text{mean}}(\mu_k), 1\right) \mathbf{Normal}\left(x_k^n; -m_{k-1}^n, c_{k-1}^n + 2D\left(t_k - t_{k-1}\right)\right) \\ &+ \mathbf{Normal}\left(\mathbb{T}_{\text{data}}(w_k); \mathbb{T}_{\text{mean}}(\mu_k), 1\right) \mathbf{Normal}\left(x_k^n; +m_{k-1}^n, c_{k-1}^n + 2D\left(t_k - t_{k-1}\right)\right) \\ &= \mathbf{Normal}\left(x_k^n; -m_{k-1}^n + \frac{e_k^n}{d_k^n}, \frac{1}{(d_k^n)^2}\right) \mathbf{Normal}\left(x_k^n; -m_{k-1}^n, c_{k-1}^n + 2D\left(t_k - t_{k-1}\right)\right) \\ &+ \mathbf{Normal}\left(x_k^n; +m_{k-1}^n - \frac{e_k^n}{d_k^n}, \frac{1}{(d_k^n)^2}\right) \mathbf{Normal}\left(x_k^n; +m_{k-1}^n, c_{k-1}^n + 2D\left(t_k - t_{k-1}\right)\right) \\ &= \frac{1}{2}\mathbf{Normal}\left(x_k^n; -m_k^n, c_k^n\right) + \frac{1}{2}\mathbf{Normal}\left(x_k^n; +m_k^n, c_k^n\right) \\ &= \mathbf{SymNormal}\left(x_k^n; m_k^n, c_k^n\right). \end{aligned} \qquad \text{Supplementary Equation 59}$$

The same calculations apply also for $k = 1$, where the starting density is replaced with the prior of Supplementary Equation 26. In this case

$$\begin{aligned} c_1^n &= \frac{\sigma_{\text{xy}}^2}{\mathbb{S}(\mu_1)^2 + \sigma_{\text{xy}}^2 (d_1^n)^2} \\ m_1^n &= \mu_{\text{xy}} + c_1^n d_1^n e_1^n \\ d_1^n &= \frac{\partial \mathbb{T}_{\text{mean}}(\mu_1\left(x_1^n\right))}{\partial x_1^n}\bigg|_{x_1^n=\mu_{\text{xy}}} \\ e_1^n &= \mathbb{T}_{\text{data}}(w_1) - \mathbb{T}_{\text{mean}}(\mu_1\left(x_1^n\right))\Big|_{x_1^n=\mu_{\text{xy}}} \end{aligned} \qquad \text{Supplementary Equation 60}$$

while for $k = 2, \ldots, K$ are

$$\begin{aligned} c_k^n &= \frac{\left(c_{k-1}^n + 2D\left(t_k - t_{k-1}\right)\right)}{1 + \left(c_{k-1}^n + 2D\left(t_k - t_{k-1}\right)\right)(d_k^n)^2} \\ m_k^n &= m_{k-1}^n + c_k^n d_k^n e_k^n \\ d_k^n &= \frac{\partial \mathbb{T}_{\text{mean}}(\mu_k\left(x_k^n\right))}{\partial x_k^n}\bigg|_{x_k^n=m_{k-1}^n} \\ e_k^n &= \mathbb{T}(w_k) - \mathbb{T}(\mu_k\left(x_k^n\right))\Big|_{x_k^n=m_{k-1}^n}. \end{aligned} \qquad \text{Supplementary Equation 61}$$

Unscented Kalman filter

The unscented Kalman filter [48, 49] tries to fit the joint probability distribution of the observations and locations globally with a multivariate normal distribution to cope with the nonlinearity in Supplementary Equation 56. Specifically the product of Supplementary Equation 56 is approximated as follows

$$\begin{aligned}
&\mathbf{Normal}\left(\mathbb{T}_{\text{data}}(w_k); \mathbb{T}_{\text{mean}}(\mu_k), 1\right)\mathbf{SymNormal}\left(x_k^n; m_{k-1}^n, c_{k-1}^n + 2D\left(t_k - t_{k-1}\right)\right) \\
&\approx \frac{1}{2}\mathbf{BNormal}\left(\begin{bmatrix} x_k^n \\ \mathbb{T}_{\text{data}}(w_k)\end{bmatrix}; \begin{bmatrix} -X_k^n \\ W_k^n \end{bmatrix}, \begin{bmatrix} {}_{xx}\Sigma_k^n, & -{}_{xw}\Sigma_k^n \\ -{}_{wx}\Sigma_k^n, & {}_{ww}\Sigma_k^n \end{bmatrix}\right) \\
&+ \frac{1}{2}\mathbf{BNormal}\left(\begin{bmatrix} x_k^n \\ \mathbb{T}_{\text{data}}(w_k)\end{bmatrix}; \begin{bmatrix} +X_k^n \\ W_k^n \end{bmatrix}, \begin{bmatrix} {}_{xx}\Sigma_k^n, & +{}_{xw}\Sigma_k^n \\ +{}_{wx}\Sigma_k^n, & {}_{ww}\Sigma_k^n \end{bmatrix}\right) \\
&\propto \frac{1}{2}\mathbf{Normal}\left(x_k^n; -m_k^n, c_k^n\right) + \frac{1}{2}\mathbf{Normal}\left(x_k^n; +m_k^n, c_k^n\right) \\
&= \mathbf{SymNormal}\left(x_k^n; m_k^n, c_k^n\right)
\end{aligned}$$

Supplementary Equation 62

Since we are faced with a filter which has two symmetric modes, we calculate the filter's mean m_k^n and variance c_k^n for one of the modes only, while we recover the other mode's mean and variance by reflection.

The means, auto- and cross-covariances in one mode of the Supplementary Equation 62 are given by

$$\begin{aligned}
X_k^n &= \int_{-\infty}^{+\infty} x q(x) dx \\
W_k^n &= \int_{-\infty}^{+\infty} \mathbb{T}_{\text{mean}}\left(\mu_k\left(x\right)\right) q(x) dx \\
{}_{xx}\Sigma_k^n &= \int_{-\infty}^{+\infty} (x - X_k^n)^T (x - X_k^n) q(x) dx \\
{}_{ww}\Sigma_k^n &= \int_{-\infty}^{+\infty} (\mathbb{T}_{\text{data}}(\mu_k) - W_k^n)^T (\mathbb{T}_{\text{data}}(\mu_k) - W_k^n) q(x) dx + 1 \\
{}_{xw}\Sigma_k^n &= \int_{-\infty}^{+\infty} \left(x - X_k^n\right)^T \left(\mathbb{T}_{\text{data}}(\mu_k) - W_k^n\right) q(x) dx \\
{}_{wx}\Sigma_k^n &= \int_{-\infty}^{+\infty} \left(\mathbb{T}_{\text{data}}(\mu_k) - W_k^n\right)^T \left(x - X_k^n\right) q(x) dx
\end{aligned}$$

Supplementary Equation 63

where $q(x) = \mathbf{Normal}\left(x; m_{k-1}^n, c_{k-1}^n + 2D\left(t_k - t_{k-1}\right)\right)$ is the probability density of one mode of the filter. The same formula applies to the other mode too.

To calculate the mean value m_k^n and variance c_k^n of each normal contributing to the symmetric normal shown above, we need to specify a set of sample points, termed sigma points in the UKF literature [48–50, 58–60], to estimate the mean values and covariance matrix of the bivariate normal on which m_k^n and c_k^n depend. To specify sigma points, we first calculate sigma points x_i^{sn} and their weights g_i^* for a standard normal $\mathbf{Normal}(x; 0, 1)$ as following

i	1	2	3	4	5	6	7	8	9	10	11
x_i^{sn}	-5.1880	-3.9362	-2.8651	-1.8760	-0.9289	0	0.9289	1.8760	2.8651	3.9362	5.1880
g_i^*	$< 10^{-5}$	0.0002	0.0067	0.0661	0.2422	0.3694	0.2422	0.0661	0.0067	0.0002	$< 10^{-5}$

according to [61]. We then transform x_i^{sn} that will be used in this $\mathbf{Normal}(x; m_{k-1}, c_{k-1} + 2D(t_k - t_{k-1}))$. The transformed sigma points are

$$x_i^* = m_{k-1} + x_i^{sn}\sqrt{c_{k-1} + 2D\left(t_k - t_{k-1}\right)}.$$

Supplementary Equation 64

Finally, given g_i^*, x_i^*, we calculate the mean and covariance of the bivariate normal previously introduced by

$$
\begin{aligned}
X_k^n &= \int_{-\infty}^{\infty} xq(x)dx \approx \sum_i g_i^* x_i^* \\
W_k^n &= \int_{-\infty}^{\infty} \mathbb{T}_x\left(x\right) q(x)dx \approx \sum_i g_i^* \mathbb{T}_x\left(x_i^*\right) \\
{}_{xx}\Sigma_k^n &= \int_{-\infty}^{\infty} (x -_x M_k)^T (x -_x M_k) q(x)dx \approx \sum_i g_i^* \left({}_xM_k - x_i^*\right)^T \left({}_xM_k - x_i^*\right) \\
{}_{ww}\Sigma_k^n &= \int_{-\infty}^{\infty} \left(\mathbb{T}_x\left(x\right) -_w M_k\right)^T \left(\mathbb{T}_x\left(x\right) -_w M_k\right) q(x)dx + 1 \approx \sum_i g_i^* \left({}_wM_k - \mathbb{T}_x\left(x_i^*\right)\right)^T \left({}_wM_k - \mathbb{T}_x\left(x_i^*\right)\right) + 1 \\
{}_{xw}\Sigma_k^n &= \int_{-\infty}^{\infty} (x -_x M_k)^T (\mathbb{T}_x\left(x\right) -_w M_k) q(x)dx \approx \sum_i g_i^* \left({}_wM_k - \mathbb{T}_x\left(x_i^*\right)\right)^T \left({}_xM_k - x_i^*\right) \\
{}_{wx}\Sigma_k^n &= \int_{-\infty}^{\infty} (\mathbb{T}_x\left(x\right) -_w M_k)^T (x -_x M_k) q(x)dx \approx \sum_i g_i^* \left({}_xM_k - x_i^*\right)^T \left({}_wM_k - \mathbb{T}_x\left(x_i^*\right)\right).
\end{aligned}
$$

Supplementary Equation 65

After computing the means X_k^n and W_k^n and auto-covariances and cross-covariances ${}_{xx}\Sigma_k$, ${}_{ww}\Sigma_k$, ${}_{xw}\Sigma_k$, ${}_{wx}\Sigma_k$, the mean and variance of each mode of the filter are given by

$$m_k^n = X_k^n + K_k^n \left(\mathbb{T}_{\text{data}}(w_k) - W_k^n\right) \quad \text{Supplementary Equation 66}$$

$$c_k^n =_{xx} \Sigma_k^n - K_k^n ({}_{ww}\Sigma_k^n) K_k^{nT} \quad \text{Supplementary Equation 67}$$

$$K_k^n = \frac{{}_{xw}\Sigma_k^n}{{}_{ww}\Sigma_k^n}. \quad \text{Supplementary Equation 68}$$

b. Backward sampling

After we compute the filter densities $p\left(x_k^n | D, \ldots, w_{1:k}\right)$ in the forward filtering step, through the EKF or UKF, we are able to sample the location x_k^n by using backward sampling as in Supplementary Equation 50. Specifically, given a computed filter, we sample sequentially x_k^n according to

$$x_K^n \sim p\left(x_K^n | \{x_{k'}^n\}_{k'<K}, D, \mu_{\text{mol}}, \mu_{\text{back}}, \{b^{n'}, \overline{y}^{n'}, \overline{z}^{n'}\}_{n'}, \{\overline{x}^{n'}\}_{n' \neq n}, \overline{w}\right) \quad \text{Supplementary Equation 69}$$

$$x_k^n \sim p\left(x_k^n | x_{k+1}^n, \{x_{k'}^n\}_{k'<k}, D, \mu_{\text{mol}}, \mu_{\text{back}}, \{b^{n'}, \overline{y}^{n'}, \overline{z}^{n'}\}_{n'}, \{\overline{x}^{n'}\}_{n' \neq n}, \overline{w}\right), \qquad k = 1, \ldots, K-1.$$

Supplementary Equation 70

Due to the specific choices of our problem these reduce to

$$x_K^n \sim \textbf{SymNormal}\left(m_K^n, c_K^n\right) \quad \text{Supplementary Equation 71}$$

$$x_k^n \sim \textbf{SymNormal}\left(m_k^n, c_k^n\right) \times \textbf{Normal}\left(x_{k+1}^n, 2D(t_{k+1} - t_k)\right), \qquad k = 1, \ldots, K-1 \quad \text{Supplementary Equation 72}$$

where m_k^n and c_k^n are the parameters of the filter which are calculated in the forward filtering step.

3.3. Sampling of inactive molecule trajectories

After updating the trajectories of the active molecules, we update the trajectories of the inactive ones. For this, we sample from the corresponding conditionals $p(\{\overline{x}^n, \overline{y}^n, \overline{z}^n\}_{n:b^n=0} | D, \mu_{\text{mol}}, \mu_{\text{back}}, \{q^n, b^n\}_n, \overline{w})$. Since the locations of inactive molecules are not associated with the observations in $\overline{w}$ and hyper-priors $\{q^n\}_n$, these conditionals simplify to $p(\{\overline{x}^n, \overline{y}^n, \overline{z}^n\}_{n:b^n=0} | D, \{b^n\}_n)$ which can be readily simulated jointly in the same manner as standard 3D Brownian motion.

3.4. Sampling of the diffusion coefficient

Now that we have updated the locations of active and inactive molecules, we update the diffusion coefficient D by sampling from the corresponding conditional $p(D | \mu_{\text{mol}}, \mu_{\text{back}}, \{q^n, b^n, \overline{x}^n, \overline{y}^n, \overline{z}^n\}_n, \overline{w})$, which, due to the spe-

cific dependencies of the variables in our formulation, e.g. Supplementary Equation 24, Supplementary Equation 37, Supplementary Equation 38 and Supplementary Equation 39, simplifies to $p(D|\{\overline{x}^n, \overline{y}^n, \overline{z}^n\}_n)$. Because of conjugacy, the latter reduces to

$$D|\{\overline{x}^n, \overline{y}^n, \overline{z}^n\}_n \sim \mathbf{InvGamma}\left(\alpha', \beta'\right) \quad \text{Supplementary Equation 73}$$

where α' and β' are given by

$$\alpha' = \alpha_D + \frac{3N(K-1)}{2}, \quad \beta' = \beta_D + \frac{1}{4}\sum_{n=1}^{N}\sum_{k=1}^{K-1} \frac{\left(x_{k+1}^n - x_k^n\right)^2 + \left(y_{k+1}^n - y_k^n\right)^2 + \left(z_{k+1}^n - z_k^n\right)^2}{t_{k+1} - t_k} \quad \text{Supplementary Equation 74}$$

3.5. Sampling of the molecule prior weights and loads

We update the load prior weights q^n by sampling from the corresponding conditional $p(\{q^n\}_n|D, \mu_{\text{mol}}, \mu_{\text{back}}, \{b^n, \overline{x}^n, \overline{y}^n, \overline{z}^n\}_n, \overline{w})$, which simplifies to $p(\{q^n\}_n|\{b^n\}_n)$. For this, we use Supplementary Equation 33 and Supplementary Equation 32, and because of conjugacy, the latter distribution is sampled by sampling each q^n separately according to

$$p(q^n|b^n) \propto p(b^n|q^n)p(q^n) = \mathbf{Beta}\left(q^n; \frac{\alpha_q}{N} + b^n, \frac{\beta_q(N-1)}{N} + 1 - b^n\right). \quad \text{Supplementary Equation 75}$$

Once the weights q^n are updated, we update the loads b^n by sampling from the corresponding conditional $p(\{b^n\}_n|D, \mu_{\text{mol}}, \mu_{\text{back}}, \{q^n, \overline{x}^n, \overline{y}^n, \overline{z}^n\}_n, \overline{w})$. We perform this sampling using a Metropolis-Hastings update with proposals of the form

$$(b^n)^{\text{prop}} \sim \mathbf{Bernoulli}(q^n). \quad \text{Supplementary Equation 76}$$

In this case, by choosing the proposal distribution similar to the prior distribution, the acceptance ratio becomes

$$\begin{aligned} r_b = \prod_{k=1}^{K} \Bigg[& \left(\frac{\mu_{\text{back}} + \mu_{\text{mol}} \sum_{n=1}^{N} (b^n)^{\text{prop}}\, \mathbf{PSF}\left(x_k^n, y_k^n, z_k^n\right)}{\mu_{\text{back}} + \mu_{\text{mol}} \sum_{n=1}^{N} (b^n)^{\text{old}}\, \mathbf{PSF}\left(x_k^n, y_k^n, z_k^n\right)}\right)^{w_k} \\ & \times \exp\left(-(t_k - t_{k-1})\mu_{\text{mol}} \sum_{n=1}^{N} \left((b^n)^{\text{old}} - (b^n)^{\text{prop}}\right) \mathbf{PSF}\left(x_k^n, y_k^n, z_k^n\right)\right)\Bigg] \end{aligned} \quad \text{Supplementary Equation 77}$$

where $(b^n)^{\text{old}}$ denotes the existing sample.

3.6. Joint sampling of the molecular brightness and background photon emission rates

Finally, after we updated the locations of molecules, and loads, we update the molecular brightness and background photon emission rates μ_{mol} and μ_{back} by sampling from the corresponding conditional $p(\mu_{\text{mol}}, \mu_{\text{back}}|D, \{q^n, b^n, \overline{x}^n, \overline{y}^n, \overline{z}^n\}_n, \overline{w})$, which simplifies to $p(\mu_{\text{mol}}, \mu_{\text{back}}|\{b^n, \overline{x}^n, \overline{y}^n, \overline{z}^n\}_n, \overline{w})$. We carry over this sampling using a Metropolis-Hastings update where proposals for μ_{mol} and μ_{back} are computed according to

$$\begin{aligned} \mu_{\text{mol}}^{\text{prop}} &\sim \mathbf{Gamma}\left(\alpha_{\text{mol}}^{\text{prop}}, \frac{\mu_{\text{mol}}^{\text{old}}}{\alpha_{\text{mol}}^{\text{prop}}}\right) \\ \mu_{\text{back}}^{\text{prop}} &\sim \mathbf{Gamma}\left(\alpha_{\text{back}}^{\text{prop}}, \frac{\mu_{\text{back}}^{\text{old}}}{\alpha_{\text{back}}^{\text{prop}}}\right) \end{aligned} \quad \text{Supplementary Equation 78}$$

where $\mu_{\rm mol}^{\rm old}$ and $\mu_{\rm back}^{\rm old}$ denote the existing samples. The acceptance ratio is

$$r_\mu = \prod_{k=1}^{K}\left[\left(\frac{\mu_{\rm back}^{\rm prop}+\mu_{\rm mol}^{\rm prop}\sum_{n=1}^{N} b^n\mathbf{PSF}\left(x_k^n,y_k^n,z_k^n\right)}{\mu_{\rm back}^{\rm old}+\mu_{\rm mol}^{\rm old}\sum_{n=1}^{N} b^n\mathbf{PSF}\left(x_k^n,y_k^n,z_k^n\right)}\right)^{w_k} \times \exp\left(\left(t_k-t_{k-1}\right)\left(\left(\mu_{\rm back}^{\rm old}-\mu_{\rm back}^{\rm prop}\right)+\left(\mu_{\rm mol}^{\rm old}-\mu_{\rm mol}^{\rm prop}\right)\sum_{n=1}^{N} b^n\mathbf{PSF}\left(x_k^n,y_k^n,z_k^n\right)\right)\right)\right]$$
$$\times\left(\frac{\mu_{\rm mol}^{\rm old}}{\mu_{\rm mol}^{\rm prop}}\right)^{2\alpha_{\rm mol}^{\rm prop}-\alpha_{\rm mol}}\exp\left(\frac{\mu_{\rm mol}^{\rm old}-\mu_{\rm mol}^{\rm prop}}{\beta_{\rm mol}}+\alpha_{\rm mol}^{\rm prop}\left(\frac{\mu_{\rm mol}^{\rm prop}}{\mu_{\rm mol}^{\rm old}}-\frac{\mu_{\rm mol}^{\rm old}}{\mu_{\rm mol}^{\rm prop}}\right)\right)$$
$$\times\left(\frac{\mu_{\rm back}^{\rm old}}{\mu_{\rm back}^{\rm prop}}\right)^{2\alpha_{\rm back}^{\rm prop}-\alpha_{\rm back}}\exp\left(\frac{\mu_{\rm back}^{\rm old}-\mu_{\rm back}^{\rm prop}}{\beta_{\rm back}}+\alpha_{\rm back}^{\rm prop}\left(\frac{\mu_{\rm back}^{\rm prop}}{\mu_{\rm back}^{\rm old}}-\frac{\mu_{\rm back}^{\rm old}}{\mu_{\rm back}^{\rm prop}}\right)\right). \qquad \text{Supplementary Equation 79}$$

We should emphasize, due to the weakness of the extended Kalman filter as compared to the unscented Kalman filter, we consider the background photon emission rate is fixed for EKF. So, in this case we update the molecular brightness $\mu_{\rm mol}$ only by sampling from the corresponding conditional $p(\mu_{\rm mol}|D,\{q^n,b^n,\overline{x}^n,\overline{y}^n,\overline{z}^n\}_n,\overline{w})$, which simplifies to $p(\mu_{\rm mol}|\{b^n,\overline{x}^n,\overline{y}^n,\overline{z}^n\}_n,\overline{w})$. Again, we carry over this sampling using a Metropolis-Hastings update where proposals for $\mu_{\rm mol}$ are computed according to

$$\mu_{\rm mol}^{\rm prop}\sim\mathbf{Gamma}\left(\alpha_{\rm mol}^{\rm prop},\frac{\mu_{\rm mol}^{\rm old}}{\alpha_{\rm mol}^{\rm prop}}\right) \qquad \text{Supplementary Equation 80}$$

and the acceptance ration will reduces to

$$r_\mu = \prod_{k=1}^{K}\left[\left(\frac{\mu_{\rm back}+\mu_{\rm mol}^{\rm prop}\sum_{n=1}^{N} b^n\mathbf{PSF}\left(x_k^n,y_k^n,z_k^n\right)}{\mu_{\rm back}+\mu_{\rm mol}^{\rm old}\sum_{n=1}^{N} b^n\mathbf{PSF}\left(x_k^n,y_k^n,z_k^n\right)}\right)^{w_k} \times \exp\left(\left(t_k-t_{k-1}\right)\left(\left(\mu_{\rm mol}^{\rm old}-\mu_{\rm mol}^{\rm prop}\right)\sum_{n=1}^{N} b^n\mathbf{PSF}\left(x_k^n,y_k^n,z_k^n\right)\right)\right)\right]$$
$$\times\left(\frac{\mu_{\rm mol}^{\rm old}}{\mu_{\rm mol}^{\rm prop}}\right)^{2\alpha_{\rm mol}^{\rm prop}-\alpha_{\rm mol}}\exp\left(\frac{\mu_{\rm mol}^{\rm old}-\mu_{\rm mol}^{\rm prop}}{\beta_{\rm mol}}+\alpha_{\rm mol}^{\rm prop}\left(\frac{\mu_{\rm mol}^{\rm prop}}{\mu_{\rm mol}^{\rm old}}-\frac{\mu_{\rm mol}^{\rm old}}{\mu_{\rm mol}^{\rm prop}}\right)\right). \qquad \text{Supplementary Equation 81}$$

Supplementary Note 5: Extension for multiple diffusive species

In the case of more than one diffusive species, we can readily modify the model to capture multiple diffusion coefficients. To show that our method can be extended, we consider two diffusive species. Namely, the extended formulation is

$$ {}_1D \sim \mathbf{InvGamma}\left(\alpha_D, \beta_D\right) \quad \text{Supplementary Equation 82} $$
$$ {}_2D \sim \mathbf{InvGamma}\left(\alpha_D, \beta_D\right) \quad \text{Supplementary Equation 83} $$

$$ \mu_{\text{mol}} \sim \mathbf{Gamma}\left(\alpha_{\text{mol}}, \beta_{\text{mol}}\right) \quad \text{Supplementary Equation 84} $$
$$ \mu_{\text{back}} \sim \mathbf{Gamma}\left(\alpha_{\text{back}}, \beta_{\text{back}}\right) \quad \text{Supplementary Equation 85} $$

$$ {}_1q^n \sim \mathbf{Beta}\left(\frac{\alpha_q}{{}_1N}, \beta_q \frac{{}_1N-1}{{}_1N}\right) \quad \text{Supplementary Equation 86} $$
$$ {}_1b^n|{}_1q^n \sim \mathbf{Bernoulli}({}_1q^n) \quad \text{Supplementary Equation 87} $$

$$ {}_2q^n \sim \mathbf{Beta}\left(\frac{\alpha_q}{{}_2N}, \beta_q \frac{{}_2N-1}{{}_2N}\right) \quad \text{Supplementary Equation 88} $$
$$ {}_2b^n|{}_2q^n \sim \mathbf{Bernoulli}({}_2q^n) \quad \text{Supplementary Equation 89} $$

$$ {}_1x_1^n \sim \mathbf{SymNormal}\left(\mu_{\text{xy}}, \sigma^2_{\text{xy}}\right) \quad \text{Supplementary Equation 90} $$
$$ {}_1y_1^n \sim \mathbf{SymNormal}\left(\mu_{\text{xy}}, \sigma^2_{\text{xy}}\right) \quad \text{Supplementary Equation 91} $$
$$ {}_1z_1^n \sim \mathbf{SymNormal}\left(\mu_{\text{z}}, \sigma^2_{\text{z}}\right) \quad \text{Supplementary Equation 92} $$

$$ {}_1x_k^n|{}_1x_{k-1}^n, {}_1D \sim \mathbf{Normal}\left({}_1x_{k-1}^n, 2(t_k - t_{k-1}){}_1D\right), \qquad k = 2, \ldots, K \quad \text{Supplementary Equation 93} $$
$$ {}_1y_k^n|{}_1y_{k-1}^n, {}_1D \sim \mathbf{Normal}\left({}_1y_{k-1}^n, 2(t_k - t_{k-1}){}_1D\right), \qquad k = 2, \ldots, K \quad \text{Supplementary Equation 94} $$
$$ {}_1z_k^n|{}_1z_{k-1}^n, {}_1D \sim \mathbf{Normal}\left({}_1z_{k-1}^n, 2(t_k - t_{k-1}){}_1D\right), \qquad k = 2, \ldots, K \quad \text{Supplementary Equation 95} $$

$$ {}_2x_1^n \sim \mathbf{SymNormal}\left(\mu_{\text{xy}}, \sigma^2_{\text{xy}}\right) \quad \text{Supplementary Equation 96} $$
$$ {}_2y_1^n \sim \mathbf{SymNormal}\left(\mu_{\text{xy}}, \sigma^2_{\text{xy}}\right) \quad \text{Supplementary Equation 97} $$
$$ {}_2z_1^n \sim \mathbf{SymNormal}\left(\mu_{\text{z}}, \sigma^2_{\text{z}}\right) \quad \text{Supplementary Equation 98} $$

$$ {}_2x_k^n|{}_2x_{k-1}^n, {}_2D \sim \mathbf{Normal}\left({}_2x_{k-1}^n, 2(t_k - t_{k-1}){}_2D\right), \qquad k = 2, \ldots, K \quad \text{Supplementary Equation 99} $$
$$ {}_2y_k^n|{}_2y_{k-1}^n, {}_2D \sim \mathbf{Normal}\left({}_2y_{k-1}^n, 2(t_k - t_{k-1}){}_2D\right), \qquad k = 2, \ldots, K \quad \text{Supplementary Equation 100} $$
$$ {}_2z_k^n|{}_2z_{k-1}^n, {}_2D \sim \mathbf{Normal}\left({}_2z_{k-1}^n, 2(t_k - t_{k-1}){}_2D\right), \qquad k = 2, \ldots, K \quad \text{Supplementary Equation 101} $$

$$ w_k|\{{}_1x_k^n, {}_1y_k^n, {}_1z_k^n, {}_2x_k^n, {}_2y_k^n, {}_2z_k^n, {}_1b^n, {}_2b^n\}_n, \mu_{\text{mol}}, \mu_{\text{back}} \sim \mathbf{Poisson}\left(\mu_k\right), \qquad k = 1, \ldots, K \quad \text{Supplementary Equation 102} $$

$$ \mu_k = (t_k - t_{k-1})\left(\mu_{\text{back}} + \mu_{\text{mol}} \sum_n {}_1b^n \text{PSF}({}_1x_k^n, {}_1y_k^n, {}_1z_k^n) + \mu_{\text{mol}} \sum_n {}_2b^n \text{PSF}({}_2x_k^n, {}_2y_k^n, {}_2z_k^n)\right) \quad \text{Supplementary Equation 103} $$

where pre-scripts 1 and 2 are used to distinguish the two species. A graphical summary is show on Supplementary Figure 16. We used this formulation for the estimates shown on Supplementary Figure 12 and Supplementary Figure 13.

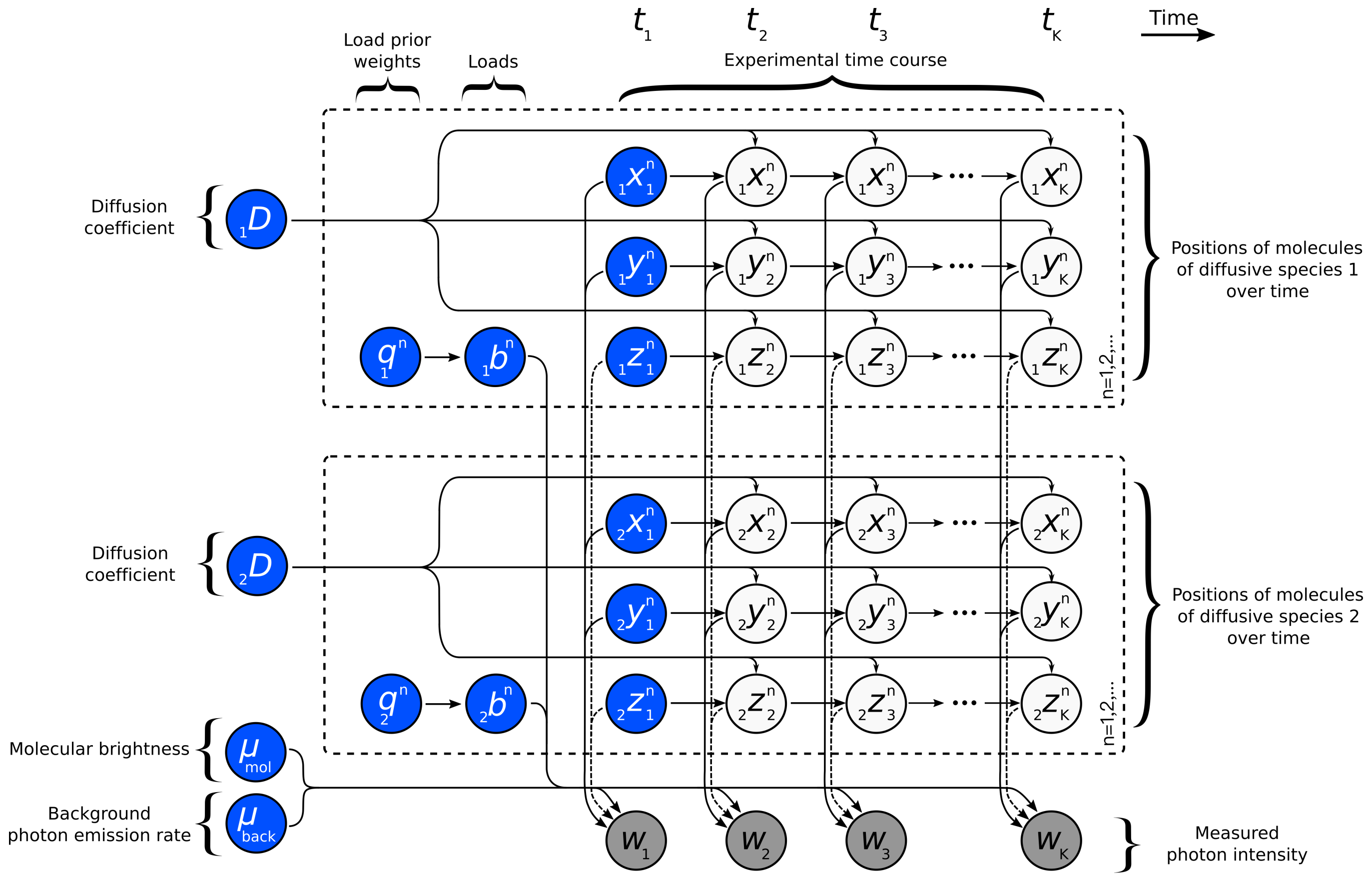

Supplementary Figure 16. Graphical summary of the framework capturing two independent diffusion coefficients. A multi-species population of model molecules, labeled by $n = 1, 2, \ldots$, evolves during the measurement period which is marked by $k = 1, 2, \ldots, K$. Here, ${}_1x_k^n$, ${}_1y_k^n$ and ${}_1z_k^n$ denote the location of molecule n at time t_k of species 1, ${}_2x_k^n$, ${}_2y_k^n$ and ${}_2z_k^n$ denote the location of molecule n at time t_k of species 2, μ_{mol} and μ_{back} denote molecular brightness and background photon emission rates. The diffusion coefficient ${}_1D$ and ${}_2D$ determine the evolution of the molecule locations of species 1 and 2 which, in turn, determine the instantaneous photon emission rates and ultimately the recorded photon intensities w_k. Load variables ${}_1b^n$ and ${}_2b^n$, with prior weights ${}_1q^n$ and ${}_2q^n$, respectively, are introduced to model molecule populations of the two species of a priori unknown sizes. Following common machine learning convention, the measurements w_k are dark shaded. Additionally, model variables requiring prior probability distributions are highlighted in blue.

Supplementary Note 6: Summary of notation, abbreviations, parameters and other options

Supplementary Table 2. Summary of notation

Description	Variable	Units
Diffusion coefficient	D	$\mu m^2 s^{-1}$
α parameter of the diffusion coefficient prior	α_D	-
β parameter of the diffusion coefficient prior	β_D	$\mu m^2 s^{-1}$
Total time trace duration	T_{total}	s
Molecular brightness at the center of the confocal volume	μ_{mol}	photons s^{-1}
α parameter of the molecular brightness's prior	α_{mol}	-
β parameter of the molecular brightness's prior	β_{mol}	photons s^{-1}
Proposal parameter of the molecule photon emission rate	$\alpha_{\text{mol}}^{\text{prop}}$	-
Emission rate of molecule n at time t_k	μ_k^n	photons s^{-1}
Combined photon emission rates of all molecules at time t_k	μ_k	photons s^{-1}
Background photon emission rate	μ_{back}	photons s^{-1}
α parameter of the background photon emission rate's prior	α_{back}	-
β parameter of the background photon emission rate's prior	β_{back}	photons s^{-1}
Proposal parameter of the background photon emission rate	$\alpha_{\text{back}}^{\text{prop}}$	-
Minor semi-axis of confocal PSF (focal plane)	ω_{xy}	μm
Major semi-axis of confocal 3DG PSF (optical axis)	ω_{z}	μm
Major semi-axis of confocal 2DG-L PSF (optical axis)	z_{R}	μm
Laser wavelength	λ_{exc}	μm
Numerical aperture	NA	-
Solution refractive index	n_{sol}	-
Location of sigma points	x^{sn}	μm
Location of molecule n at time t_k in x-coordinate	x_k^n	μm
Location of molecule n at time t_k in y-coordinate	y_k^n	μm
Location of molecule n at time t_k in z-coordinate	z_k^n	μm
Recorded photon intensity at time t_k	w_k	photons
Load variable for molecule n	b^n	-
Prior weight for b_n	q^n	-
α parameter of prior weight q^n	α_q	-
β parameter of prior weight q^n	β_q	-
Upper bound for the number of model molecules	N	-
Mean value of initial molecule position's prior in the xy-plane	μ_{xy}	μm
Mean value of initial molecule position's prior on the z-axis	μ_{z}	μm
Variance of the initial molecule position's prior in the xy-plane	σ_{xy}	μm
Variance of the initial molecule position's prior on the z-axis	σ_{z}	μm
Normalized distance of molecule n at time t_k	d_k^n	-
Normalized distance for the definition of effective volume	ℓ	-
Effective volume	V_ℓ	μm^3
Heaviside function	H	-
Periodic boundary in the xy-plane (focal plane)	L_{xy}	μm
Periodic boundary on the z-axis (optical axis)	L_{z}	μm
Convergence threshold	ϵ_{thr}	$\mu m^2 s^{-1}$
Bin threshold	ϵ_{bin}	photons

Supplementary Table 3. List of abbreviations

Phrase	Abbreviation
Fluorescence correlation spectroscopy	FCS
Point spread function	PSF
Three dimensional Gaussian	3DG
Two dimensional Gaussian-Lorentzian	2DGL
Two dimensional Gaussian-Cylindrical	2DGC
Forward filtering backward sampling	FFBS
Markov chain Monte Carlo	MCMC
Graphical user interface	GUI
Excitation profile	EXC
Collection efficiency function	CEF

Supplementary Table 4. Probability distributions used and their densities

Distribution	Notation	Probability density function	Mean value	Variance/Covariance
Normal	$\textbf{Normal}(\mu, \sigma^2)$	$\frac{1}{\sqrt{2\pi\sigma^2}} e^{-\frac{(x-\mu)^2}{2\sigma^2}}$	μ	σ^2
Symmetric Normal	$\textbf{SymNormal}(\mu, \sigma^2)$	$\frac{1}{2}\frac{e^{-\frac{(x+\mu)^2}{2\sigma^2}}}{\sqrt{2\pi\sigma^2}} + \frac{1}{2}\frac{e^{-\frac{(x-\mu)^2}{2\sigma^2}}}{\sqrt{2\pi\sigma^2}}$	0	$\mu^2 + \sigma^2$
Bivariate Normal	$\textbf{BNormal}(\mu, \Sigma)$	$\frac{1}{2\pi\sqrt{\lvert\Sigma\rvert}} e^{-\frac{1}{2}(x-\mu)^T \Sigma^{-1}(x-\mu)}$	μ	Σ
Poisson	$\textbf{Poisson}(\lambda)$	$\frac{\lambda^x e^{-\lambda}}{x!}$	λ	λ
Gamma	$\textbf{Gamma}(\alpha, \beta)$	$\frac{1}{\Gamma(\alpha)\beta^\alpha} x^{\alpha-1} e^{-\frac{x}{\beta}}$	$\alpha\beta$	$\alpha\beta^2$
Inverse Gamma	$\textbf{InvGamma}(\alpha, \beta)$	$\frac{\beta^\alpha}{\Gamma(\alpha)} x^{-\alpha-1} e^{-\frac{\beta}{x}}$	$\frac{\beta}{\alpha-1}$	$\frac{\beta^2}{(\alpha-1)^2(\alpha-2)}$
Beta	$\textbf{Beta}(\alpha, \beta)$	$\frac{\Gamma(\alpha+\beta)}{\Gamma(\alpha)\Gamma(\beta)} x^{\alpha-1}(1-x)^{\beta-1}$	$\frac{\alpha}{\alpha+\beta}$	$\frac{\alpha\beta}{(\alpha+\beta)^2(\alpha+\beta+1)}$
Bernoulli	$\textbf{Bernoulli}(q)$	$(q-1)\delta_0(x) + q\delta_1(x)$	q	$q(1-q)$

Here, the corresponding random variables are denoted by x.

Supplementary Table 5. Parameter values used in the generation of the synthetic traces

	PSF	L_{xy}	L_{z}	ω_{xy}	$\omega_{\text{z}}, z_{\text{R}}$	N	D	μ_{mol}	μ_{back}	T_{total}	δt
Units		µm	µm	µm	µm	-	$\mu\text{m}^2\text{s}^{-1}$	photons s^{-1}	photons s^{-1}	s	s
Fig. 1(a)	3DG	2	3	0.30	1.50	10	10	5×10^4	10^3	0.1	10^{-4}
Fig. 1(c)	3DG	2	3	0.30	1.50	100	10	5×10^4	10^3	0.1	10^{-4}
Fig. 2(a)	3DG	2,2,2 ,2,4	3,3,3 ,3,7	0.30	1.50	$10^2, 10^2, 10^2$ $, 10^2, 10^3$	$10^{-2}, 10^{-1}, 1$ $, 10, 10^2$	5×10^4	10^3	0.1	10^{-4}
Fig. 2(c)	3DG	2	3	0.30	1.50	150	10	5×10^4	10^3	10	10^{-4}
Fig. 3(a-c)	3DG	2	3	0.30	1.50	150	10	$10^5, 5{\times}10^4, 10^4$	10^3	0.1	10^{-4}
Supplementary Figure 3	3DG	2	3	0.30	1.50	50	10	5×10^4	10^3	0.1	10^{-4}
Supplementary Figure 4(a)	3DG	2	3	0.30	1.50	50	10	5×10^4	10^3	100	10^{-4}
Supplementary Figure 5(a)	3DG	2	3	0.30	1.50	50	10	5×10^4	10^3	0.1	10^{-4}
Supplementary Figure 5(b)	2DGL	2	3	0.30	1.50	50	10	5×10^4	10^3	0.1	10^{-4}
Supplementary Figure 5(c)	2DGC	2	3	0.30	-	50	10	5×10^4	10^3	0.1	10^{-4}
Supplementary Figure 12(a)	3DG	2	3	0.30	1.50	20, 20	1,10	5×10^4	10^3	1	10^{-4}

Choices are listed according to figures.

Supplementary Table 6. Parameter values used in the analyses of the traces

	PSF	ω_{xy}	ω_z,z_R	N	α_D	β_D	α_{mol}	β_{mol}	α_{mol}^{prop}	α_{back}	β_{back}	α_{back}^{prop}	α_q	β_q	μ_{xy}	μ_z	σ_{xy}^2	σ_z^2	ϵ_{thr}	ϵ_{bin}
Units		µm	µm	-	-	µm^{2}s^{-1}	-	photons s^{-1}	-	-	photons s^{-1}	-	-	-	µm	µm	µm^2	µm^2	µm^{2}s^{-1}	photons
Fig. 1(a)	3DG	0.30	1.50	50	1	1	2	10^4	10^3	2	500	10^3	1	1	0.2	0.2	2	2	0.1	4
Fig. 1(c)	3DG	0.30	1.50	50	1	1	2	10^4	10^3	2	500	10^3	1	1	0.2	0.2	2	2	0.1	4
Fig. 2(a)	3DG	0.30	1.50	50	1	1	2	10^4	10^3	2	500	10^3	1	1	0.2	0.2	2	2	0.1	4
Fig. 2(b)	3DG	0.30	1.50	50	1	1	2	10^4	10^3	2	500	10^3	1	1	0.2	0.2	2	2	0.1	4
Fig. 3	3DG	0.30	1.50	50	1	1	2	10^4	10^3	2	500	10^3	1	1	0.2	0.2	2	2	0.1	4
Fig. 4(c)	2DGL	0.40	-	50	1	1	2	10^4	10^3	2	500	10^3	1	1	0.2	0.2	2	2	0.1	4
Fig. 5	3DG	0.27	4.51	50	1	1	2	10^4	10^3	2	500	10^3	1	1	0.2	0.2	2	2	0.1	4
Fig. 6	3DG	0.27	4.51	50	1	1	2	10^4	10^3	2	500	10^3	1	1	0.2	0.2	2	2	0.1	4
Fig. 7(a)	3DG	0.27	4.51	50	1	1	2	10^4	10^3	2	500	10^3	1	1	0.2	0.2	2	2	0.1	4
Fig. 7(b)	3DG	0.27	4.51	50	1	1	2	10^4	10^3	2	500	10^3	1	1	0.2	0.2	2	2	0.1	4
Fig. 7(c)	3DG	0.27	4.51	50	1	1	2	10^4	10^3	2	500	10^3	1	1	0.2	0.2	2	2	0.1	4
Supplementary Figure 3	3DG	0.30	1.50	50	1	1	2	10^4	10^3	2	500	10^3	1	1	0.2	0.2	2	2	0.1	4
Supplementary Figure 4	3DG	0.30	1.50	50	1	1	2	10^4	10^3	2	500	10^3	1	1	0.2	0.2	2	2	0.1	4
Supplementary Figure 5(a)	3DG	0.30	1.50	50	1	1	2	10^4	10^3	2	500	10^3	1	1	0.2	0.2	2	2	0.1	4
Supplementary Figure 5(b)	2DGL	0.30	1.50	50	1	1	2	10^4	10^3	2	500	10^3	1	1	0.2	0.2	2	2	0.1	4
Supplementary Figure 5(c)	2DGC	0.30	-	50	1	1	2	10^4	10^3	2	500	10^3	1	1	0.2	0.2	2	2	0.1	4
Supplementary Figure 8	3DG	0.27	4.51	50	1	1	2	10^4	10^3	2	500	10^3	1	1	0.2	0.2	2	2	0.1	4
Supplementary Figure 9	3DG	0.27	4.51	50	1	1	2	10^4	10^3	2	500	10^3	1	1	0.2	0.2	2	2	0.1	4
Supplementary Figure 10	2DGL	0.42	-	50	1	1	2	10^4	10^3	2	500	10^3	1	1	0.2	0.2	2	2	0.1	4
Supplementary Figure 12	3DG	0.3	1.5	50	1	1	2	10^4	10^3	2	500	10^3	1	1	0.2	0.2	2	2	0.1	4

Choices are listed according to figures.

Supplementary References

[1] Berg, H. C. *Random walks in biology* (Princeton University Press, 1993).

[2] Hida, T. Brownian motion. In *Brownian Motion*, 44–113 (Springer, 1980).

[3] Einstein, A. Über die von der molekularkinetischen theorie der wärme geforderte bewegung von in ruhenden flüssigkeiten suspendierten teilchen. *Annalen der physik* **322**, 549–560 (1905).

[4] Cheng, N.-S. Formula for the viscosity of a glycerol- water mixture. *Industrial & engineering chemistry research* **47**, 3285–3288 (2008).

[5] Cohen, L. Convolution, filtering, linear systems, the wiener-khinchin theorem: generalizations. In *Advanced Signal Processing Algorithms, Architectures, and Implementations III*, vol. 1770, 378–394 (International Society for Optics and Photonics, 1992).

[6] Widengren, J., Mets, U. & Rigler, R. Fluorescence correlation spectroscopy of triplet states in solution: a theoretical and experimental study. *The Journal of Physical Chemistry* **99**, 13368–13379 (1995).

[7] Enderlein, J., Gregor, I., Patra, D. & Fitter, J. Statistical analysis of diffusion coefficient determination by fluorescence correlation spectroscopy. *Journal of fluorescence* **15**, 415–422 (2005).

[8] Rigler, R. & Elson, E. S. *Fluorescence correlation spectroscopy: theory and applications*, vol. 65 (Springer Science & Business Media, 2012).

[9] Guo, S.-M. *et al.* Bayesian approach to the analysis of fluorescence correlation spectroscopy data ii: application to simulated and in vitro data. *Analytical chemistry* **84**, 3880–3888 (2012).

[10] Von Toussaint, U. Bayesian inference in physics. *Reviews of Modern Physics* **83**, 943 (2011).

[11] Tavakoli, M., Taylor, J. N., Li, C.-B., Komatsuzaki, T. & Pressé, S. *Single Molecule Data Analysis: An Introduction*, chap. 4, 205–305 (John Wiley & Sons, 2017).

[12] Sivia, D. & Skilling, J. *Data analysis: a Bayesian tutorial* (OUP Oxford, 2006).

[13] Enderlein, J. & Ambrose, W. P. Optical collection efficiency function in single-molecule detection experiments. *Applied optics* **36**, 5298–5302 (1997).

[14] Chen, Y., Müller, J. D., Ruan, Q. & Gratton, E. Molecular brightness characterization of egfp in vivo by fluorescence fluctuation spectroscopy. *Biophysical journal* **82**, 133–144 (2002).

[15] Wilson, T. *et al. Confocal microscopy*, vol. 426 (Academic press London, 1990).

[16] Pawley, J. *Handbook of biological confocal microscopy* (Springer Science & Business Media, 2010).

[17] Masters, B. R. & So, P. *Handbook of biomedical nonlinear optical microscopy* (Oxford University Press, 2008).

[18] Schwille, P. Fluorescence correlation spectroscopy and its potential for intracellular applications. *Cell biochemistry and biophysics* **34**, 383–408 (2001).

[19] Wolf, E. Electromagnetic diffraction in optical systems. i. an integral representation of the image field. In *Proceedings of the Royal Society of London A: Mathematical, Physical and Engineering Sciences*, vol. 253, 349–357 (The Royal Society, 1959).

[20] Richards, B. & Wolf, E. Electromagnetic diffraction in optical systems. ii. structure of the image field in an aplanatic system. In *Proceedings of the Royal Society of London A: Mathematical, Physical and Engineering Sciences*, vol. 253, 358–379 (The Royal Society, 1959).

[21] Rigler, R., Mets, Ü., Widengren, J. & Kask, P. Fluorescence correlation spectroscopy with high count rate and low background: analysis of translational diffusion. *European Biophysics Journal* **22**, 169–175 (1993).

[22] Zhang, B., Zerubia, J. & Olivo-Marin, J.-C. Gaussian approximations of fluorescence microscope point-spread function models. *Applied Optics* **46**, 1819–1829 (2007).

[23] Berland, K. M., So, P. & Gratton, E. Two-photon fluorescence correlation spectroscopy: method and application to the intracellular environment. *Biophysical Journal* **68**, 694–701 (1995).

[24] Dertinger, T. *et al.* Two-focus fluorescence correlation spectroscopy: A new tool for accurate and absolute diffusion measurements. *ChemPhysChem* **8**, 433–443 (2007).

[25] Siegman, A. E. Lasers (mill valley, ca (1986).

[26] Blom, H. & Björk, G. Lorentzian spatial intensity distribution in one-photon fluorescence correlation spectroscopy. *Applied optics* **48**, 6050–6058 (2009).

[27] Born, M. & Wolf, E. *Principles of optics: electromagnetic theory of propagation, interference and diffraction of light* (Elsevier, 2013).

[28] Gibson, S. F. & Lanni, F. Experimental test of an analytical model of aberration in an oil-immersion objective lens used in three-dimensional light microscopy. *JOSA A* **9**, 154–166 (1992).

[29] Buschmann, V., Krämer, B., Koberling, F., Macdonald, R. & Rättinger, S. Quantitative fcs: determination of the confocal volume by fcs and bead scanning with the microtime 200. *Application Note PicoQuant GmbH, Berlin* (2009).

[30] Haile, J., Johnston, I., Mallinckrodt, A. J., McKay, S. *et al.* Molecular dynamics simulation: elementary methods. *Computers in Physics* **7**, 625–625 (1993).

[31] Jazani, S., Sgouralis, I. & Pressé, S. A method for single molecule tracking using a conventional single-focus confocal setup. *The Journal of Chemical Physics* **150**, 123320 (2019).

[32] Anscombe, F. J. The transformation of poisson, binomial and negative-binomial data. *Biometrika* **35**, 246–254 (1948).

[33] Paisley, J. & Jordan, M. I. A constructive definition of the beta process. *arXiv preprint arXiv:1604.00685* (2016).

[34] Broderick, T., Jordan, M. I., Pitman, J. *et al.* Beta processes, stick-breaking and power laws. *Bayesian analysis* **7**, 439–476 (2012).

[35] Paisley, J. & Carin, L. Nonparametric factor analysis with beta process priors. In *Proceedings of the 26th Annual International Conference on Machine Learning*, 777–784 (ACM, 2009).

[36] Al Labadi, L. & Zarepour, M. On approximations of the beta process in latent feature models: Point processes approach. *Sankhya A* 1–21.

[37] Robert, C. & Casella, G. *Introducing Monte Carlo Methods with R* (Springer Science & Business Media, 2009).

[38] Gelman, A. *et al. Bayesian data analysis*, vol. 2 (CRC press Boca Raton, FL, 2014).

[39] Liu, H. & Motoda, H. *Computational methods of feature selection* (CRC Press, 2007).

[40] Sgouralis, I. & Pressé, S. An introduction to infinite hmms for single-molecule data analysis. *Biophysical Journal* **112**, 2021–2029 (2017).

[41] Cappé, O., Moulines, E. & Rydén, T. Inference in hidden markov models. In *Proceedings of EUSFLAT Conference*, 14–16 (Springer, 2009).

[42] Rydén, T. *et al.* Em versus markov chain monte carlo for estimation of hidden markov models: A computational perspective. *Bayesian Analysis* **3**, 659–688 (2008).

[43] Rabiner, L. & Juang, B. An introduction to hidden markov models. *ieee assp magazine* **3**, 4–16 (1986).

[44] Bishop, C. M. *Pattern recognition and machine learning* (springer, 2006).

[45] Rabiner, L. R. A tutorial on hidden markov models and selected applications in speech recognition. *Proceedings of the IEEE* **77**, 257–286 (1989).

[46] Scott, S. L. Bayesian methods for hidden markov models: Recursive computing in the 21st century. *Journal of the American Statistical Association* **97**, 337–351 (2002).

[47] Byron, M. Y., Shenoy, K. V. & Sahani, M. Derivation of extended kalman filtering and smoothing equations. *Technical report, Department of Electrical Engineering, Stanford University* **19**, 25 (2004).
[48] Wan, E. A. & Van Der Merwe, R. The unscented kalman filter for nonlinear estimation. In *Adaptive Systems for Signal Processing, Communications, and Control Symposium 2000. AS-SPCC. The IEEE 2000*, 153–158 (Ieee, 2000).
[49] Menegaz, H. M., Ishihara, J. Y., Borges, G. A. & Vargas, A. N. A systematization of the unscented kalman filter theory. *IEEE Transactions on automatic control* **60**, 2583–2598 (2015).
[50] Stoer, J. & Bulirsch, R. *Introduction to numerical analysis*, vol. 12 (Springer Science & Business Media, 2013).
[51] Kalman, R. E. A new approach to linear filtering and prediction problems. *Journal of Basic Engineering* **82**, 35–45 (1960).
[52] Kalman, R. E. & Bucy, R. S. New results in linear filtering and prediction theory. *Journal of Basic Engineering* **83**, 95–108 (1961).
[53] Sorenson, H. W. Kalman filtering techniques. In *Advances in Control Systems*, vol. 3, 219–292 (Elsevier, 1966).
[54] Frühwirth, R. Application of kalman filtering to track and vertex fitting. *Nuclear Instruments and Methods in Physics Research Section A: Accelerators, Spectrometers, Detectors and Associated Equipment* **262**, 444–450 (1987).
[55] Ljung, L. Asymptotic behavior of the extended kalman filter as a parameter estimator for linear systems. *IEEE Transactions on Automatic Control* **24**, 36–50 (1979).
[56] Song, T. & Speyer, J. A stochastic analysis of a modified gain extended kalman filter with applications to estimation with bearings only measurements. *IEEE Transactions on Automatic Control* **30**, 940–949 (1985).
[57] Hoshiya, M. & Saito, E. Structural identification by extended kalman filter. *Journal of engineering mechanics* **110**, 1757–1770 (1984).
[58] Štecha, J. & Havlena, V. Unscented kalman filter revisited–hermite-gauss quadrature approach. In *Information Fusion (FUSION), 2012 15th International Conference on*, 495–502 (IEEE, 2012).
[59] Sarkka, S. On unscented kalman filtering for state estimation of continuous-time nonlinear systems. *IEEE Transactions on automatic control* **52**, 1631–1641 (2007).
[60] Julier, S. J. & Uhlmann, J. K. New extension of the kalman filter to nonlinear systems. In *Signal Processing, sensor fusion, and target recognition VI*, vol. 3068, 182–194 (International Society for Optics and Photonics, 1997).
[61] Heiss, F. & Winschel, V. Likelihood approximation by numerical integration on sparse grids. *journal of Econometrics* **144**, 62–80 (2008).
